# Functional and effective EEG connectivity patterns in Alzheimer’s disease and mild cognitive impairment: a systematic review

**DOI:** 10.3389/fnagi.2025.1496235

**Published:** 2025-02-12

**Authors:** Elizabeth R. Paitel, Christian B. D. Otteman, Mary C. Polking, Henry J. Licht, Kristy A. Nielson

**Affiliations:** ^1^Aging, Imaging, and Memory Laboratory, Department of Psychology, Marquette University, Milwaukee, WI, United States; ^2^Department of Neurology, Medical College of Wisconsin, Milwaukee, WI, United States

**Keywords:** connectivity, Alzheimer’s disease, mild cognitive impairment, EEG, aging, neurodegeneration, neuroimaging

## Abstract

**Background:**

Alzheimer’s disease (AD) might be best conceptualized as a disconnection syndrome, such that symptoms may be largely attributable to disrupted communication between brain regions, rather than to deterioration within discrete systems. EEG is uniquely capable of directly and non-invasively measuring neural activity with precise temporal resolution; connectivity quantifies the relationships between such signals in different brain regions. EEG research on connectivity in AD and mild cognitive impairment (MCI), often considered a prodromal phase of AD, has produced mixed results and has yet to be synthesized for comprehensive review. Thus, we performed a systematic review of EEG connectivity in MCI and AD participants compared with cognitively healthy older adult controls.

**Methods:**

We searched PsycINFO, PubMed, and Web of Science for peer-reviewed studies in English on EEG, connectivity, and MCI/AD relative to controls. Of 1,344 initial matches, 124 articles were ultimately included in the systematic review.

**Results:**

The included studies primarily analyzed coherence, phase-locked, and graph theory metrics. The influence of factors such as demographics, design, and approach was integrated and discussed. An overarching pattern emerged of lower connectivity in both MCI and AD compared to healthy controls, which was most prominent in the alpha band, and most consistent in AD. In the minority of studies reporting greater connectivity, theta band was most commonly implicated in both AD and MCI, followed by alpha. The overall prevalence of alpha effects may indicate its potential to provide insight into nuanced changes associated with AD-related networks, with the caveat that most studies were during the resting state where alpha is the dominant frequency. When greater connectivity was reported in MCI, it was primarily during task engagement, suggesting compensatory resources may be employed. In AD, greater connectivity was most common during rest, suggesting compensatory resources during task engagement may already be exhausted.

**Conclusion:**

The review highlighted EEG connectivity as a powerful tool to advance understanding of AD-related changes in brain communication. We address the need for including demographic and methodological details, using source space connectivity, and extending this work to cognitively healthy older adults with AD risk toward advancing early AD detection and intervention.

## 1 Introduction

Alzheimer’s disease (AD) is a neurodegenerative disorder characterized by impairment in multiple cognitive domains (e.g., memory, planning, problem solving) and in the ability to complete instrumental activities of daily living (e.g., managing medications, preparing meals; Alzheimer’s Association, 2024). The neuropathological signatures of AD include the accumulation of neurofibrillary tangles and amyloid plaques. Although there is a great deal of attention on amyloid deposition and intervention in AD diagnosis and treatment (Long and Holtzman, 2019; Selkoe and Hardy, 2016), neurofibrillary tangles importantly and disproportionately impact the tracts that underlie communication between different brain regions (Delbeuck et al., 2003; Watanabe et al., 2019; Yu et al., 2021). These patterns, coupled with evidence of structural and functional brain network impairments in AD, have led to the hypothesis that AD is a “disconnection syndrome” (for review see Delbeuck et al., 2003; Stam, 2014; Yu et al., 2021). Specifically, AD symptoms are proposed to result from impaired connectivity between various brain regions and networks, rather than being due to the disruption of discrete neural systems.

Characterizing neural connectivity patterns may be crucial to tracking the development and progression of AD across its different stages. Mild cognitive impairment (MCI), considered a prodromal stage of AD, is characterized by cognitive decline beyond what is typical for healthy aging, but that is insufficient to meet criteria for AD, and without loss of the abilities required to live independently (Alzheimer’s Association, 2022, 2024; Petersen, 2004, 2016). Although the presentation of MCI is heterogeneous, with multiple underlying causes, approximately 10–20% of cases convert to AD every year, with approximately one-third developing AD within five years (Alzheimer’s Association, 2022; Bruscoli and Lovestone, 2004; Petersen et al., 2018; Ward et al., 2013). Thus, it is important to evaluate MCI toward better understanding early AD risk.

Despite increasing focus on the quantification of amyloid plaques and neurofibrillary tangles for AD diagnosis, the neuroimaging of these biomarkers is invasive and extremely expensive, which severely limits its feasibility (Fiandaca et al., 2014; Milà-Alomà et al., 2019). Conversely, electroencephalography (EEG) is a neuroimaging method that is non-invasive, inexpensive, and directly measures neural functioning at a millisecond scale, via summated post-synaptic potentials in real-time (Luck, 2014; Slotnick, 2017). EEG can be used to model the relationship between neural activity in different brain regions, providing information regarding the communication, or connectivity, between those regions, which can only be estimated by other *in vivo* neuroimaging methods. In addition, EEG signals may be deconvolved into their underlying neural oscillations (i.e., rhythms), which have been suggested as a critical component of signal transfer between brain regions (Buzsáki and Watson, 2012; Chapeton et al., 2019; Mulert, 2013; Schnitzler and Gross, 2005). For these reasons, recent international initiatives advocate for increased utilization and study of EEG as a biomarker of AD (Babiloni et al., 2021; Babiloni et al., 2020; Maestú et al., 2019; Paitel et al., 2021).

There are multiple approaches to analyzing connectivity with EEG (cf. Bastos and Schoffelen, 2016; Cao et al., 2022; Chiarion et al., 2023; Sakkalis, 2011; Srinivasan et al., 2007), either via bivariate or multivariate signal relationships. The most common approaches to connectivity are non-directed metrics, which quantify the relationship between the signals, without inferring causation (i.e., one region sending a signal to the other). Directed (i.e., effective) connectivity, on the other hand, seeks to establish a causal relationship, determining the causal flow of information between regions. EEG connectivity is analyzed in either the time or frequency domain, with additional time-frequency approaches gaining popularity in recent years (Chiarion et al., 2023; Morales and Bowers, 2022). The most common time domain metrics are those based in correlations, such as Pearson’s correlations, mutual information, and cross-correlation (Bastos and Schoffelen, 2016; Cao et al., 2022). Frequency domain approaches first decompose the signal into the underlying oscillatory activity, which are then commonly grouped by their fundamental frequency bands: delta (2-4 Hz), theta (4-8 Hz), alpha (8-12 Hz), beta (12-30 Hz), and gamma (30 + Hz). Relationships may then be analyzed between the phase or power of the oscillatory signals. The most common frequency-based approaches include variations of coherence, phase-locking value, and phase-slope index (Bastos and Schoffelen, 2016; Cao et al., 2022). It is important to note that the underlying meaning of connectivity computed from each of these metrics may have different interpretations. For example, it has been suggested that phase-based measures primarily reveal information regarding the timing of activity within neural populations, while power-based metrics are more informative about the quantity or spatial extent of such populations (Cohen, 2014). Thus, the use of different metrics, and even the data processing choices with the same metrics, may contribute to substantial variability across studies.

The existing EEG literature on resting state and task-induced connectivity in AD and MCI is both complex and nuanced, and it has yet to be systematically reviewed. Existing systematic reviews have been selective, focusing on certain connectivity approaches (e.g., magnitude squared coherence, Fischer et al., 2023), frequency bands (e.g., alpha, Lejko et al., 2020), only resting state activity (Babiloni et al., 2016a; Cassani et al., 2018; Teipel et al., 2016; Vecchio et al., 2013), or considering either MCI or AD, but not both (Buzi et al., 2023). While not a systematic review, the most recent paper to review EEG studies in both MCI and AD during task and rest included discussion of multiple EEG approaches, including connectivity studies (Horvath et al., 2018). A recent review from Adebisi and Veluvolu (2023) that covered the years of 2016–2020 evaluated the discriminative ability of EEG connectivity for dementia diagnoses, not specific to MCI or AD, with a methodological focus. Overall, existing reviews are biased toward assessing resting state activity (default-mode network, DMN). They typically report reduced alpha band connectivity in MCI and AD compared to healthy control groups (HC), with some studies pointing to the strongest effects in longer-distance communication, such as between frontal–parietal and frontal-temporal regions (Babiloni et al., 2016a; Fischer et al., 2023; Lejko et al., 2020). Findings in other frequency bands have been inconsistent (Babiloni et al., 2016a; Buzi et al., 2023; Fischer et al., 2023; Horvath et al., 2018). Thus, a comprehensive, up-to-date, and systematic review is timely and important.

The present systematic review summarizes studies comparing EEG connectivity in MCI or AD with healthy older adult controls, including resting state and task-activated studies, across all connectivity metrics. We report the results of studies grouped by connectivity approach, and we highlight overarching patterns both within and across these approaches by diagnostic group relative to controls. The contributions and limitations of demographic factors, methods, and study designs are also discussed. The aim of the present review is to both discern patterns in the rich and complex existing research on EEG connectivity in MCI and AD, as well as to guide future cognitive neuroscience research on the use of EEG as an early AD biomarker.

## 2 Methods

### 2.1 Search strategies

**Figure 1:**
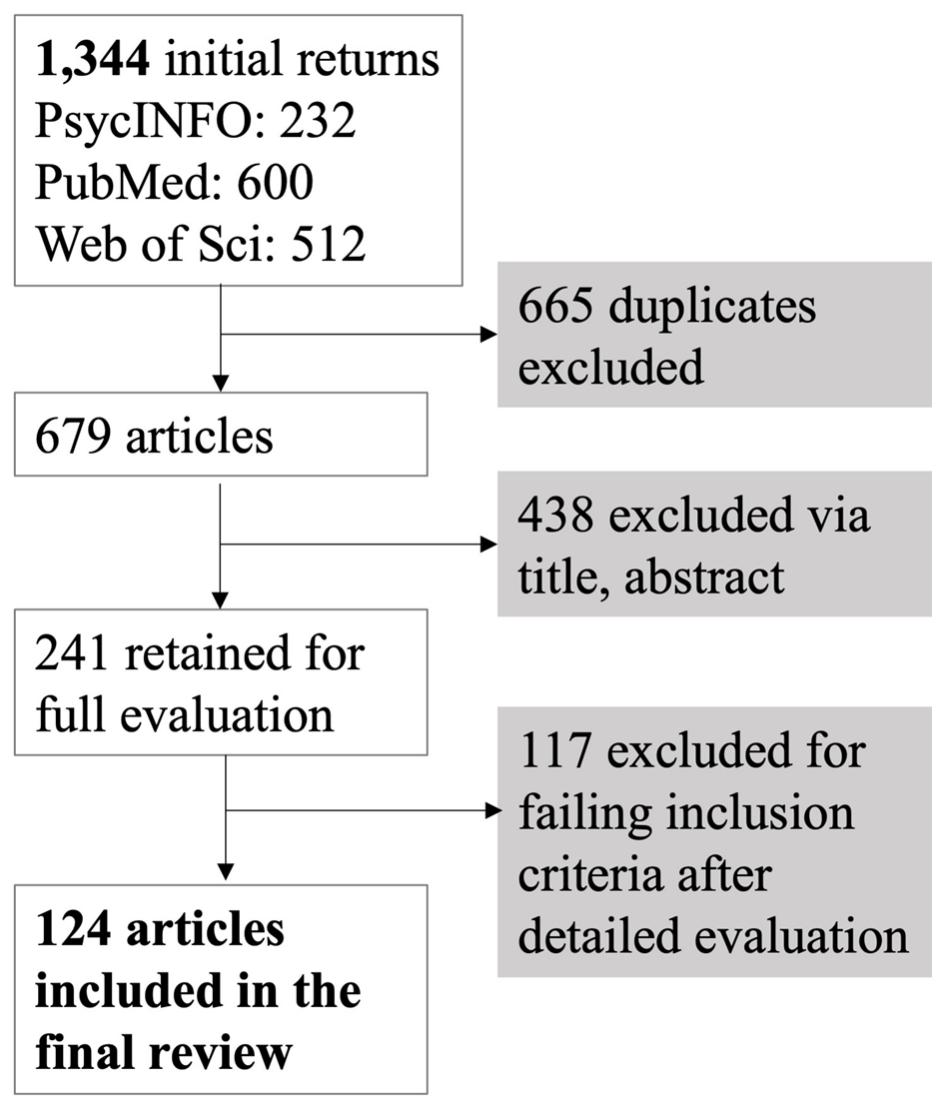
Article selection process for inclusion in the systematic review, consistent with the Preferred Reporting Items for Systematic reviews and Meta-Analyses (PRISMA) guidelines (Page et al., 2021).

Database searches were conducted in PsycINFO, PubMed, and Web of Science, inclusive of all dates from the inception of the databases through February 9, 2023. Given its relevance to risk for AD and previous work indicating the potential importance of neural patterns in asymptomatic participants with risk for AD (Bondi et al., 2005; Filippini et al., 2011; Paitel et al., 2021; Rao et al., 2015; Reuter-Lorenz and Park, 2014; Woodard et al., 2010), we also conducted searches for studies with healthy, cognitively intact participants with genetic AD risk via the Apolipoprotein-E (APOE) ε4 allele (Alzheimer’s Association, 2024; Yu et al., 2014). The search strategy syntax was adjusted for each specific database, but specifically required the keywords [“EEG” AND “connectivity”] AND [“MCI” OR “mild cognitive impairment”] or [“Alzheimer’s”] or [“APOE” OR “Apolipoprotein E”], as well peer-reviewed, published paper type and that they be written in English. The number of articles at each step of the review process is detailed in Figure 1. Notably, returns only considered the role of APOE within cognitively impaired groups (i.e., MCI or AD), rather than in cognitively healthy groups. As such, APOE could not be evaluated separately from MCI and AD.

### 2.2 Article selection

Figure 1 displays the overall process for study inclusion and exclusion, consistent with the Preferred Reporting Items for Systematic reviews and Meta-Analyses (PRISMA) guidelines (Page et al., 2021). Duplicate articles were first deleted, and the remaining returns were then evaluated using the paper title and abstract. These papers were only further examined if the title and/or abstract suggested they: (1) were original, empirical, peer-reviewed studies, (2) analyzed functional or effective connectivity using EEG, (3) employed a sample of participants with MCI or AD, and (4) compared an MCI/AD group with a healthy older adult control group. The 241 studies that passed this initial evaluation were subjected to full article evaluation. Studies excluded at this stage failed inclusion criteria after detailed evaluation for the following reasons: not an original, empirical, peer-reviewed study (*n* = 5); did not include a sample with sporadic, late-onset AD, MCI, or APOE groups (*n* = 12); did not include direct, statistical comparison with a healthy, cognitively intact older adult control group (*n* = 44); did not analyze functional or effective connectivity (*n* = 34); connectivity analyses did not use EEG (*n* = 17); were duplicate articles (*n* = 4); or were unavailable in English (*n* = 1). Note that some excluded papers failed multiple inclusion criteria, but each was recorded under one category for simplicity. These exclusions resulted in a final total of 124 studies to be included in the review.

## 3 Results

The 124 studies included in this review included 35 papers that analyzed an MCI sample (Supplementary Table S1), 56 that examined AD (Supplementary Table S2), and 33 papers that analyzed both MCI and AD samples (shown in both Supplementary Tables S1, S2). These papers included 9,537 participants total (2,279 MCI (24%); 3,603 AD (38%); 3,655 HC (38%)), although there is some duplication of participants that were included in more than one study (e.g., Ruiz-Gómez et al., 2019b; Ruiz-Gómez et al., 2021). Multiple methodological approaches were used to examine connectivity across studies (see Supplementary Table S3), with coherence, other phase-based approaches (which we will refer to as ‘phase-locked’ for brevity), and graph theory approaches most frequently applied. Quality of the included studies was variable; a quality assessment checklist to assess the risk of bias in individual studies adapted from the Newcastle-Ottawa scale (Lejko et al., 2020; Wells et al., 2014) is provided in Table S4. We also note that the vast majority of the studies reviewed were conducted in independently recruited research samples, with little use of large public databases or repositories. Although there was some evidence of overlapping samples between some studies, they analyzed different connectivity metrics and/or different EEG contexts within those samples.

### 3.1 Sample characteristics

#### 3.1.1 Sample size

Group sample sizes ranged widely across studies (HC *n* = 7–135, *M*_HC_ = 28.8, *SD =* 23.4; MCI *n* = 7–154, *M*_MCI_ = 33.0, *SD =* 26.7; AD *n* = 6–318, *M*_AD_ = 40.0, *SD* = 47.8). There was a marginal difference across sample types amongst the studies in the review, *F*(2,283) = 2.95, *p* = 0.054, $\eta^{2}$ = 0.02. Post-hoc contrasts were checked despite the marginal omnibus effect, given the lack of specific hypothesis and exploratory nature of the comparison; sample sizes were comparable between AD and MCI (7.0, *p* = 0.20) and MCI and HC (4.2, *p* = 0.40), with AD samples overall larger than HC samples (11.3, *p* = 0.02).

#### 3.1.2 Age

Mean age was reported in 105 out of 124 studies (i.e., 85%). Seven additional studies reported only the age range or minimum age of the samples. Across studies that reported mean age, there was a significant overall difference amongst sample types, *F*(2,232) = 9.97, *p* < 0.001, $\eta^{2}$ = 0.09. Post-hoc contrasts showed the effect was attributable to younger HC groups (range: 57.4–80.1 years, *M*_HC_ = 69.3, *SD* = 4.8) than MCI groups (range: 61.0–85.5 years, *M*_MCI_ = 71.1, *SD* = 5.1; −1.80, *p* = 0.02) and AD groups (range: 64.0–83.9 years, *M*_AD_ = 72.4, *SD* = 4.0; −3.12, *p* < 0.001). Average age was not significantly different between AD and MCI groups (1.30, *p* = 0.11).

#### 3.1.3 Education

Only 61 out of 124 studies (i.e., 49%) reported average years of education by group. Four additional studies reported education as a binary category (e.g., 5–11 years or > 11 years), and two studies reported only a minimum education level. Across studies reporting mean education, there was a significant difference between sample types, *F*(2,134) = 5.23, *p* = 0.007, $\eta^{2}$ = 0.07. Post-hoc contrasts showed that this difference was due to significantly fewer years of education in the AD groups (range: 3.31–14.14 years, *M*_AD_ = 9.6, *SD* = 2.48) when compared with MCI groups (range: 5.86–15.39, *M*_MCI_ = 11.0, *SD* = 2.49; −1.35, *p* = 0.04) or HC groups (range: 3.36–16.50, *M*_HC_ = 11.2, *SD* = 2.87; −1.87, *p* = 0.005). HC did not significantly differ from MCI (0.53, *p* = 0.37).

#### 3.1.4 Sex

The distribution of female and male participants was reported in 103 out of 124 studies (i.e., 83%). Across these studies, sex distribution varied widely, with percent female ranging from 25–74% in HC (*M*_HC_ = 54.68%, *SD* = 12.04), 24–76% in MCI (*M*_MCI_ = 52.39, *SD* = 12.74), and 20–86% in AD (*M*_AD_ = 57.54%, *SD* = 13.18). A Kruskal-Wallis non-parametric ANOVA showed no overall a significant difference in distribution of females, *H*(2) = 2.37, *p* = 0.31.

#### 3.1.5 MMSE scores

83 out of 124 studies (i.e., 67%) reported average MMSE scores (possible range: 0–30) for their samples (Folstein et al., 1983). Of the 41 papers that lacked MMSE data (see Supplementary Tables S1, S2), four used a different metric, 17 reported MMSE in a manner that could not be accurately tabulated (e.g., ranges, cutoffs, medians, unclear group values), and 13 papers provided no specific cognitive information or only reported that participants “met criteria” for the diagnostic category. For the 83 studies reporting MMSE, there was a notable range of MMSE scores particularly in AD groups (HC: 25.73–30.00, MCI: 20.3–28.41, AD: 9.40–26.30). Moreover, there was a significant overall group difference in MMSE, as expected (*F*(2,181) = 320.15, *p* < 0.001, $\eta^{2}$ = 0.78). Post-hoc contrasts showed that MMSE was significantly lower in AD than in MCI (−5.91, *p* < 0.001, *M*_AD_ = 19.94, *SD* = 3.07, *M*_MCI_ = 25.85, SD = 1.66) and in HC (−8.67, *p* < 0.001, *M*_HC_ = 28.61, *SD* = 0.93), and MCI was lower than HC (−2.76, *p* < 0.001). Notably, MMSE within HC groups was comparable regardless of study sample composition differences (i.e., studies that compared HC with AD, HC with MCI, or all three groups, *F*(2,79) = 0.30, *p =* 0.74, $\eta^{2}$ = 0.008).

### 3.2 Methodological characteristics

#### 3.2.1 Metrics

The papers in this review represented most of the various metrics available for studying EEG connectivity, with some studies applying more than one approach. The primary approaches were coherence (*n* = 41), phase-locked (*n* = 35), and graph theory (*n* = 44), with multiple other less frequently applied approaches also represented (*n* = 34 studies). Importantly, these metrics analyze fundamentally different aspects of neural connectivity, which can contribute to the appearance of variability across studies (Cohen, 2014). Moreover, even within methods, there were computational differences and methodological choices that might contribute to differences in study findings (see Supplementary Table S3). Four methodological factors deserve particular attention. First, only 29% of studies (i.e., 36/124) analyzed connectivity in source space. Instead, the majority of studies did analyses at the electrode level. Sensor-level approaches are limited by the effects of volume conduction, such that multiple EEG sensors record signals from the same underlying brain region, which can result in connectivity results that reflect activity from shared brain regions (Chiarion et al., 2023; Mahjoory et al., 2017; Michel and Brunet, 2019; Schoffelen and Gross, 2009; Van de Steen et al., 2019).

#### 3.2.2 Data segmentation

There was substantial variability across studies in the duration of the data segments used to compute connectivity. For connectivity metrics in the frequency domain, frequency resolution is determined by the number of samples included in the data segments. Durations ranged widely, from 400 milliseconds at 256 Hz (~1,563 samples, ~2.5 Hz resolution; Bagattini et al., 2022) to 40 s at 500 Hz (80,000 samples, ~.025 Hz resolution; Kabbara et al., 2018; but see also Vyšata et al., 2015). Generally, it is recommended to use a time window that includes three cycles at the lowest frequency of interest or at least one second before and after the time window of interest to ensure sufficient frequency resolution (Cohen, 2014). Thus, some of the reviewed samples used too few time points, likely resulting in smearing, which is a distortion (or “blurring”) of the EEG signal that makes it difficult to interpret a signal’s actual frequency (Buzzell et al., 2022; Cohen, 2014).

#### 3.2.3 Measurement context

The studies reviewed primarily analyzed EEG connectivity during the resting state. Indeed, only 19% (i.e., 24/124) of studies analyzed EEG during active task engagement. Eleven of 24 (46%) task-related studies used coherence methods. The tasks used typically tapped memory-related functions (*n* = 16; e.g., digit span, *n*-back, mental arithmetic) or basic sensory-attentional processing (*n* = 7; e.g., visual, auditory, or olfactory oddball paradigms).

#### 3.2.4 Frequency bands

Most of the reviewed studies analyzed connectivity using multiple frequency bands. The most common frequency bands were alpha (*n* = 94), theta (*n* = 87), beta (*n* = 84), and delta (*n* = 72). Gamma activity was less frequently included (*n* = 46), which is likely attributable to greater concerns of EEG artifact within high frequencies (Muthukumaraswamy, 2013). An additional 23 studies analyzed “broadband” EEG signals, using a range that included activity across multiple frequency bands (e.g., 1-100 Hz). Within alpha and beta bands, about half of the studies divided analyses into lower (alpha-1, beta-1) and upper (alpha-2, beta-2) bands. This approach may be advantageous given the relatively wide frequency range within alpha (typically ~8-12 Hz) and beta (~12-30 Hz), relative to delta (~2-4 Hz) and theta (~4-8 Hz), and some evidence of differential patterns by band subdivision (Bazanova and Vernon, 2014; Klimesch, 1999).

### 3.3 Connectivity results

The studies with significant connectivity findings are presented separately for each of the primary connectivity methods, specifically: coherence (Table 1); phase-locked (Table 2); graph theory (Table 3); and various other, less frequently used approaches (e.g., correlational metrics; Table 4). Three studies do not appear in these tables due to purely non-significant findings (Ding et al., 2022; Kim et al., 2018; Núñez et al., 2019); they can be found in Supplementary Table S3.

**Table 1:** Summary of significant findings from studies using coherence metrics to assess connectivity.

1. Mild cognitive impairment (MCI) v. Healthy control (HC)						
Authors (Year)	Connectivity approach	Analysis basis^†^	Recording context^†^	Significant bands^†^	Group effects^	Ref.
Babiloni et al. (2009)	MSQ	Sensors - pairs	Rest - EC	Delta	MCI > HC	4
Babiloni et al. (2018b)	LL	Source - eLORETA	Rest - EC	Alpha2, alpha3	MCI < HC	7
Babiloni et al. (2019)	LL	Source - eLORETA	Rest - EC	Alpha2, alpha3	MCI < HC	8
Barzegaran et al. (2016)	LL	Source - LAURA	Rest‡ − EC, EO	Beta	MCI < HC	10
Fide et al. (2023)	IM	Sensors - pairs	Task - VO	Theta	MCI > HC	31
Handayani et al. (2018)	MSQ	Sensors - pairs	Rest - EC	Delta, theta (temporal)	MCI > HC	43
				Theta (FC), alpha, beta	MCI < HC	
Jiang (2005)	MSQ	Sensors - pairs	Task - WM	Delta, theta, alpha1, alpha2, beta1, beta2	MCI > HC	53
Jiang and Zheng (2006)	MSQ	Sensors - pairs	Task - WM	Delta, theta, alpha1, alpha2, beta1, beta2	MCI > HC	54
Jiang et al. (2008)	MSQ	Sensors - pairs	Task - WM	Delta, theta, alpha1, alpha2, beta1, beta2	MCI > HC	55
Michels et al. (2017)	MSQ, RPD	Source - Beamformer	Rest - EC	Alpha, beta	MCI < HC	72
Musaeus et al. (2019a)	MSQ, IM	Sensors - pairs	Rest - EC	MSQ: Alpha^, beta (frontal, frontal-occipital) IM: Delta	MCI < HC	76
				MSQ: Delta^, theta, beta (temporal, frontal-temporal) IM: Theta, alpha	MCI > HC	
Musaeus et al. (2019b)	MSQ	Sensors - pairs	Rest - EC, EO	Delta, beta^	MCI < HC	77
				Theta	MCI > HC	
Rodinskaia et al. (2022)	NA	Sensors - pairs	Rest - EC and task (combined)	Beta (parietal)	MCI < HC	84
				Alpha, Beta (temporal)	MCI > HC	
Tao and Tian (2006)	NA	Sensors - pairs	Task - COUNT	Gamma	MCI < HC	99
Teipel et al. (2009)	NA	Sensors - pairs	Rest - EC	Alpha	MCI < HC	100
Vanneste et al. (2021)	LL	Source - eLORETA	Rest - EC	Theta	MCI < HC	104
Xu et al. (2014)	MSQ	Sensors - pairs	Rest - EC	Alpha2	MCI < HC	117
Zheng et al. (2007)	MSQ	Sensors - pairs	Task - WM	Alpha1, alpha2	MCI > HC	124

2. Alzheimer’s disease (AD) v. Healthy control (HC)						
Authors (Year)	Connectivity approach	Analysis basis^†^	Recording context^†^	Significant bands^†^	Group effects^	Ref.
Al-Nuaimi et al. (2021)	MSQ	Sensors - pairs	Rest - NA	Alpha/theta, beta/theta ratio	AD < HC	2
Babiloni et al. (2009)	MSQ	Sensors - pairs	Rest - EC	Delta	AD > HC	4
				Alpha1	AD < HC	
Babiloni et al. (2016b)	LL	Source - eLORETA	Rest - EC	Alpha1, alpha2	AD < HC	5
				Delta, theta	AD > HC	
Babiloni et al. (2018a)	LL	Source - eLORETA	Rest - EC	Alpha2, alpha3	AD < HC	6
				Delta	AD > HC	
Barzegaran et al. (2016)	LL	Source - LAURA	Rest - EC, EO	Beta	AD < HC	10
Blinowska et al. (2017)	ORD	Sensors - pairs	Rest - EC	Theta, alpha, beta, gamma	AD < HC	12
Chan et al. (2013)	MSQ	Sensors - pairs	Rest - PS	Alpha, beta, theta	AD < HC	20
Dubovik et al. (2013)	IM	Source - Beamformer	Rest - EC	Alpha	AD < HC	27
				Theta	AD > HC	
Fide et al. (2022)	IM	Sensors - avg. regions	Rest - EC	Alpha1	AD < HC	30
				Delta	AD > HC	
Fide et al. (2023)	IM	Sensors - pairs	Task - VO	Delta, theta, alpha	AD < HC	31
Güntekin et al. (2008)	MSQ	Sensors - pairs	Task - VO	Delta, theta, alpha	AD < HC	39
Hidasi et al. (2007)	NA	Sensors - pairs	Rest - EC, EO	Alpha1	AD < HC	46
				Alpha2, beta2	AD > HC	
Ho et al. (2014)	MSQ	Sensors - CP3-F4	Task - AO	Theta	AD < HC	47
Jelic et al. (1997)	MSQ	Sensors - avg. regions	Rest - EC	Alpha	AD < HC	51
Leuchter et al. (1994)	MSQ	Sensors - pairs	Rest - EC	16 Hz	AD < HC	65
				4 Hz	AD > HC	
Locatelli et al. (1998)	MSQ	Sensors - pairs	Rest - EC	Alpha	AD < HC	69
Musaeus et al. (2019a)	MSQ, IM	Sensors - pairs	Rest - EC	MSQ: Alpha^, beta (frontal-occipital) IM: Delta	AD < HC	76
				MSQ: Theta, beta (temporal, frontal-temporal) IM: Theta, alpha	AD > HC	
Musaeus et al. (2019b)	MSQ	Sensors - pairs	Rest - EC, EO	Alpha, beta^	AD < HC	77
				Delta, theta	AD > HC	
Rodinskaia et al. (2022)	NA	Sensors - pairs	Rest - EC, Task-multiple	Rest: Beta	AD < HC	84
Sankari et al. (2011)	MSQ	Sensors - pairs	Rest - EC	Delta^, theta, alpha, beta	AD < HC	88
Sankari et al. (2012)	WF	Sensors - pairs	Rest - EC	Delta, theta, alpha, beta	AD < HC	89
Sedghizadeh et al. (2020)	IM	Sensors - pairs	Task - OO	Beta, gamma	AD > HC**§**	90
Sedghizadeh et al. (2022)	Amplitude		Task-OO	Gamma (Fz-Cz)	AD < HC	91
Tao and Tian (2006)	NA	Sensors - pairs	Rest - EC, Task - COUNT	Gamma	AD < HC	99
Vyšata et al. (2015)	Wavelet	Sensors - pairs	Rest - EC	Frontal (all freq); frontotemporal, temporal (higher freq)	AD < HC	111
				Frontoparietal (all freq); fronto-occipital (lower freq)	AD > HC	
Wang et al. (2014)	MSQ	Sensors - pairs	Rest - EC	Delta, theta, alpha1, alpha2, beta, gamma	AD < HC	112
Wang et al. (2015)	MSQ	Sensors - pairs	Rest - EC	Alpha2	AD < HC	113

Within-group effects are not shown. ^some mixed-directionality effects were found within-study, which are categorized by the most consistent pattern; †see Supplementary Table S1 for study specifications and technical descriptions; *individual landmarks; ‡ passive visual task; § Greater values on this metric represent lower connectivity; AO = auditory oddball; avg = averaged; COUNT = counting; EC = eyes closed; EO = eyes open; FC = frontal-central; IM = imaginary; LL = Lagged linear; MSQ = Magnitude squared; NA = not available/specified; OO = olfactory oddball; ORD = Ordinary; PS = photic stimulation; Ref = reference number; RPD = Renormalized partial Directed; VO = visual oddball; WF = Wavelet fraction; WM = working memory.

**Table 2:** Summary of significant findings from studies using phase-locked methods to assess connectivity.

1. Mild cognitive impairment (MCI) v. Healthy control (HC)						
Authors (Year)	Connectivity approach	Analysis basis^†^	Recording context^†^	Significant bands^†^	Group effects	Ref.
Cantero et al. (2009a)	PSI	Source - swLORETA	Rest: EC	Alpha1	MCI > HC	16
Gómez et al. (2018)	PSLI	Sensors - pairs	Rest: EC	Delta, theta, alpha, gamma	MCI < HC	36
Gonzalez-Escamilla et al. (2015)	PLI	Sensors - avg. regions	Rest: EC	Alpha	MCI < HC	37
Gonzalez-Escamilla et al. (2015)	PLI	Sensors - pairs	Rest: EC	Alpha	MCI < HC	38
Gurja et al. (2022)	LPS	Source - eLORETA	Rest: EC	Alpha1	MCI < HC	41
Handayani et al. (2018)	PLV	Sensors - pairs	Rest: EC	Alpha, beta	MCI < HC	43
Li et al. (2021)	PSI	Sensors - NA	Rest: EC	Theta	MCI < HC	67
Pons et al. (2010)	PLI	Sensors - pairs	Rest: EC	Alpha1, alpha2	MCI > HC	82
Požar et al. (2020)	PLI	Sensors - avg. regions	Rest: EC	Delta	MCI < HC	83
Ruiz-Gómez et al. (2019b)	PLI	Sensors - pairs	Rest: EC	Theta	MCI > HC	86
Spyrou et al. (2018)	PLI (tensor factorization)	Sensors - pairs	Task: memory	Theta, alpha, beta	MCI < HC	94
				Cross-component synchronization	MCI > HC	
Su et al. (2021)	PLV	Sensors - pairs	Rest: EC	Alpha	MCI < HC	95
Sweeney-Reed et al. (2012)	PLV (EMDPL)	Sensors - avg. regions	Task: memory	Theta	MCI < HC	96
Tóth et al. (2014)	PLI	Sensors - avg. regions	Rest: EO	Delta	MCI < HC	102
				Alpha1	MCI > HC	
Yan et al. (2021)	WPLI	Sensors - pairs	Rest: EC	Delta	MCI < HC	118
Youssef et al. (2021)	Debiased WPLI	Sensors - avg. regions	Rest: EC	Theta	MCI < HC	119
Zhang et al., 2022	PS	Source - DICOS	Rest: EC	Alpha	MCI < HC	122

2. Alzheimer’s disease (AD) v. Healthy control (HC)						
Authors (Year)	Connectivity approach	Analysis basis^†^	Recording context^†^	Significant bands^†^	Group effects	Ref.
Cai et al. (2018)	PSI	Sensors - pairs	Rest: EC	Delta	AD > HC	14
				Alpha	AD < HC	
Canuet et al. (2012)	LPS	Source - eLORETA	Rest: EC	Alpha 2	AD < HC	18
				Theta	AD > HC	
Engels et al. (2015)	PLI	Sensors - avg. regions	Rest: EC	Alpha1	AD < HC	28
Frangopoulou and Alimardani (2022)	PLV	Sensors - pairs	Rest: EO	Theta; Homotopic pairs: theta, delta	AD > HC	34
Gurja et al. (2022)	LPS	Source - eLORETA	Rest: EC	Alpha1	AD < HC	41
Han et al. (2017)	PLI	Sensors - avg. regions	Task: memory	Delta, theta, alpha, beta, gamma	AD > HC	42
Hata et al. (2016)	LPS	Source - eLORETA	Rest: EC, EO	Delta, theta	AD < HC	44
Kabbara et al. (2018)	PLV	Source - WMNE	Rest: EC	Theta, alpha2 (default mode network), beta (visual network)	AD < HC	57
				Theta (salience attention network)	AD > HC	
Knyazeva et al. (2010)	PSI	Sensors - NA	Rest: EC	Left frontal-temporal: delta, theta, alpha1, alpha2, beta1, beta2	AD < HC	59
				Parietal: delta, theta, alpha1, alpha2, beta1, beta2	AD > HC	
Li et al. (2019)	WPLI	Source - DBTN	Task: WM	Alpha1, alpha2, beta	AD < HC	66
				Beta (temporal regions)	AD > HC	
Mehraram et al. (2020)	WPLI	Sensors - avg. regions	Rest: EC	Alpha, beta	AD < HC	71
Ruiz-Gómez et al. (2019b)	PLI	Sensors - pairs	Rest: EC	Alpha, beta2	AD < HC	86
				Theta	AD > HC	
Wang et al. (2022)	LPS	Source - eLORETA, sLORETA	Rest: EC	Gamma	AD < HC	114
Yan et al. (2021)	WPLI	Sensors - pairs	Rest: EC	Delta	AD < HC	118
Yu et al. (2021)	PSI	Sensors - pairs	Rest: EC, EO	Broadband	AD < HC	121

Within-group effects are not shown. †see Supplementary Table S1 for study specifications and technical descriptions; avg = averaged; DBTN = Dynamic brain transition network; DICOS = Dynamic imaging of coherence sources; EC = eyes closed; EMDPL = empirical mode decomposition phase locking; EO = eyes open; LPS = Lagged phase synchronization; NA = not available/specified; PLI = phase lag index; PLV = phase locking value; PSLI = phase slope index; PSI = phase synchronization index; Ref = reference number; WPLI = weighted phase lag index; WMNE = Weighted minimum norm estimate; WM = working memory.

**Table 3:** Summary of significant findings from studies using graph theory methods to assess connectivity.

1. Mild cognitive impairment (MCI) v. Healthy control (HC)							
Authors (Year)	Connectivity approach	Analysis basis^†^	Recording context^†^	Significant metric	Significant bands^†^	Group effects	Ref.
Choi et al. (2021)	PLV	Source - WNME	Rest - EC	Clustering coefficient	Alpha1	MCI < HC	21
					Beta2	MCI > HC	
Das and Puthankattil (2020)	WPLI	Sensors - avg. regions	Rest - EC, EO; Task - Mental arithmetic	Eccentricity	Gamma (task)	MCI < HC	23
				Leaf Fraction	Alpha1 (rest)	MCI < HC	
				Eccentricity, diameter	Delta, theta, alpha1, alpha2, beta (task, rest)	MCI > HC	
				Betweenness centrality	Alpha1, alpha2 (task)	MCI > HC	
Dattola et al. (2021)	LLC	Source - eLORETA	Rest - EC	Network robustness	Broadband (1–40 Hz)	MCI < HC	24
Duan et al. (2020)	Coherence, PCOR	Sensors -NA	Rest - EC	Clustering coefficient, node strength	Theta, alpha1	MCI < HC	26
				Resilience	Theta	MCI < HC	
				Versatility	Alpha2	MCI < HC	
				Path length	Theta, alpha1	MCI > HC**§**	
				Betweenness centrality	Theta	MCI > HC	
Franciotti et al. (2019)	Granger causality	Sensors - pairs	Rest - EC	Degree, in-degree, out-degree, local efficiency, global efficiency	NA	MCI < HC	32
Frantzidis et al. (2014)	Relative wavelet entropy	Sensors - pairs	Rest - EC	Small worldness, clustering coefficient, nodal strength & significance ratio (betweenness centrality)	Broadband (>1 Hz)	MCI < HC	35
Ioulietta et al. (2020)	PCOR	Sensors-NA	Rest - EC, EO	Clustering coefficient, strength	Broadband (0.3-75 Hz)	MCI < HC	48
Josefsson et al. (2019)	JDE	Sensors-NA	Task - memory	Clustering coefficient, small worldness	Beta	MCI < HC	56
				Eccentricity	Beta	MCI > HC	
La Foresta et al. (2019)	LLC	Source - eLORETA	Rest - EC	Path length	1–40 Hz (Broadband)	MCI > HC**§**	62
Lazarou et al. (2022)	PCOR	Sensors-NA	Task - visual attention, memory	Clustering coefficient, strength	Broadband (0.3-70 Hz)	MCI < HC	63
Li et al. (2021)	PSI, DTF	Sensors-NA	Rest - EC	Clustering coefficient, node degree, global efficiency	Theta	MCI < HC	67
Mammone et al. (2018)	PDI	Sensors - pairs	Rest - EC	Path length	Broadband (1–40 Hz)	MCI > HC**§**	70
				Clustering coefficient, global efficiency	Broadband (1–40 Hz)	MCI < HC	
Miraglia et al. (2016)	LLC	Source - eLORETA	Rest - EC, EO	Small worldness	Delta, theta	MCI < HC	73
Miraglia et al. (2023)	LLC	Source - eLORETA	Hypervent	Global efficiency	Alpha1, alpha2	MCI < HC	74
Požar et al. (2020)	PLI	Sensors - avg. regions	Rest - EC	Vertex degree, degree divergence, leaf fraction	Delta	MCI < HC	83
				Vertex eccentricity, diameter	Delta	MCI > HC	
Vecchio et al. (2014)	LLC	Source - sLORETA, eLORETA	Rest - EC	Clustering coefficient	Alpha1	MCI > HC	105
Vecchio et al. (2016)	LLC	Source - sLORETA, eLORETA	Rest - EC	Small worldness	Delta	MCI < HC	106
Vecchio et al. (2021)	LLC	Source - eLORETA	Rest - EC	Small worldness	Delta, Theta	MCI < HC	109
Wei et al. (2015)	PSI	Sensors - pairs	Task - visual attention	Clustering coefficient	Alpha, beta	MCI < HC	115
	PSI	Sensors - pairs	Task - visual attention	Path length	Alpha, beta	MCI < HC**§**	
	PSI	Sensors - pairs	Task - visual attention	Small worldness	Alpha	MCI < HC	
Xu et al. (2014)	MSC	Sensors-NA	Rest - EC	Clustering coefficient	Theta, alpha1, alpha2	MCI < HC	117
				Shortest path length	Theta, alpha1, alpha2	MCI > HC**§**	
Youssef et al. (2021)	Debiased WPLI	Sensors-NA	Rest - EC (pre-, post-task)	Clustering coefficient, global efficiency	Theta (pre-task)	MCI > HC	119

2. Alzheimer’s disease (AD) v. Healthy control (HC)							
Authors (Year)	Connectivity approach	Analysis basis^†^	Recording context^†^	Significant metric	Significant bands^†^	Group effects	Ref.
Afshari and Jalili (2016)	DTF	Sensors-NA	Rest - EC	Global efficiency, attack tolerance	Alpha, beta	AD < HC	1
				Local efficiency	Alpha, beta	AD > HC	
Bagattini et al. (2022)	PDC	Sensors - avg. regions	Task - enumeration	Divisibility	Theta	AD > HC	9
Cai et al. (2018)	PSI	Sensors – pairs, global	Rest - EC	Cross-frequency, regional synchronization strength, local efficiency, small worldness	Delta-theta, delta-alpha, delta-beta	AD < HC	14
				Global efficiency	Delta-alpha, delta-beta	AD > HC	
				Local efficiency	Theta-alpha	AD < HC	
Cai et al. (2020)	NIPLV	Sensors-NA	Rest - EC	Clustering coefficient	Cross-frequency, beta	AD < HC	15
				Participation coefficient	Cross-frequency, delta (parietal)	AD < HC	
				Participation coefficient	Cross-frequency (frontal), delta (frontal-central), alpha	AD > HC	
				Node degree proportion	Delta (posterior)	AD < HC	
				Node degree proportion	Delta (frontal)	AD > HC	
Cecchetti et al. (2021)	LLC	Source - eLORETA	Rest - EC	Path length	Theta	AD < HC**§**	19
				Nodal strength, clustering coefficient	Alpha2	AD < HC	
				Nodal strength, local efficiency, clustering coefficient	Theta	AD > HC	
Choi et al. (2021)	PLV	Source - WNME	Rest - EC	Clustering coefficient	Alpha1	AD < HC	21
					Theta	AD > HC	
Dattola et al. (2021)	LLC	Source - eLORETA	Rest - EC	Network robustness	Broadband (1–40 Hz)	AD < HC	24
Duan et al. (2020)	Coherence, PCOR	Sensors-NA	Rest - EC	Clustering coefficient, node strength	Alpha1	AD < HC	26
				Resilience	Alpha1, alpha2	AD < HC	
				Betweenness centrality, versatility	Alpha1, alpha2	AD > HC	
				Path length	Alpha1, alpha2	AD > HC**§**	
Engels et al. (2015)	PLI	Sensors - avg. regions		Betweenness centrality	Beta (posterior)	AD < HC	28
				Betweenness centrality	Alpha1, alpha2, beta (anterior)^	AD > HC	
Escudero et al. (2016)	IPC	Sensors-NA	Rest - EC	Degree centrality, efficiency	Broadband (0.5-40 Hz)	AD < HC	29
Franciotti et al. (2019)	Granger causality	Sensors - pairs	Rest - EC	Degree, in-degree, out-degree, local efficiency, global efficiency, out-degree assortativity	NA	AD < HC	32
Franciotti et al. (2022)	MI	Sensors-NA	Rest - EC	Network assortativity	Broadband (1-100 Hz)	AD < HC	
Frantzidis et al. (2014)	Relative wavelet entropy	Sensors - pairs	Rest - EC	Small worldness, clustering coefficient, nodal strength (betweenness centrality)	Broadband (>1 Hz)	AD < HC	35
Ioulietta et al. (2020)	PCOR	Sensors-NA	Rest - EC, EO	Clustering coefficient, strength	Broadband (0.3-75 Hz)	AD < HC	48
Jalili (2016)	Coherence, PCOR, PO, SL	Sensors-NA	Rest - EC, EO	Global & local efficiency, betweenness centrality	Alpha	AD < HC	49
				Assortativity	Alpha	AD > HC	
Jalili (2017)	PCOR	Sensors - pairs	Rest - EC, EO	Local efficiency, modularity (EC)	Delta, theta, alpha, beta, gamma	AD < HC	50
Kabbara et al. (2018)	PLV	Source - WNME	Rest - EC	Global efficiency	Theta	AD < HC	57
				Integration	Theta, alpha1, alpha2	AD < HC	
				Connector hubs & node vulnerability	Broadband (0.1–45 Hz)	AD < HC	
				Clustering coefficient	Theta	AD > HC	
				Segregation	Theta, alpha1, alpha2	AD > HC	
				Provincial hubs	0.1–45 Hz (Broadband)	AD > HC	
La Foresta et al. (2019)	LLC	Source - eLORETA	Rest - EC	Path length	Broadband (1–40 Hz)	AD > HC**§**	62
				Clustering coefficient		AD < HC	
Lazarou et al. (2022)	PCOR	Sensors-NA	Task - visual attention, memory	Clustering coefficient, strength, betweenness centrality	Broadband (0.3–70 Hz)	AD < HC	63
Li et al. (2019)	WPLI	Source - DBTN	Task - WM	Degree, clustering coefficient	Alpha1, alpha2, beta	AD < HC	66
				Centrality index	Alpha1	AD < HC	
				Degree, clustering coefficient	Alpha1, alpha2, beta, Superior temporal - all bands	AD > HC	
				Centrality index	Alpha2	AD > HC	
Mammone et al. (2018)	PDI	Sensors - pairs	Rest - EC	Path length	Broadband (1–40 Hz)	AD > HC**§**	70
				Clustering coefficient, global efficiency	Broadband (1–40 Hz)	AD < HC	
Mehraram et al. (2020)	WPLI	Sensors - avg. regions	Rest - EC	Clustering coefficient	Alpha	AD < HC	71
Miraglia et al. (2016)	LLC	Source - eLORETA	Rest - EC, EO	Small worldness	Delta, theta	AD < HC	73
Peraza et al. (2018)	PLI	Sensors-NA	Rest - EC	Nodal degree, leaf ratio	Alpha	AD < HC	81
Smith et al. (2016)	WPLI	Sensors-NA	Rest - EC	Clustering coefficient	Beta	AD < HC	92
Tait et al. (2019)	PLF	Source - eLORETA	Rest - EO	Small worldness, closeness centrality	Theta	AD < HC	98
				Mean degree, path length	Theta	AD > HC**§**	
Vecchio et al. (2014)	LLC	Source - sLORETA, eLORETA	Rest - EC	Path length	Theta	AD > HC**§**	105
				Clustering coefficient	Theta, alpha1	AD > HC	
Vecchio et al. (2016)	LLC	Source - sLORETA, eLORETA	Rest - EC	Small worldness	Delta, theta, beta1, beta2	AD < HC	106
					Alpha	AD > HC	
Vecchio et al. (2017)	LLC	Source - sLORETA, eLORETA	Rest - EC	Small worldness	Delta, theta, beta	AD < HC	107
					Alpha	AD > HC	
Vecchio et al. (2018)	LLC	Source - sLORETA, eLORETA	Rest - EC (pre-, post-task)	Small worldness	Alpha2	AD ↓ HC ↑	108
Vecchio et al. (2021)	LLC	Source - eLORETA	Rest - EC	Small worldness	Delta, theta, beta1, beta2	AD < HC	109
					Alpha1, alpha2	AD > HC	
Vecchio et al. (2022)	LLC	Source - eLORETA	Rest - EC	Small worldness	Delta, theta	AD < HC	110
					Alpha2	AD > HC	
Wang et al. (2014)	MSC	Sensors-NA	Rest - EC	Clustering coefficient	Theta, alpha1, alpha2, beta, gamma	AD < HC	112
				Global, local efficiency, small worldness	Delta, theta, alpha1, alpha2, beta, gamma	AD < HC	
				Mean network connectivity	Theta, alpha2, gamma	AD < HC	
				Path length	Delta, theta, alpha1, alpha2, beta, gamma	AD > HC**§**	
Yu et al. (2018)	PDI	Sensors - pairs	Rest - EC	Global efficiency, clustering coefficient, small worldness	Broadband (0.5–30 Hz)	AD < HC	120
Yu et al. (2021)	Clustering coefficient; Global, local efficiency; nodal, edge between-ness	Sensors - pairs	Rest - EC, EO	Clustering coefficient, global efficiency, local efficiency	Broadband (0.5–30 Hz)	AD < HC	121
				Nodal betweenness, edge betweenness		AD > HC	

Within-group effects are not shown. †see Supplementary Table S1 for study specifications and technical descriptions; § Greater values on this metric represent lower connectivity; avg = averaged; DTF = directed transfer function; EC = eyes closed; EO = eyes open; Hypervent = hyperventilation; IPC = Imaginary part of coherence; JDE = joint distribution entropy; LLC = lagged linear coherence; LPS = Lagged phase synchronization; MSC = magnitude squared coherence; MI = Mutual information; NA = not available/specified; NIPLV = Normalized imaginary phase locking value; PCOR = Pearson correlations; PDC = partial directed coherence; PDI = Permutation disalignment index; PO = Phase order; PLF = phase locking factor; PLI = phase lag index; PLV = phase locking value; PSLI = phase slope index; PSI = phase synchronization index; Ref = reference number; SL = Synchronization likelihood; WM = working memory; WPLI = weighted phase lag index; WMNE = Weighted minimum norm estimate.

**Table 4:** Summary of significant findings from studies using miscellaneous methods to assess connectivity.

1. Mild cognitive impairment (MCI) v. Healthy control (HC)						
Authors (Year)	Connectivity approach	Analysis basis^†^	Recording context^†^	Significant bands, network^†^	Group effects	Ref.
Babiloni et al. (2009)	DTF	Sensors - avg. regions	Rest - EC	Theta, alpha1, alpha2, beta1	MCI < HC	3
Bonanni et al. (2021)	MI	Sensors – pairs, avg. regions	Rest - EC	1–100 Hz	MCI > HC	13
Cantero et al. (2009b)	DTF	Source - swLORETA	Rest - EC	Alpha1	MCI > HC	17
				Alpha2	MCI < HC	
Crook-Rumsey et al. (2023)	SNN	Sensors - avg. regions	Task - memory, WM	SNN connectivity, # of significant connections	MCI < HC	22
Guo et al. (2021)	PEC	Source - custom scripts	Rest - EC	Beta	MCI < HC	40
				Delta, theta	MCI > HC	
Koenig et al. (2005)	GFS	Sensors - global	Rest - EC	Beta	MCI < HC	61
Li et al. (2021)	DTF	Sensors - NA	Rest - EC	Theta global DTF, node degree, global efficiency	MCI < HC	67
Liu et al. (2012)	CMI	Sensors - pairs	Task - AO	Theta	NA	68
Movahed and Rezaeian (2022)	SL	Sensors - pairs	Rest - EC	Frontal-central, within-frontal, frontal-temporal, central-occipital, within-central	MCI < HC	75
				Only P3-F7, P4-Cz	MCI > HC	
Núñez et al. (2021)	Meta-states via IACDRP	Source - sLORETA	Rest - EC	Alpha dwell time; alpha & beta1 modularity	MCI < HC	79
Sedghizadeh et al. (2022)	Phase-amplitude coupling	Sensors - Fz, Cz, Pz	OO	Theta-gamma, at all three sites	MCI > HC	91
Timothy et al. (2017)	Recurrence rate from CRQA	Sensors - avg. regions	Rest - EC	Widespread (Task > Rest)	MCI > HC	101
Vanneste et al. (2021)	PACFC	Source - eLORETA	Rest - EC	Theta-gamma coupling	MCI < HC	104
Wen et al. (2014)	GSI, GCI, SES	Sensors - global	Rest - EC	GSI alpha	MCI < HC	116
				GCI alpha, beta1, beta2	MCI < HC	
				SES alpha	MCI < HC	
						

2. Alzheimer’s disease (AD) v. Healthy control (HC)						
Authors (Year)	Connectivity approach	Analysis basis^†^	Recording context^†^	Significant bands, network^†^	Group effects	Ref.
Babiloni et al. (2009)	DTF	Sensors - avg. regions	Rest - EC	Theta, alpha1, alpha2, beta1, beta2	AD < HC	3
Birba et al. (2022)	WSMI	Sensors – pairs, clusters	Rest (NA)	4-10 Hz	AD > HC	11
Blinowska et al. (2017)	DTF	Sensors - pairs	Rest - EC	Theta, alpha	AD < HC	12
Chan et al. (2013)	CMI	Sensors - pairs	Rest - EC, EO, PS	0.5-70 Hz	AD < HC	20
Herzog et al. (2022)	DTC	Source - sLORETA	Rest - EC	^Delta, theta, alpha, beta, gamma	AD < HC	45
Jeong et al. (2001)	CMI	Sensors - pairs	Rest - EC	1–35 Hz	AD < HC	52
Knyazeva et al. (2013)	SES	Source - LAURA	Rest - EC	Temporal, frontal	AD < HC	60
				Posterior	AD > HC	
Koenig et al. (2005)	GFS	Sensors - global	Rest - EC	Delta	AD > HC	61
				Alpha, Beta	AD < HC	
Lee et al. (2010)	GSI	Sensors - global	Rest - EC	Beta1, beta2, beta3, gamma	AD < HC	64
Núñez et al. (2021)	Meta-states via IACDRP	Source - sLORETA	Rest - EC	Alpha dwell time; alpha, beta1 modularity	AD < HC	79
Park and Reuter-Lorenz (2009)	GFS	Sensors - global	Rest - EC	Beta1, beta2, beta3, broadband	AD < HC	80
Ruiz-Gómez et al. (2019a)	AEC	Sensors - pairs	Rest - EC	Alpha, beta1	AD < HC	85
				Delta	AD > HC	
Ruiz-Gómez et al. (2021)	CC	Source - sLORETA	Rest - EC	Multiplex clustering coefficient	AD < HC	87
				Multiplex global strength; path length	AD > HC	
Song et al. (2018)	GCMEV	Sensors - NA	Rest - EC	0.5–40 Hz	AD < HC	93
Tahaei et al. (2012)	Synchronization via eigenratio	Sensors - NA	Rest - EC	Delta, alpha, beta, gamma	AD < HC	97
Tyrer et al. (2020)	DCM	Source - multiple spare priors	Task - memory	2–30 Hz	AD < HC	103
Vyšata et al. (2015)	MI	Sensors - pairs	Rest - EC	Frontolateral	AD < HC	111
				Centroparietal	AD > HC	
Yu et al. (2018)	PDI	Sensors - pairs	Rest - EC	PDI	AD > HC**§**	120
Zhao et al. (2019)	ROLS, DRC, AMM	Sensors - pairs	Rest - EC, EO	<70 yrs.: Nonlinear AMM	AD < HC	123
				>70 yrs.: Linear AMM, nonlinear AMM; nonlinear DRC; # of significant connections	AD > HC	

Notes: Group effects based on mean differences. Within-group effects are not shown. †see Supplementary Table S1 for study specifications and technical descriptions; ^some mixed-directionality effects were found within-study, which are categorized by the most consistent pattern; # = number; § Greater values on this metric represent lower connectivity; DBTN = Dynamic brain transition network; avg = averaged; AEC = amplitude envelope correlation; AMM = average mean magnitude; AO = auditory oddball; CC = canonical correlation; CMI = Cross-mutual information; CPSD = cross-power spectral density; CRQA = cross recurrence quantification analysis; DCM = dynamic causal modeling; DRC = dynamic range of connectivity; DTC = dual total correlation; DTF = directed transfer function; EC = eyes closed; EO = eyes open; GCMEV = generalized composite multiscale entropy vector; GCI = global clustering index; GFS = global field synchronization; GSI = global synchronization index; IACDRP = amplitude correlation-derived recurrence plots; MI = mutual information; NA = not available/specified; PACFC = phase-amplitude cross-frequency coupling; OO = olfactory odball; PDI = Permutation disalignment index; PEC = power envelope connectivity; PS = photic stimulation; Ref = reference number; ROLS = Revised orthogonal least squares; SES = S-estimator synchronization; SL = synchronization likelihood; SNN = spiking neural network; WM = working memory; WSMI = weighted symbolic mutual information.

#### 3.3.1 Coherence

Coherence is a non-directed metric that assesses the relationship between the power spectra of two signals. Coherence metrics are primarily based on the consistency of phase differences between the two sensors or brain regions, which are sensitive to both the phase and amplitude of the signals (Bastos and Schoffelen, 2016; Cao et al., 2022; Srinivasan et al., 2007).

##### 3.3.1.1 MCI

Eighteen of 20 studies (90%) that examined MCI reported significant connectivity differences between MCI and HC using coherence metrics (Table 1). Four studies reported mixed directionality, dependent on the frequency band, region, and type of coherence (Handayani et al., 2018; Musaeus et al., 2019a; Musaeus et al., 2019b; Rodinskaia et al., 2022). Eight studies reported only lower connectivity in MCI compared to HC (44%, 8/18; Babiloni et al., 2018b, 2019; Barzegaran et al., 2016; Michels et al., 2017; Tao and Tian, 2006; Teipel et al., 2009; Vanneste et al., 2021; Xu et al., 2014), while six reported only greater connectivity in MCI (33%; Babiloni et al., 2009; Fide et al., 2023; Jiang, 2005; Jiang and Zheng, 2006; Jiang et al., 2008; Zheng et al., 2007). One study analyzed resting and task conditions together, without post-hoc comparisons separating the conditions (Rodinskaia et al., 2022). Of the remaining studies, 83% (5/6) that reported greater connectivity in MCI examined active, task-related connectivity using visual oddball (Fide et al., 2023) or working memory tasks (using the same sample - Jiang, 2005; Jiang and Zheng, 2006; Jiang et al., 2008; Zheng et al., 2007). In contrast, resting state connectivity was most frequently reduced in MCI compared to HC (91%, 10/11; including three studies with mixed directionality). Furthermore, the reports of greater connectivity in MCI most frequently included the delta and/or theta band (80%; 8/10), with alpha the next most frequent (70%; 7/10), while lower connectivity in MCI was most commonly in the alpha (7/12) and beta (6/12) bands.

Given the relative advantages of connectivity analyses in source vs. sensor space, it is notable that all five studies conducted in source space reported lower resting state connectivity in MCI compared to HC, with most effects in alpha and beta bands (Babiloni et al., 2018b; Babiloni et al., 2019; Barzegaran et al., 2016; Michels et al., 2017; Vanneste et al., 2021). Moreover, the only study to report lower connectivity in MCI in the theta band used source analysis to compute connectivity between posterior cingulate and parahippocampal cortices, finding lower connectivity both within the theta band and in theta-gamma coupling between these regions (Vanneste et al., 2021).

##### 3.3.1.2 AD

Twenty-seven of 31 studies (87%) that examined AD groups found significant connectivity differences between AD and HC using coherence approaches (Table 1). Ten studies reported mixed directionality (Babiloni et al., 2018a; Babiloni et al., 2009; Babiloni et al., 2016b; Dubovik et al., 2013; Fide et al., 2022; Hidasi et al., 2007; Leuchter et al., 1994; Musaeus et al., 2019a; Musaeus et al., 2019b; Vyšata et al., 2015). All of the remaining studies reported only lower connectivity in AD compared to HC (63%, 17/27; Al-Nuaimi et al., 2021; Barzegaran et al., 2016; Blinowska et al., 2017; Chan et al., 2013; Fide et al., 2023; Güntekin et al., 2008; Ho et al., 2014; Jelic et al., 1997; Locatelli et al., 1998; Rodinskaia et al., 2022; Sankari et al., 2011, 2012; Sedghizadeh et al., 2022; Sedghizadeh et al., 2020; Tao and Tian, 2006; Wang et al., 2014, 2015), including during resting state and in all six studies that used a cognitive task (Fide et al., 2023; Güntekin et al., 2008; Ho et al., 2014; Sedghizadeh et al., 2022; Sedghizadeh et al., 2020; Tao and Tian, 2006). Only four studies (15%) analyzed coherence in source space; all four reported lower resting connectivity in AD compared to HC, two with mixed directionality (Babiloni et al., 2018a; Babiloni et al., 2016b; Barzegaran et al., 2016; Dubovik et al., 2013). One study reported their findings in terms of wavelet scales; the relationship and comparability with traditional frequency bands was not described (Vyšata et al., 2015). They overall reported lower connectivity in AD compared to HC within the frontal lobe and at higher frequencies, but greater connectivity in AD in frontal–parietal connections and at lower frequencies. Of the remaining studies, findings of lower connectivity in AD were most frequent in the alpha band (73%; 19/26), followed by beta (42%; 11/26) and theta (35%; 9/26). All reports of greater connectivity in AD were part of studies that reported mixed directionality (Babiloni et al., 2018a; Babiloni et al., 2009; Babiloni et al., 2016b; Dubovik et al., 2013; Fide et al., 2022; Hidasi et al., 2007; Leuchter et al., 1994; Musaeus et al., 2019a; Musaeus et al., 2019b), which were dependent on frequency band, region of interest, and coherence metric. The majority of the findings of greater connectivity in AD were in the delta and/or theta band (89%; 8/9), and all were during resting state.

#### 3.3.2 Phase-locked

The phase-locked metrics reported in these studies primarily included phase lag index, phase-locking value, and phase synchronization, with variations including weighted, debiased, and lagged measures. These are non-directed frequency domain metrics that assess the phase synchrony between two signals. Unlike coherence, these measures are sensitive specifically to the phase information, rather than both phase and amplitude (Bastos and Schoffelen, 2016; Cao et al., 2022; Chiarion et al., 2023).

##### 3.3.2.1 MCI

Seventeen of 22 studies (77%) reported significant connectivity effects in MCI using phase-locked approaches (Table 2). Of these, two found mixed directionality, dependent on the frequency band or connectivity metric (Spyrou et al., 2018; Tóth et al., 2014). Twelve studies reported only lower connectivity in MCI compared to HC (71%, 12/17; Gómez et al., 2018; Gonzalez-Escamilla et al., 2015; Gonzalez-Escamilla et al., 2016; Gurja et al., 2022; Handayani et al., 2018; Li et al., 2021; Požar et al., 2020; Su et al., 2021; Sweeney-Reed et al., 2012; Yan et al., 2021; Youssef et al., 2021; Zhang et al., 2022), typically in alpha (8/12), theta (5/12), or delta bands (4/12). The remaining three studies reported only greater connectivity in MCI during rest (18%, 3/17; Cantero et al., 2009a; Pons et al., 2010; Ruiz-Gómez et al., 2019b). When including studies with mixed directionality, the five studies reporting greater connectivity primarily reflected the alpha band (60%, 3/5); all but one were during rest. The task-based study used a visual short-term memory task and reported lower connectivity in MCI in theta, alpha, and beta bands, with greater synchronization between EEG components in MCI (Spyrou et al., 2018). Notably, only three studies analyzed MCI connectivity in source space, all in the resting state, with two finding lower alpha connectivity (Gurja et al., 2022; Zhang et al., 2022) and one finding greater alpha connectivity (Cantero et al., 2009a).

##### 3.3.2.2 AD

Fifteen of 20 studies (75%) found significant connectivity effects in AD using phase-locked approaches (Table 2). Seven resting state studies found only lower connectivity in AD compared to HC (47%, 7/15; Engels et al., 2015; Gurja et al., 2022; Hata et al., 2016; Mehraram et al., 2020; Wang et al., 2022; Yan et al., 2021; Yu et al., 2019). Two studies found solely greater connectivity in AD (13%, 2/15): one used a spatial memory task (Han et al., 2017) and the other was during rest (Frangopoulou and Alimardani, 2022). All other reports of greater connectivity in AD were part of studies that reported mixed directionality, which were dependent on frequency band and region (40%, 6/15; Cai et al., 2018; Canuet et al., 2012; Kabbara et al., 2018; Knyazeva et al., 2010; Li et al., 2019; Ruiz-Gómez et al., 2019b). Only one of those studies was task-related, finding lower connectivity in AD than HC during a digit span task in alpha-1 and alpha-2 bands, as well as in beta in most regions, but greater beta band connectivity specifically within temporal regions (Li et al., 2019). Of the studies reporting lower AD connectivity, the pattern was most related to alpha (69%, 9/13) and beta (38%, 5/13) bands, while greater AD connectivity was most frequent in the theta band (75%, 6/8). Notably, 40% (6/15) of the AD studies using phase-locked approaches analyzed connectivity in source space (Canuet et al., 2012; Gurja et al., 2022; Hata et al., 2016; Kabbara et al., 2018; Li et al., 2019; Wang et al., 2022). All but one showed lower connectivity in AD than HC; the exception had mixed findings with primarily lower connectivity in AD, but greater salience network-related connectivity in the theta band (Kabbara et al., 2018).

#### 3.3.3 Graph theory

Graph theory is an approach that models the brain as a complex network composed of nodes, which represent brain regions or sensors, and edges, representing the connections between them. From this foundation, numerous metrics can be investigated that provide information regarding network function and information flow (Bassett and Sporns, 2017; Bassett et al., 2018; Bullmore and Bassett, 2011). Compared to other connectivity metrics, for which lower values indicate lower connectivity, there is more nuance to interpreting graph theory metric directionality. For example, a larger divisibility value reflects greater separation between nodes, and thus suggests less efficient, or lower, connectivity. Because interpretation is highly dependent on the specific metric, we report group patterns for metrics that had at least five supporting studies. Results for all significant metrics can be found in Table 3.

Graph theory metrics with at least five supporting studies include clustering coefficient, path length, small worldness, local efficiency, and global efficiency. Clustering coefficients provide an index of how closely connected a node is to its neighbors (Masuda et al., 2018; Miraglia et al., 2022; Rubinov and Sporns, 2010; Van Diessen et al., 2014). Path length describes the number of “steps” (i.e., edges) needed to get from one node to another; a longer path length is indicative of a less interconnected and less efficient network (Miraglia et al., 2022; Rubinov and Sporns, 2010; Thilaga et al., 2018; Van Diessen et al., 2014). Small worldness describes networks that are characterized by high local clustering coefficients and short average path length between nodes (Bassett and Bullmore, 2006; Bassett and Bullmore, 2017; Rubinov and Sporns, 2010). Networks high in small worldness have fewer long-distance connections. This topology is generally considered an efficient structure for neural network processing. Global and local efficiency describe how effectively information is transferred throughout the whole network and in local regions, respectively (Achard and Bullmore, 2007; Rubinov and Sporns, 2010).

##### 3.3.3.1 MCI

Twenty-one of 24 studies (88%) reported significant connectivity effects in MCI using graph theory metrics (Table 3). Six of these studies reported mixed directionality, largely dependent on the metric of interest, but also on frequency band. The majority of the findings indicated disrupted connectivity in MCI compared to HC.

The primary graph theory metric in MCI studies was clustering coefficient, with twelve studies reporting significant clustering coefficient results. Nine of these studies reported only smaller clustering coefficients in MCI compared to HC (75%, 9/12; Duan et al., 2020; Frantzidis et al., 2014; Ioulietta et al., 2020; Josefsson et al., 2019; Lazarou et al., 2022; Li et al., 2021; Mammone et al., 2018; Wei et al., 2015; Xu et al., 2014), including both resting state and task-based connectivity (e.g., short-term memory tasks, Josefsson et al., 2019; Lazarou et al., 2022). These patterns were most commonly reported in alpha (50%, 5/10) and broadband (40%, 4/10), followed by theta (30%, 3/10). In contrast, one study reported mixed directionality (i.e., frequency band-dependent), with smaller alpha-1 clustering coefficients, but larger beta-2 coefficients in MCI (Choi et al., 2021). In addition, two studies reported only greater clustering coefficients in MCI (17%, 2/12; Vecchio et al., 2014; Youssef et al., 2021). Taken together, studies reporting greater resting state clustering coefficients in MCI, which used coherence or weighted PLI computations, were in alpha-1 (1/3), beta-2 (1/3), and theta (1/3) bands. In addition, only two studies were conducted in source space, with both showing greater connectivity in MCI (Choi et al., 2021; Vecchio et al., 2014). The connectivity approach used to compute clustering coefficients in these studies varied, including Pearson correlations, coherence metrics, phase-locked approaches, and entropy measures.

The next most frequent metric was small worldness, with six studies reporting significant group effects. All of the studies found lower small worldness in MCI compared to HC (Frantzidis et al., 2014; Josefsson et al., 2019; Miraglia et al., 2016; Vecchio et al., 2021; Vecchio et al., 2016; Wei et al., 2015). These patterns were consistent across resting state (Frantzidis et al., 2014; Miraglia et al., 2016; Vecchio et al., 2021; Vecchio et al., 2016) and task-based studies, including short-term memory recall (Josefsson et al., 2019) and visual attention (Wei et al., 2015). Half analyzed small worldness in source space (Miraglia et al., 2016; Vecchio et al., 2021; Vecchio et al., 2016). Results included delta (3/6), theta (2/6), beta (2/6), alpha (1/6), and broadband (1/6). The most common approach used to compute small worldness was lagged linear connectivity (3/6).

Five studies reported significant effects with path length, four of which found greater path length in MCI compared to HC (Duan et al., 2020; La Foresta et al., 2019; Mammone et al., 2018; Xu et al., 2014), including the only study in source space (La Foresta et al., 2019). Half reported greater path length in theta and alpha bands (Duan et al., 2020; Xu et al., 2014) and the other half used a broadband approach (La Foresta et al., 2019; Mammone et al., 2018). The only study to report lower path length in MCI compared to HC was during a visual attention task, with significant effects in alpha and beta (Wei et al., 2015). Approaches to computing path length varied widely, including coherence metrics, correlations, and phase-locked approaches.

Five studies had significant findings with global efficiency. Four found lower global efficiency in MCI compared to HC (Franciotti et al., 2019; Li et al., 2021; Mammone et al., 2018; Miraglia et al., 2023), including the only one in source space (Miraglia et al., 2023). Results included broadband (2/4), alpha (1/4), and theta (1/4). The remaining study found greater global efficiency in MCI in the theta band (Youssef et al., 2021). Connectivity metrics used to compute efficiency included phase-locked, coherence, and correlational metrics.

##### 3.3.3.2 AD

Thirty-five of 36 studies (97%) reported significant connectivity effects in AD using graph theory metrics (Table 3). The majority (56%, 20/36) reported mixed directionality, with effects dependent on both the metric of interest and the frequency band. The majority of the findings indicated disrupted connectivity in AD compared to HC.

The most frequent metric in AD studies was clustering coefficient, with 16 studies reporting significant results. Three studies, all done at source-level, reported mixed directionality (i.e., dependent on frequency band and/or region), with two at rest (Cecchetti et al., 2021; Choi et al., 2021) and one during a digit span task (Li et al., 2019). At rest, alpha clustering coefficients were smaller, while theta coefficients were larger in AD compared to HC. During digit span, AD alpha and beta clustering coefficients were smaller in frontal and postcentral regions, but larger in the superior temporal sulcus. The other two studies performed in source space reported only larger clustering coefficients in AD compared to HC at rest (2/16, 13%; Kabbara et al., 2018; Vecchio et al., 2014), with findings in the theta band and alpha-1. In contrast, eleven studies reported only smaller clustering coefficients in AD compared to HC (69%, 11/16; Cai et al., 2020; Duan et al., 2020; Frantzidis et al., 2014; Ioulietta et al., 2020; La Foresta et al., 2019; Lazarou et al., 2022; Mammone et al., 2018; Mehraram et al., 2020; Smith et al., 2016; Wang et al., 2014; Yu et al., 2018). Taken with the three mixed directionality studies, most reports of lower clustering coefficients were in the alpha and beta bands (57%, 8/14), with alpha most frequent. The connectivity approaches used to compute clustering coefficient in these studies varied widely, most frequently using coherence, correlations, or phase-locked metrics.

The next most frequent metric in AD studies was small worldness, with eleven studies, all analyzing connectivity during resting state. Four studies reported mixed directionality (36%; Vecchio et al., 2022; Vecchio et al., 2021; Vecchio et al., 2017; Vecchio et al., 2016), dependent on the frequency band. Specifically, while comparisons across most bands showed lower small worldness in AD compared to HC (including delta, theta, and beta bands), small worldness in the alpha band was greater in each of these four studies (Vecchio et al., 2022; Vecchio et al., 2021; Vecchio et al., 2017; Vecchio et al., 2016). Indeed, most reports of greater small worldness were in the alpha band (4/5), all four of which were from the same research group, computed via lagged linear coherence. The other study showed greater small worldness in cross-frequency bands between delta with theta, alpha, and beta (Cai et al., 2018). All other studies reported only lower small worldness in AD compared to HC (Frantzidis et al., 2014; Miraglia et al., 2016; Tait et al., 2019; Wang et al., 2014; Yu et al., 2018), showing results in the theta band (78%, 7/9) and delta band (67%, 6/9). Vecchio and colleagues (Vecchio et al., 2018) further showed that alpha-2 small worldness during resting state decreased in AD but increased in HC when change was measured from before, then to during a sensory motor learning task, and then to after the task. The majority of the small worldness studies (64%, 7/11) were conducted in source space (Ferreri et al., 2016; Miraglia et al., 2016; Tait et al., 2019; Vecchio et al., 2022; Vecchio et al., 2021; Vecchio et al., 2017; Vecchio et al., 2018), and most were computed with a coherence metric (7/11).

Ten studies reported significant group effects in global efficiency. All but one (Cai et al., 2018) found lower efficiency in AD compared to HC during rest (Afshari and Jalili, 2016; Escudero et al., 2016; Franciotti et al., 2019; Jalili, 2016; Kabbara et al., 2018; Mammone et al., 2018; Wang et al., 2014; Yu et al., 2019; Yu et al., 2018). The one study to find greater efficiency in AD analyzed cross-frequency coupling, finding greater global efficiency specifically in delta-alpha and delta-beta (Cai et al., 2018). Only one study analyzed efficiency in source space (Kabbara et al., 2018). Overall, the most frequent findings were in broadband (5/10) and alpha bands (3/10). Metrics used to compute efficiency varied widely.

Eight studies reported significant group effects in local efficiency, all during resting state. Six found lower local efficiency in AD compared to HC (Cai et al., 2018; Franciotti et al., 2019; Jalili, 2016, 2017; Wang et al., 2014; Yu et al., 2019), most frequently in alpha (3/6). The two remaining studies found greater local efficiency in AD compared to HC, one in theta band in source space (Cecchetti et al., 2021) and the other in alpha and beta in sensor space (Afshari and Jalili, 2016). Metrics used to compute efficiency varied widely.

Seven studies reported significant effects in path length. All of these studies were conducted using resting state data, and all but two used coherence metrics (Mammone et al., 2018; Tait et al., 2019). Most reported greater path length in AD compared to HC (86%, 6/7; Duan et al., 2020; La Foresta et al., 2019; Mammone et al., 2018; Tait et al., 2019; Vecchio et al., 2014; Wang et al., 2014). Specifically, two studies showed the effect in the theta band (source space, Tait et al., 2019; Vecchio et al., 2014), two using broadband metrics (source space, La Foresta et al., 2019; sensor space, Mammone et al., 2018), one in alpha-1 and alpha-2 (sensor space, Duan et al., 2020), and one found significant effects in delta, theta, alpha-1 and alpha-2, beta, and gamma bands (sensor space without specific statistics reported, Wang et al., 2014). The only study to report lower path length in AD compared to HC was in the theta band using source space (Cecchetti et al., 2021).

#### 3.3.4 Other methods

Of the 124 total papers in this review, 31 reported significant effects using methods distinct from the previously described categories. Examples of these other methods include variations of mutual information (e.g., cross-mutual information, weighted symbolic mutual information), amplitude envelope correlation, and directed transfer function, amongst others, some of which are based in correlational approaches. A collective summary of results from these studies would be overly speculative. Thus, we show the methodological approach of each study in Supplementary Table S3 and the results of each individual study in Table 4. Here we summarize only a few overarching points. In MCI, 14 of 17 studies (82%) using these other methods found significant group differences. Most studies found reduced connectivity in MCI, although not all studies reported the directionality of the effect, and directionality varied across the methods, regions, and frequency bands, particularly when task-related. Of those with significant findings, four studies were conducted in source space (29%, 4/14). Three found only lower connectivity in MCI compared to HC, and the fourth had mixed directionality depending on the alpha sub-band. In AD participants, 19 of 25 studies (76%) reported significant connectivity effects. The majority of the results indicated lower connectivity in AD compared to HC, but with variability by method, region, and frequency band. Five of the studies with significant findings were conducted in source space (26%, 5/19). Three found only lower connectivity in AD compared to HC; the other two had mixed directionality, dependent on region and metric of interest. Of note, a longitudinal study of MCI participants who later progressed to AD reported greater broadband connectivity (1–100 Hz, via mutual information between sensors) only during the prodromal (i.e., MCI) stage, which was no longer evident at the time of AD diagnosis or at three years post-diagnosis (Bonanni et al., 2021). This evidence of hyperconnectivity in the prodromal stage was theorized to be indicative of plasticity (i.e., recruitment) that subsides with greater disease progression.

## 4 Discussion

It has been theorized that AD may best be characterized as a disconnection syndrome (Delbeuck et al., 2003; Stam, 2014; Yu et al., 2021). As such, recent research has evolved to emphasize studies of neural connectivity differences and changes in people diagnosed with MCI and AD relative to cognitively healthy elders. As this work has rapidly expanded, many techniques have been developed to examine connectivity using EEG (Bastos and Schoffelen, 2016; Cao et al., 2022; Chiarion et al., 2023; Srinivasan et al., 2007). The particular advantage of EEG is to capture neural connectivity in real time, with millisecond-level precision, and without use of a proxy (Luck, 2014; Slotnick, 2017). Yet, there are no truly comprehensive systematic reviews of this literature. Thus, the current study conducted such a systematic review, comparing findings across EEG connectivity methods, in resting and task-activated states, where MCI and/or AD participants were compared to cognitively healthy elders. Ultimately, 124 studies were included, with 35 that examined MCI relative to HC, 56 that examined AD relative to HC, and 33 that examined all three groups. The primary methods used to examine EEG connectivity were coherence, phase-locked, and graph theory metrics, although various other approaches were also employed. The majority of the approaches were non-directed, phase-based metrics in the frequency domain; thus, results primarily speak to the synchronicity of timing of neural population activity between different brain regions, without inferring causal direction of information flow (Cohen, 2014). With the exception of graph theory, where multivariate connectivity was more prevalent, most of the connectivity metrics were bivariate, analyzing the relationship between two pairwise signals. Here we discuss patterns that emerged within method types, and across studies, as well as the variability and limitations in the existing literature, toward enhancing future EEG connectivity research with these populations.

### 4.1 Connectivity differences in MCI/AD relative to HC

**Figure 2:**
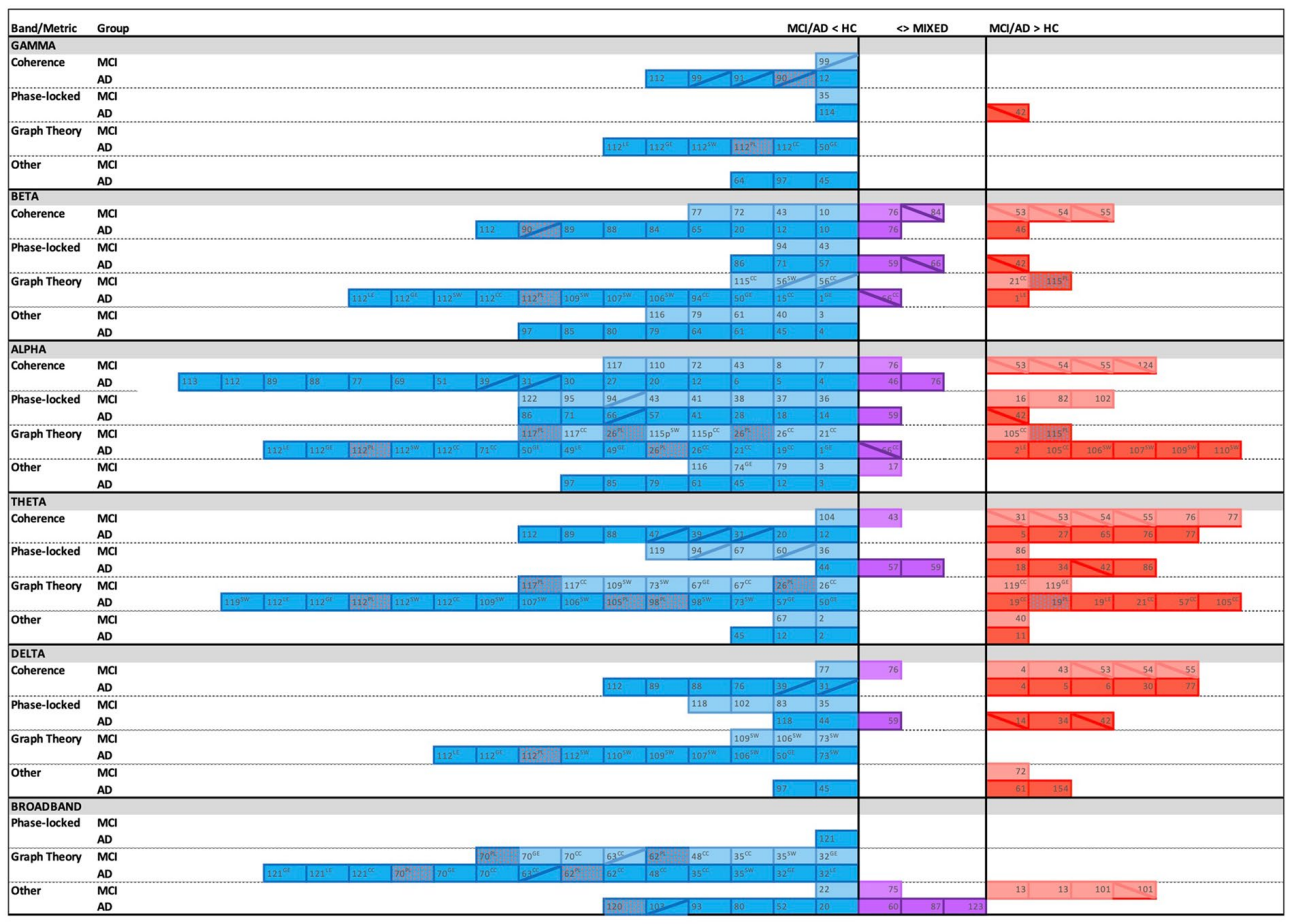
Overview of findings across connectivity metrics, frequency bands, and groups with corresponding reference numbers (see Tables 1–4), with **blue** indicating lower connectivity, **red** indicating greater connectivity, and **purple** representing mixed directionality in Alzheimer’s disease (AD) and/or Mild Cognitive Impairment (MCI) groups relative to healthy controls (HC). Twelve studies are not shown due to purely negative findings (*n* = 3), unclear directionality (*n* = 1), unique study design (*n* = 2), only cross-frequency results (*n* = 1), or graph theory methods represented by less than five studies (*n* = 5). Solid cell = EEG recorded at rest; diagonal line (╲ or ╱) = EEG recorded during task; GE = global efficiency; LE = local efficiency; PL = path length; CC = clustering coefficient; SW = small worldness; Dotted fill = greater PL implies poorer network efficiency (**blue** cell, **red** dots), or lower PL implies better network efficiency (**red** cell, **blue** dots). Overall, the most frequent reports were of lower connectivity in MCI/AD vs. HC (**blue**), especially in the alpha band. Greater connectivity in MCI/AD vs. HC (**red**) was most commonly reported in the theta band and during task-based studies in MCI.

The majority of the included studies found significant differences in EEG connectivity between cognitively healthy elders and those with MCI or AD. While there was variability in the directionality of the effects, an overarching pattern emerged of lower connectivity in both MCI and AD compared to healthy controls, with patterns most consistent in AD (see Figure 2). The trends are in line with the expected progressive neural network degradation consequent to AD (Alzheimer’s Association, 2024). Notably, lower connectivity was most robust in the alpha band, followed by beta and theta. When greater connectivity was reported in MCI or AD relative to controls, it was most common in the theta band, followed by delta and alpha. EEG research on power within specific regions (e.g., power spectral density) collectively suggests a pattern of neural slowing during AD progression, with greater power in slower bands and lower power in faster bands (Dauwels et al., 2010; Smailovic and Jelic, 2019). The connectivity findings herein are in relative agreement with those conclusions, showing prevalent patterns of lower connectivity in alpha and beta bands and greater connectivity in theta and delta bands. However, there were also frequent reports of greater connectivity in alpha and lower connectivity in theta. Although various study differences, particularly in sample characteristics (e.g., age, degree of cognitive decline, and possible resilience factors such as education) may contribute to conflicting findings, these differences could also suggest a more nuanced interpretation. Specifically, the overall prevalence of results in alpha frequencies and the prevalence of findings with both lower and greater connectivity suggests that alpha may be particularly sensitive to the transition from the dominance of faster to slower frequencies that typifies AD-related change (Hamilton et al., 2021; Wijaya et al., 2023).

While patterns of lower connectivity were evident overall in MCI and AD relative to controls, more nuanced trends emerged when considering the EEG recording context. In AD, lower connectivity was common whether measured during the resting state or during active task engagement. This finding suggests that connectivity deficits are relatively widespread across various neural networks in AD. However, in MCI, lower connectivity was most commonly reported during the resting state, while greater connectivity was more often found during task engagement. This difference of recording context-dependent patterns between MCI and AD suggests that those with MCI may still be able to engage compensatory resources, while these resources are more likely to already be exhausted in AD (Paitel and Nielson, 2023; Rao et al., 2015; Reuter-Lorenz and Cappell, 2008; Reuter-Lorenz and Park, 2014). Specifically, compensatory theories of cognitive aging suggest that during earlier stages of disease progression there is a period of increased brain activity and connectivity that reflects compensation for AD-related neuropathology, thereby allowing for the maintenance of task performance (Cabeza, 2002; Cabeza et al., 2002; Davis et al., 2008; Park and Reuter-Lorenz, 2009; Reuter-Lorenz and Cappell, 2008; Reuter-Lorenz and Park, 2014). However, such compensatory resources are finite; as neuropathology advances, these resources are exhausted, which results in the progression of cognitive impairment (Park and Reuter-Lorenz, 2009; Rao et al., 2015; Reuter-Lorenz and Cappell, 2008; Reuter-Lorenz and Park, 2014). Thus, greater connectivity specifically during task performance in MCI suggests that some compensatory resources may remain, at least early in MCI, and that these resources are recruited during task engagement. This is also consistent with a recent study of working memory encoding that showed lower directed connectivity from prefrontal to temporal lobes, but greater connectivity from prefrontal to parietal and occipital lobes in MCI compared to HC (Jiang et al., 2024).

Although neural compensation is more common early in cognitive decline, it is most notable in cognitively healthy elders at elevated risk for AD (Elverman et al., 2021; Rao et al., 2015; Reuter-Lorenz and Park, 2014; Sugarman et al., 2012). Importantly, the greatest risk factor for AD other than age is inheritance of the APOE ε4 allele (Alzheimer’s Association, 2024; Yu et al., 2014). We would therefore expect greater connectivity in asymptomatic ε4 carriers than in individuals with MCI, with evidence of reduced connectivity in carriers who eventually develop cognitive symptoms (Paitel and Nielson, 2023; Rao et al., 2015). However, attempts to evaluate this expectation failed; no studies of cognitively healthy ε4 carriers were available to include in the review. Yet, within MCI and AD samples, a few studies examined the role of ε4. Two studies saw no ε4 differences within MCI (Cantero et al., 2009a; Cantero et al., 2009b), while two others found lower alpha phase lag index connectivity (primarily frontal) in ε4+ compared to ε4- (Gonzalez-Escamilla et al., 2015; Gonzalez-Escamilla et al., 2016), which is consistent with our expectation. Of note, these two studies were from the same research group and conducted with the same sample. Only one study examined ε4 in AD, showing that lower connectivity in AD relative to HC was attributable specifically to homozygous ε4+ (i.e., carrying both ε4 alleles), thus suggesting a persistent influence of ε4 (Jelic et al., 1997). The general lack of studies considering ε4, and particularly the lack of study of asymptomatic carriers precludes drawing any clear conclusions and highlights an important gap in the existing literature.

While coherence and other phase-based metrics may be interpreted to generally quantify the interdependence of signals between brain regions, graph theory metrics provide more specific information regarding network function and information flow (Bassett and Sporns, 2017; Bassett et al., 2018; Bullmore and Bassett, 2011). Overall, the reviewed studies revealed that MCI and AD networks had lower density of interconnected notes, via clustering coefficient. Contrasting reports of larger clustering coefficients were most common specifically in the theta band in AD, which may suggest that clustering of connections in that lower frequency band remains relatively robust, compared to the faster bands (Abazid et al., 2022). Furthermore, MCI and AD groups overall had greater path length than HC groups, indicating less integrated networks (Miraglia et al., 2022; Rubinov and Sporns, 2010; Van Diessen et al., 2014). Taking into account both clustering coefficient and path length, MCI and AD networks were generally characterized by lower small worldness, which is often interpreted as the balance between network segregation and integration (Miraglia et al., 2022; Rubinov and Sporns, 2010). The exception was a collection of studies from Vecchio and colleagues that reported greater small worldness in AD specifically in the alpha band (Vecchio et al., 2022; Vecchio et al., 2021; Vecchio et al., 2017; Vecchio et al., 2016). Closer investigation of those results revealed that small worldness values in HC groups were modulated by frequency band, with relatively higher values in delta, theta, and beta bands and lower values in alpha and gamma. In contrast, small worldness in the AD group tended to be “flatter,” or more static, across frequency bands. Thus, the organization of neural networks may become less adaptive with AD progression. Finally, MCI and AD networks were overall less efficient compared to those of HC, considering both global and local network efficiency. Taken together, the findings of these graph theory metrics suggest that neural networks become less efficient in MCI and AD, compared to healthy older adults, consistent with the expected impact of AD-related neuropathology, particularly synaptic dysfunction and loss of neural connections (Delbeuck et al., 2003; Martínez-Serra et al., 2022; Shankar and Walsh, 2009).

There are many graph theory metrics, which each describe a different aspect of neural networks. Across the 44 studies that used graph theory metrics, over 30 different metrics were reported. There was such variability in which metrics were analyzed between studies that only five could be summarized in AD and four in MCI (with ≥5 studies). Yet, graph theory metrics were particularly informative, especially in AD, with 97% (i.e., 35/36) of studies finding significant group effects. Thus, graph theory may be particularly advantageous, given its ability to quantify important aspects of complex network organization that result from AD-related neuropathology. In MCI, significant effects were most consistently detected using coherence (90%, 18/20), closely followed by graph theory metrics (88%, 21/24). It is possible that network-level disruptions assessed with graph theory metrics become more robust with disease progression, with simpler, between-region connectivity assessments more robust in earlier stage decline (i.e., MCI). However, other factors may be responsible for the marginal difference in detection of group effects, and indeed, with 88% of studies finding significant patterns, graph theory is likely to provide key insights into network-level changes earlier in the AD spectrum. More research with graph theory in MCI, AD, and cognitively healthy groups with AD risk will certainly advance understanding of the timeline of network-level neural changes in the course of AD.

### 4.2 Study quality and transparency of reporting

Considering studies that reported sample demographics, there was a very wide range of sample sizes, especially with AD samples (range = 6 to 318). Particularly given the large number of comparisons made in connectivity studies, some of the studies likely did not have sufficient statistical power to assure the interpretations. Despite that concern, sample sizes were generally well balanced for the relative comparisons. There were also age differences that may have influenced study findings. HC groups were significantly younger overall than both MCI and AD groups, which would amplify MCI and AD connectivity differences by confounding them with expected age-related differences (Ferreira and Busatto, 2013; Park and Reuter-Lorenz, 2009; Reuter-Lorenz and Park, 2014; Sala-Llonch et al., 2015). Similarly, AD groups had overall lower education than both MCI and HC groups, which would serve to artificially exacerbate group differences (Montemurro et al., 2023; Roldán-Tapia et al., 2017; Tucker and Stern, 2011). While sex distributions were overall comparable between groups, we note that there are few studies interrogating sex differences in these neural patterns, which would be valuable given the greater risk of AD in women (Alzheimer’s Association, 2024; Andrew and Tierney, 2018; Mielke et al., 2014). We further note that evaluating gender differences, beyond simply sex differences, is also of great importance but is essentially unstudied (Correro and Nielson, 2020; Mielke, 2018; Mielke et al., 2014). Supplementary Table S4 includes report of which studies controlled for age, education, and sex by study design or analysis. Finally, the range of average MMSE scores raised some concerns about the validity of the diagnostic groupings. The MMSE (range = 0–30) generally uses ≤24 as a cutoff for cognitive impairment (≤ 26 has been suggested as superior; Chun et al., 2021; Folstein et al., 1983; Kvitting et al., 2019; Salis et al., 2023). However, the average HC MMSE scores were as low as 25.7, while average MCI scores were as high as 28.4, and average AD scores were as high as 26.3. These variances can suppress or obfuscate real group differences and their interpretations.

An important caveat to the interpretation of the sample characteristics in this review is the generally low rate of reporting such information (see Supplementary Table S1 and S4). Specifically, 15% of studies did not include mean ages of the groups, 17% did not report sex distribution, 33% did not report global cognitive functioning metrics, and 51% did not report educational attainment. Reporting and transparency about these factors is essential for contextualizing and interpreting patterns of neural activity in MCI and AD. Specifically, age is the greatest risk factor for AD (Alzheimer’s Association, 2024), women have a higher risk of developing AD that is not due to greater longevity (O’Neal, 2024), education is an important cognitive resilience factor (Tucker and Stern, 2011), and global cognitive functioning is needed to assure the comparability across studies and clarity of diagnostic criteria.

A meta-analysis of this literature would have been extremely valuable to expand upon our descriptive analysis. However, this was not feasible due to a lack of detailed statistical reporting in a large proportion of the papers, along with vast numbers of methods and comparisons in most studies (including sensors/ROIs, frequency bands, and groups; see Supplementary Table S3).

### 4.3 Methodological considerations for studying and interpreting connectivity

#### 4.3.1 Sensor versus source space

While many EEG research questions may be best addressed using sensor-level data (e.g., event-related potentials from cortical regions), connectivity analyses involve a higher risk of spurious connectivity when conducted at the sensor level (Chiarion et al., 2023; Mahjoory et al., 2017; Michel and Brunet, 2019; Schoffelen and Gross, 2009; Van de Steen et al., 2019). For this reason, relying solely on findings from sensor-level studies may over-estimate connectivity results. Specifically, sensor-level results may carry a greater risk of detecting patterns that may not actually be attributable to shared neural coupling or co-activation. Connectivity analyses in source space are better equipped to delineate the likely neural generators of EEG signals and more appropriately account for underlying sources of shared variance, thus increasing the likelihood of accurately modeling connectivity between two distinct brain regions. Despite these important advantages, only 29% (i.e., 36/124) of the current studies analyzed connectivity in source space. Trends from sensor- and source-level studies in the present review had overall similar patterns. That is, there was no discernible difference in preponderance or direction of effects using source space rather than sensor-level data. Yet, the subset of studies available with source-level findings was too limited to in any way be conclusive. We advocate for future analyses in source space, which has become particularly accessible and feasible with advances in user-friendly, open-source software and detailed published tutorials (e.g., Delorme and Makeig, 2004; Oostenveld et al., 2011; Penny et al., 2011; Tadel et al., 2011). This does require thoughtful consideration of the appropriate computation of sources. For example, some studies analyzed 84 ROIs computed from only 19 electrodes; a low-density EEG array is not ideal for delineating a large number of sources (Michel and Brunet, 2019; Song et al., 2015). Relatedly, thoughtful consideration should be given to using *a priori* brain regions of interest, rather than extensive or exhaustive numbers of comparisons.

#### 4.3.2 Resting versus task state

The majority of the papers reviewed here analyzed EEG connectivity during the resting state. Given the cognitive limitations inherent in studying MCI and AD, this tendency toward resting state analysis is understandable. However, the current findings are certainly biased toward documenting patterns within the default mode network. Future complementary study is needed using tasks that tap specific cognitive processes, with attention to high or comparable task accuracy across groups (at least in milder forms of cognitive impairment). High accuracy tasks discern neural activity patterns that are not conflated with group differences due to error or cognitive demand (Reuter-Lorenz and Cappell, 2008). Some examples include tasks that tap aspects of crystallized intelligence (e.g., vocabulary; Ferré et al., 2019; Salthouse, 2014), semantic access (Nielson et al., 2006; Pistono et al., 2019; Seidenberg et al., 2009; Sugarman et al., 2012), stop-signal tasks (Elverman et al., 2021; Hsieh and Lin, 2017a, 2017b; Paitel and Nielson, 2021, 2023), and oddball paradigms (Invitto et al., 2018; Iragui et al., 1993; Schiff et al., 2008; Stevens et al., 2000).

#### 4.3.3 Frequency domain

The most common approaches to analyzing connectivity used metrics that are based on EEG frequencies (e.g., coherence, phase lag index, etc.). While there are advantages to investigating connectivity within specific oscillatory bands, these approaches are limited in temporal resolution (Cohen, 2014; Luck, 2014). A primary strength of EEG is its precise recording of summated post-synaptic potentials with millisecond temporal resolution (Luck, 2014; Slotnick, 2017). However, most frequency domain approaches average activity across several seconds, thereby compromising that temporal precision. Such an approach is more appropriate for resting state analyses, in which participants are not completing a specific task. However, in the context of active task engagement, temporal precision provides crucial information regarding temporally specific neural network connectivity. In using longer time windows of several seconds to allow for higher frequency resolution, the ability to provide temporal precision about cognitive subprocesses is compromised. For example, Li et al. (2019) used a digit span task, but across epochs of 17 s, which was inclusive of two seconds pre-stimulus, the ten second duration of the stimulus presentation, and five seconds post-stimulus. Thus, rather than specifically analyzing neural activity relevant to the holding or manipulating of information in working memory, this lengthy window encompassed a broad number of cognitive processes underlying task performance.

The vast majority of the studies analyzed connectivity within a specific frequency band. Yet, oscillatory activity in the brain simultaneously occurs across multiple frequency ranges. For this reason, there has been a recent increase in prevalence of cross-frequency approaches that analyze the relationship between activity across different bands. Two categories of cross-frequency coupling are phase-amplitude and phase-phase coupling. Recent work with these methods points to their potential import for understanding the neuroscience of cognition (Abubaker et al., 2021; Canolty and Knight, 2010; Riddle et al., 2021), as well as for application in contexts such as MCI and AD (Dimitriadis et al., 2015; Musaeus et al., 2020). Of the studies in the present review that analyzed cross-frequency coupling, most found lower coupling in MCI or AD compared to HC, including theta-gamma coupling in MCI (Vanneste et al., 2021), and delta-theta, delta-alpha, delta-beta (Cai et al., 2018) and multiplex (across four bands and two bands) network features (Cai et al., 2020) in AD. In contrast, the only study during active task engagement found greater theta-gamma coupling for frequent (non-oddball) trials in MCI compared to HC during an olfactory oddball task (Sedghizadeh et al., 2022). Thus, while this area of research is young, it is likely that unique insights may be obtained by pursuing application in MCI and AD.

#### 4.3.4 Time domain

In contrast to the frequency domain, connectivity approaches in the time domain, such as those based in correlations, allow for shorter time windows, and thus superior temporal resolution. The primary limitation of such correlational methods is that most do not account for volume conduction. However, this is not a concern that is unique to correlational methods. For example, magnitude squared coherence is also sensitive to volume conduction (Bastos and Schoffelen, 2016; Khadem and Hossein-Zadeh, 2014; Ruiz-Gómez et al., 2019a; Srinivasan et al., 2007). Measures such as imaginary coherence have been specifically developed to better account for those effects (Nolte et al., 2004). Furthermore, with recent advances in computational processing, approaches that allow for evaluating temporal–spatial dynamics are becoming more feasible. For example, Crook-Rumsey et al. (2023) used a novel network modeling approach (i.e., spiking neural networks) during working memory and prospective memory tasks to interrogate connectivity within three separate time windows: 200-400 ms (cue detection and monitoring), 400-800 ms (deeper contextual and memory processes), and 0-1,000 ms (full epoch). They found lower connectivity in MCI compared to HC that was specific to the 200 to 400 ms window in a central cluster during a 1-back task, and in lateral frontal clusters during a perceptual prospective memory task. Importantly, these effects were not significant using the full 1,000 ms time window.

### 4.4 Conclusion and future directions

Altogether, the existing literature highlights EEG connectivity as a powerful tool to advance understanding of AD-related changes in brain communication via functional and effective connectivity metrics. The studies reviewed highlight overall dominant patterns of lower connectivity in MCI and AD compared to HC, particularly in the alpha band. The prevalence of findings in the alpha band may suggest that it holds particular promise for characterizing AD-related neural network changes. It is notable that the majority of the studies report on alert resting state connectivity, for which alpha is the dominant EEG frequency (Millett, 2001; Nunez and Srinivasan, 2006). Thus, different patterns or other prominent frequencies may emerge when a greater proportion of task-based studies is available. Contrasting patterns of greater connectivity were most common in the theta band for both MCI and AD, and were more prevalent in MCI during task engagement, suggestive of the recruitment of neural resources to accomplish the task (Reuter-Lorenz and Cappell, 2008; Reuter-Lorenz and Park, 2014).

This review explores connectivity patterns with attention to the influence of factors including sample characteristics, study design, and methodological considerations. Overall trends in connectivity were evident despite substantial variability in these factors across studies. The most consistent reports of significant group differences were found using graph theory metrics in AD and coherence in MCI (closely followed by graph theory). We further suggest the need to extend this work to cognitively healthy, high-AD-risk older adults to advance characterization of the earliest possible indicators of network-level changes that may portend risk for future cognitive decline.

The present review exclusively focused on sporadic, late-onset AD, which is the most common form (Alzheimer’s Association, 2024). In contrast, early-onset AD (prior to age 65) is rare but has a stronger genetic component (APP, PSEN1, or PSEN2 variants) and is associated with a more aggressive and often atypical clinical presentation (Mendez, 2017). At present, there is relatively less research on connectivity in early-onset AD; greater study and synthesis of such research may reveal distinct patterns and progression of neural network changes, both compared with late-onset AD, but also in distinguishing the distinct phenotypes of early-onset AD (Adebisi et al., 2024; Adebisi and Veluvolu, 2023; Filippi et al., 2017; Gour et al., 2014).

We note that there was a paucity of *a priori*, theory-driven analyses in specific bands, time windows, and between specific brain regions. Many studies conducted a very large number of exploratory comparisons, including bivariate comparisons of sensors and regions across the whole brain. This approach limits the meaningfulness of interpretations, elevates the likelihood of statistical error, and reduces the likelihood of replication. We advocate for future studies employing thoughtful *a priori* hypotheses guided by the existing literature, to improve the clarity and confidence of interpretations and replications.

## Data Availability

The data analyzed in this study is subject to the following licenses/restrictions: this paper is a systematic review of previously published studies. The summary data generated to conduct the review and synthesize findings across studies can be obtained by request to the corresponding author. The original datasets are not accessible through our study. Requests to access these datasets should be directed to kristy.nielson@marquette.edu.

## References

[ref1] Abazid M. Houmani N. Dorizzi B. Boudy J. Mariani J. Kinugawa K. (2022). Weighted brain network analysis on different stages of clinical cognitive decline. Bioengineering 9:62. doi: 10.3390/bioengineering9020062, PMID: 35200415 PMC8869328

[ref2] Abubaker M. Al Qasem W. Kvašňák E. (2021). Working memory and cross-frequency coupling of neuronal oscillations. Front. Psychol. 12:756661. doi: 10.3389/fpsyg.2021.756661, PMID: 34744934 PMC8566716

[ref3] Achard S. Bullmore E. (2007). Efficiency and cost of economical brain functional networks. PLoS Comput. Biol. 3:e17. doi: 10.1371/journal.pcbi.0030017, PMID: 17274684 PMC1794324

[ref4] Adebisi A. T. Lee H.-W. Veluvolu K. C. (2024). EEG-based brain functional network analysis for differential identification of dementia-related disorders and their onset. IEEE Trans. Neural Syst. Rehabil. Eng. 32, 1198–1209. doi: 10.1109/TNSRE.2024.3374651, PMID: 38451768

[ref5] Adebisi A. T. Veluvolu K. C. (2023). Brain network analysis for the discrimination of dementia disorders using electrophysiology signals: a systematic review. Front. Aging Neurosci. 15:1039496. doi: 10.3389/fnagi.2023.1039496, PMID: 36936496 PMC10020520

[ref6] Afshari S. Jalili M. (2016). Directed functional networks in Alzheimer's disease: disruption of global and local connectivity measures. IEEE J. Biomed. Health Inform. 21, 949–955. doi: 10.1109/JBHI.2016.2578954, PMID: 27305688

[ref7] Al-Nuaimi A. H. Blūma M. Al-Juboori S. S. Eke C. S. Jammeh E. Sun L. . (2021). Robust EEG based biomarkers to detect Alzheimer’s disease. Brain Sci. 11:1026. doi: 10.3390/brainsci11081026, PMID: 34439645 PMC8394244

[ref8] Alzheimer’s Association (2022). More than normal aging: understanding mild cognitive impairment. Alzheimers Dement. 18, 545–868. doi: 10.1002/alz.12638

[ref9] Alzheimer’s Association (2024). 2024 Alzheimer's disease facts and figures. Alzheimers Dement. 20, 3708–3821. doi: 10.1002/alz.13809, PMID: 38689398 PMC11095490

[ref10] Andrew M. K. Tierney M. C. (2018). The puzzle of sex, gender and Alzheimer’s disease: why are women more often affected than men? Womens Health 14:1745506518817995. doi: 10.1177/1745506518817995

[ref11] Babiloni C. Arakaki X. Azami H. Bennys K. Blinowska K. Bonanni L. . (2021). Measures of resting state EEG rhythms for clinical trials in Alzheimer's disease: recommendations of an expert panel. Alzheimers Dement. 17, 1528–1553. doi: 10.1002/alz.12311, PMID: 33860614 PMC8647863

[ref12] Babiloni C. Blinowska K. Bonanni L. Cichocki A. De Haan W. Del Percio C. . (2020). What electrophysiology tells us about Alzheimer's disease: a window into the synchronization and connectivity of brain neurons. Neurobiol. Aging 85, 58–73. doi: 10.1016/j.neurobiolaging.2019.09.008, PMID: 31739167

[ref13] Babiloni C. Del Percio C. Lizio R. Noce G. Lopez S. Soricelli A. . (2018a). Abnormalities of resting-state functional cortical connectivity in patients with dementia due to Alzheimer's and Lewy body diseases: an EEG study. Neurobiol. Aging 65, 18–40. doi: 10.1016/j.neurobiolaging.2017.12.023 29407464

[ref14] Babiloni C. Del Percio C. Lizio R. Noce G. Lopez S. Soricelli A. . (2018b). Functional cortical source connectivity of resting state electroencephalographic alpha rhythms shows similar abnormalities in patients with mild cognitive impairment due to Alzheimer’s and Parkinson’s diseases. Clin. Neurophysiol. 129, 766–782. doi: 10.1016/j.clinph.2018.01.009, PMID: 29448151

[ref15] Babiloni C. Del Percio C. Pascarelli M. T. Lizio R. Noce G. Lopez S. . (2019). Abnormalities of functional cortical source connectivity of resting-state electroencephalographic alpha rhythms are similar in patients with mild cognitive impairment due to Alzheimer's and Lewy body diseases. Neurobiol. Aging 77, 112–127. doi: 10.1016/j.neurobiolaging.2019.01.013, PMID: 30797169

[ref16] Babiloni C. Frisoni G. Vecchio F. Lizio R. Pievani M. Geroldi C. . (2009). Global functional coupling of resting EEG rhythms is abnormal in mild cognitive impairment and Alzheimer’s disease: a multicenter EEG study. J. Psychophysiol. 23, 224–234. doi: 10.1027/0269-8803.23.4.224

[ref17] Babiloni C. Lizio R. Marzano N. Capotosto P. Soricelli A. Triggiani A. I. . (2016a). Brain neural synchronization and functional coupling in Alzheimer's disease as revealed by resting state EEG rhythms. Int. J. Psychophysiol. 103, 88–102. doi: 10.1016/j.ijpsycho.2015.02.008, PMID: 25660305

[ref18] Babiloni C. Triggiani A. I. Lizio R. Cordone S. Tattoli G. Bevilacqua V. . (2016b). Classification of single normal and Alzheimer's disease individuals from cortical sources of resting state EEG rhythms. Front. Neurosci. 10:47. doi: 10.3389/fnins.2016.00047, PMID: 26941594 PMC4763025

[ref19] Bagattini C. Esposito M. Ferrari C. Mazza V. Brignani D. (2022). Connectivity alterations underlying the breakdown of pseudoneglect: new insights from healthy and pathological aging. Front. Aging Neurosci. 14:930877. doi: 10.3389/fnagi.2022.930877, PMID: 36118681 PMC9475001

[ref20] Barzegaran E. van Damme B. Meuli R. Knyazeva M. G. (2016). Perception-related EEG is more sensitive to Alzheimer's disease effects than resting EEG. Neurobiol. Aging 43, 129–139. doi: 10.1016/j.neurobiolaging.2016.03.032, PMID: 27255822

[ref21] Bassett D. S. Bullmore E. (2006). Small-world brain networks. Neuroscientist 12, 512–523. doi: 10.1177/1073858406293182 17079517

[ref22] Bassett D. S. Bullmore E. T. (2017). Small-world brain networks revisited. Neuroscientist 23, 499–516. doi: 10.1177/1073858416667720, PMID: 27655008 PMC5603984

[ref23] Bassett D. S. Sporns O. (2017). Network neuroscience. Nat. Neurosci. 20, 353–364. doi: 10.1038/nn.4502, PMID: 28230844 PMC5485642

[ref24] Bassett D. S. Zurn P. Gold J. I. (2018). On the nature and use of models in network neuroscience. Nat. Rev. Neurosci. 19, 566–578. doi: 10.1038/s41583-018-0038-8, PMID: 30002509 PMC6466618

[ref25] Bastos A. M. Schoffelen J.-M. (2016). A tutorial review of functional connectivity analysis methods and their interpretational pitfalls. Front. Syst. Neurosci. 9:175. doi: 10.3389/fnsys.2015.00175, PMID: 26778976 PMC4705224

[ref26] Bazanova O. Vernon D. (2014). Interpreting EEG alpha activity. Neurosci. Biobehav. Rev. 44, 94–110. doi: 10.1016/j.neubiorev.2013.05.007, PMID: 23701947

[ref27] Birba A. Fittipaldi S. Cediel Escobar J. C. Gonzalez Campo C. Legaz A. Galiani A. . (2022). Multimodal neurocognitive markers of naturalistic discourse typify diverse neurodegenerative diseases. Cereb. Cortex 32, 3377–3391. doi: 10.1093/cercor/bhab421, PMID: 34875690 PMC9376869

[ref28] Blinowska K. J. Rakowski F. Kaminski M. Fallani F. D. V. Del Percio C. Lizio R. . (2017). Functional and effective brain connectivity for discrimination between Alzheimer’s patients and healthy individuals: a study on resting state EEG rhythms. Clin. Neurophysiol. 128, 667–680. doi: 10.1016/j.clinph.2016.10.002, PMID: 27836429

[ref29] Bonanni L. Moretti D. Benussi A. Ferri L. Russo M. Carrarini C. . (2021). Hyperconnectivity in dementia is early and focal and wanes with progression. Cereb. Cortex 31, 97–105. doi: 10.1093/cercor/bhaa209, PMID: 32797208

[ref30] Bondi M. W. Houston W. S. Eyler L. T. Brown G. G. (2005). fMRI evidence of compensatory mechanisms in older adults at genetic risk for Alzheimer disease. Neurology 64, 501–508. doi: 10.1212/01.WNL.0000150885.00929.7E, PMID: 15699382 PMC1761695

[ref31] Bruscoli M. Lovestone S. (2004). Is MCI really just early dementia? A systematic review of conversion studies. Int. Psychogeriatr. 16, 129–140. doi: 10.1017/S1041610204000092, PMID: 15318760

[ref32] Bullmore E. T. Bassett D. S. (2011). Brain graphs: graphical models of the human brain connectome. Annu. Rev. Clin. Psychol. 7, 113–140. doi: 10.1146/annurev-clinpsy-040510-143934, PMID: 21128784

[ref33] Buzi G. Fornari C. Perinelli A. Mazza V. (2023). Functional connectivity changes in mild cognitive impairment: a meta-analysis of M/EEG studies. Clin. Neurophysiol. 156, 183–195. doi: 10.1016/j.clinph.2023.10.011, PMID: 37967512

[ref34] Buzsáki G. Watson B. O. (2012). Brain rhythms and neural syntax: implications for efficient coding of cognitive content and neuropsychiatric disease. Dialogues Clin. Neurosci. 14, 345–367. doi: 10.31887/DCNS.2012.14.4/gbuzsaki, PMID: 23393413 PMC3553572

[ref35] Buzzell G. A. Niu Y. Aviyente S. Bernat E. (2022). A practical introduction to EEG time-frequency principal components analysis (TF-PCA). Dev. Cogn. Neurosci. 55:101114. doi: 10.1016/j.dcn.2022.101114, PMID: 35636345 PMC9156873

[ref36] Cabeza R. (2002). Hemispheric asymmetry reduction in older adults: the HAROLD model. Psychol. Aging 17, 85–100. doi: 10.1037/0882-7974.17.1.85, PMID: 11931290

[ref37] Cabeza R. Anderson N. D. Locantore J. K. McIntosh A. R. (2002). Aging gracefully: compensatory brain activity in high-performing older adults. NeuroImage 17, 1394–1402. doi: 10.1006/nimg.2002.1280, PMID: 12414279

[ref38] Cai L. Wei X. Liu J. Zhu L. Wang J. Deng B. . (2020). Functional integration and segregation in multiplex brain networks for Alzheimer's disease. Front. Neurosci. 14:51. doi: 10.3389/fnins.2020.00051, PMID: 32132892 PMC7040198

[ref39] Cai L. Wei X. Wang J. Yu H. Deng B. Wang R. (2018). Reconstruction of functional brain network in Alzheimer's disease via cross-frequency phase synchronization. Neurocomputing 314, 490–500. doi: 10.1016/j.neucom.2018.07.019

[ref40] Canolty R. T. Knight R. T. (2010). The functional role of cross-frequency coupling. Trends Cogn. Sci. 14, 506–515. doi: 10.1016/j.tics.2010.09.001, PMID: 20932795 PMC3359652

[ref41] Cantero J. L. Atienza M. Cruz-Vadell A. Suarez-Gonzalez A. Gil-Neciga E. (2009a). Increased synchronization and decreased neural complexity underlie thalamocortical oscillatory dynamics in mild cognitive impairment. NeuroImage 46, 938–948. doi: 10.1016/j.neuroimage.2009.03.018, PMID: 19303446

[ref42] Cantero J. L. Atienza M. Gomez-Herrero G. Cruz-Vadell A. Gil-Neciga E. Rodriguez-Romero R. . (2009b). Functional integrity of thalamocortical circuits differentiates normal aging from mild cognitive impairment. Hum. Brain Mapp. 30, 3944–3957. doi: 10.1002/hbm.20819, PMID: 19449329 PMC6871053

[ref43] Canuet L. Tellado I. Couceiro V. Fraile C. Fernandez-Novoa L. Ishii R. . (2012). Resting-state network disruption and APOE genotype in Alzheimer's disease: a lagged functional connectivity study. PLoS One 7:46289. doi: 10.1371/journal.pone.0046289, PMID: 23050006 PMC3457973

[ref44] Cao J. Zhao Y. Shan X. Wei H. L. Guo Y. Chen L. . (2022). Brain functional and effective connectivity based on electroencephalography recordings: a review. Hum. Brain Mapp. 43, 860–879. doi: 10.1002/hbm.25683, PMID: 34668603 PMC8720201

[ref45] Cassani R. Estarellas M. San-Martin R. Fraga F. J. Falk T. H. (2018). Systematic review on resting-state EEG for Alzheimer’s disease diagnosis and progression assessment. Dis. Markers 2018:5174815. doi: 10.1155/2018/5174815, PMID: 30405860 PMC6200063

[ref46] Cecchetti G. Agosta F. Basaia S. Cividini C. Cursi M. Santangelo R. . (2021). Resting-state electroencephalographic biomarkers of Alzheimer’s disease. NeuroImage 31:102711. doi: 10.1016/j.nicl.2021.102711, PMID: 34098525 PMC8185302

[ref47] Chan H.-L. Chu J.-H. Fung H.-C. Tsai Y.-T. Meng L.-F. Huang C.-C. . (2013). Brain connectivity of patients with Alzheimer's disease by coherence and cross mutual information of electroencephalograms during photic stimulation. Med. Eng. Phys. 35, 241–252. doi: 10.1016/j.medengphy.2011.10.005, PMID: 22041127

[ref48] Chapeton J. I. Haque R. Wittig J. H. Inati S. K. Zaghloul K. A. (2019). Large-scale communication in the human brain is rhythmically modulated through alpha coherence. Curr. Biol. 29:2801-2811. e2805. doi: 10.1016/j.cub.2019.07.014, PMID: 31422882 PMC6736747

[ref49] Chiarion G. Sparacino L. Antonacci Y. Faes L. Mesin L. (2023). Connectivity analysis in EEG data: a tutorial review of the state of the art and emerging trends. Bioengineering 10:372. doi: 10.3390/bioengineering10030372, PMID: 36978763 PMC10044923

[ref50] Choi K.-M. Kim J.-Y. Kim Y.-W. Han J.-W. Im C.-H. Lee S.-H. (2021). Comparative analysis of default mode networks in major psychiatric disorders using resting-state EEG. Sci. Rep. 11:22007. doi: 10.1038/s41598-021-00975-3, PMID: 34759276 PMC8580995

[ref51] Chun C. T. Seward K. Patterson A. Melton A. MacDonald-Wicks L. (2021). Evaluation of available cognitive tools used to measure mild cognitive decline: a scoping review. Nutrients 13:3974. doi: 10.3390/nu13113974, PMID: 34836228 PMC8623828

[ref52] Cohen M. X. (2014). Analyzing neural time series data: Theory and practice. Cambridge, MA: MIT Press.

[ref53] Correro A. N. Nielson K. A. (2020). A review of minority stress as a risk factor for cognitive decline in lesbian, gay, bisexual, and transgender (LGBT) elders. J. Gay Lesbian Ment. Health 24, 2–19. doi: 10.1080/19359705.2019.1644570, PMID: 33014237 PMC7531820

[ref54] Crook-Rumsey M. Howard C. J. Doborjeh Z. Doborjeh M. Ramos J. I. E. Kasabov N. . (2023). Spatiotemporal EEG dynamics of prospective memory in ageing and mild cognitive impairment. Cogn. Comput., 15, 1273–1299. doi: 10.1007/s12559-022-10075-7

[ref55] Das S. Puthankattil S. D. (2020). Complex network analysis of MCI-AD EEG signals under cognitive and resting state. Brain Res. 1735:146743. doi: 10.1016/j.brainres.2020.146743, PMID: 32114060

[ref9001] Dattola S. Mammone N. Morabito F. C. Rosaci D. Sarné G. M. L. La Foresta F. (2021). Testing graph robustness indexes for EEG analysis in Alzheimer’s disease diagnosis. Electronics 10:1440. doi: 10.3390/electronics10121440

[ref56] Dauwels J. Vialatte F. Cichocki A. (2010). Diagnosis of Alzheimer's disease from EEG signals: where are we standing? Curr. Alzheimer Res. 7, 487–505. doi: 10.2174/156720510792231720, PMID: 20455865

[ref57] Davis S. W. Dennis N. A. Daselaar S. M. Fleck M. S. Cabeza R. (2008). Que PASA? The posterior–anterior shift in aging. Cereb. Cortex 18, 1201–1209. doi: 10.1093/cercor/bhm155, PMID: 17925295 PMC2760260

[ref58] Delbeuck X. Van der Linden M. Collette F. (2003). Alzheimer'disease as a disconnection syndrome? Neuropsychol. Rev. 13, 79–92. doi: 10.1023/A:1023832305702 12887040

[ref59] Delorme A. Makeig S. (2004). EEGLAB: an open source toolbox for analysis of single-trial EEG dynamics including independent component analysis. J. Neurosci. Methods 134, 9–21. doi: 10.1016/j.jneumeth.2003.10.009, PMID: 15102499

[ref60] Dimitriadis S. I. Laskaris N. A. Bitzidou M. P. Tarnanas I. Tsolaki M. N. (2015). A novel biomarker of amnestic MCI based on dynamic cross-frequency coupling patterns during cognitive brain responses. Front. Neurosci. 9:350. doi: 10.3389/fnins.2015.00350 26539070 PMC4611062

[ref61] Ding Y. Chu Y. Liu M. Ling Z. Wang S. Li X. . (2022). Fully automated discrimination of Alzheimer’s disease using resting-state electroencephalography signals. Quant. Imaging Med. Surg. 12, 1063–1078. doi: 10.21037/qims-21-430, PMID: 35111605 PMC8739099

[ref62] Duan F. Huang Z. Sun Z. Zhang Y. Zhao Q. Cichocki A. . (2020). Topological network analysis of early Alzheimer’s disease based on resting-state EEG. IEEE Trans. Neural Syst. Rehabil. Eng. 28, 2164–2172. doi: 10.1109/TNSRE.2020.3014951, PMID: 32763856

[ref63] Dubovik S. Bouzerda-Wahlen A. Nahum L. Gold G. Schnider A. Guggisberg A. G. (2013). Adaptive reorganization of cortical networks in Alzheimer’s disease. Clin. Neurophysiol. 124, 35–43. doi: 10.1016/j.clinph.2012.05.028, PMID: 22781497

[ref64] Elverman K. H. Paitel E. R. Figueroa C. M. McKindles R. J. Nielson K. A. (2021). Event-related potentials, inhibition, and risk for Alzheimer’s disease among cognitively intact elders. J. Alzheimers Dis. 80, 1413–1428. doi: 10.3233/JAD-201559, PMID: 33682720

[ref65] Engels M. Stam C. J. van der Flier W. M. Scheltens P. de Waal H. van Straaten E. C. (2015). Declining functional connectivity and changing hub locations in Alzheimer’s disease: an EEG study. BMC Neurol. 15, 145–148. doi: 10.1186/s12883-015-0400-7, PMID: 26289045 PMC4545875

[ref66] Escudero J. Smith K. Azami H. Abásolo D. (2016). “Inspection of short-time resting-state electroencephalogram functional networks in Alzheimer's disease” in 2016 38th annual international conference of the IEEE engineering in medicine and biology society (EMBC): IEEE, 2810–2813. 10.1109/EMBC.2016.7591314 28268902

[ref67] Ferré P. Benhajali Y. Steffener J. Stern Y. Joanette Y. Bellec P. (2019). Resting-state and vocabulary tasks distinctively inform on age-related differences in the functional brain connectome. Language, cognition and neuroscience 34, 949–972. doi: 10.1080/23273798.2019.1608072, PMID: 31457069 PMC6711486

[ref68] Ferreira L. K. Busatto G. F. (2013). Resting-state functional connectivity in normal brain aging. Neurosci. Biobehav. Rev. 37, 384–400. doi: 10.1016/j.neubiorev.2013.01.017, PMID: 23333262

[ref69] Ferreri F. Vecchio F. Vollero L. Guerra A. Petrichella S. Ponzo D. . (2016). Sensorimotor cortex excitability and connectivity in Alzheimer's disease: a TMS-EEG co-registration study. Hum. Brain Mapp. 37, 2083–2096. doi: 10.1002/hbm.23158, PMID: 26945686 PMC6867580

[ref70] Fiandaca M. S. Mapstone M. E. Cheema A. K. Federoff H. J. (2014). The critical need for defining preclinical biomarkers in Alzheimer's disease. Alzheimers Dement. 10, S196–S212. doi: 10.1016/j.jalz.2014.04.015 24924671

[ref71] Fide E. Hünerli-Gündüz D. Öztura İ. Yener G. G. (2022). Hyperconnectivity matters in early-onset Alzheimer's disease: a resting-state EEG connectivity study. Neurophysiol. Clin. 52, 459–471. doi: 10.1016/j.neucli.2022.10.003, PMID: 36372646

[ref72] Fide E. Yerlikaya D. Güntekin B. Babiloni C. Yener G. G. (2023). Coherence in event-related EEG oscillations in patients with Alzheimer’s disease dementia and amnestic mild cognitive impairment. Cogn. Neurodyn. 17, 1621–1635. doi: 10.1007/s11571-022-09920-0, PMID: 37974589 PMC10640558

[ref73] Filippi M. Basaia S. Canu E. Imperiale F. Meani A. Caso F. . (2017). Brain network connectivity differs in early-onset neurodegenerative dementia. Neurology 89, 1764–1772. doi: 10.1212/WNL.0000000000004577, PMID: 28954876 PMC5664301

[ref74] Filippini N. Ebmeier K. P. MacIntosh B. J. Trachtenberg A. J. Frisoni G. B. Wilcock G. . (2011). Differential effects of the APOE genotype on brain function across the lifespan. NeuroImage 54, 602–610. doi: 10.1016/j.neuroimage.2010.08.009, PMID: 20705142

[ref75] Fischer M. H. F. Zibrandtsen I. C. Høgh P. Musaeus C. S. (2023). Systematic review of EEG coherence in Alzheimer’s disease. J. Alzheimers Dis. 91, 1261–1272. doi: 10.3233/JAD-220508, PMID: 36641665

[ref76] Folstein M. F. Robins L. N. Helzer J. E. (1983). The mini-mental state examination. Arch. Gen. Psychiatry 40:812. doi: 10.1001/archpsyc.1983.01790060110016 6860082

[ref77] Franciotti R. Falasca N. W. Arnaldi D. Famà F. Babiloni C. Onofrj M. . (2019). Cortical network topology in prodromal and mild dementia due to Alzheimer’s disease: graph theory applied to resting state EEG. Brain Topogr. 32, 127–141. doi: 10.1007/s10548-018-0674-3, PMID: 30145728 PMC6326972

[ref78] Franciotti R. Moretti D. V. Benussi A. Ferri L. Russo M. Carrarini C. . (2022). Cortical network modularity changes along the course of frontotemporal and Alzheimer's dementing diseases. Neurobiol. Aging 110, 37–46. doi: 10.1016/j.neurobiolaging.2021.10.016, PMID: 34847523

[ref79] Frangopoulou M. S. Alimardani M. (2022). qEEG analysis in the diagnosis of Alzheimer’s disease: a comparison of functional connectivity and spectral analysis. Appl. Sci. 12:5162. doi: 10.3390/app12105162

[ref80] Frantzidis C. A. Vivas A. B. Tsolaki A. Klados M. A. Tsolaki M. Bamidis P. D. (2014). Functional disorganization of small-world brain networks in mild Alzheimer's disease and amnestic mild cognitive impairment: an EEG study using relative wavelet entropy (RWE). Front. Aging Neurosci. 6:224. doi: 10.3389/fnagi.2014.00224, PMID: 25206333 PMC4144118

[ref81] Gómez C. Ruiz-Gómez S. J. Poza J. Maturana-Candelas A. Núñez P. Pinto N. . (2018). “Assessment of EEG connectivity patterns in mild cognitive impairment using phase slope index” in in: *2018 40th Annual International Conference of the IEEE Engineering in Medicine and Biology Society (EMBC)* (Honolulu, HI: IEEE), 263–266. 10.1109/EMBC.2018.8512270 30440388

[ref82] Gonzalez-Escamilla G. Atienza M. Cantero J. L. (2015). Impaired cortical oscillatory coupling in mild cognitive impairment: anatomical substrate and ApoE4 effects. Brain Struct. Funct. 220, 1721–1737. doi: 10.1007/s00429-014-0757-1, PMID: 24682246

[ref83] Gonzalez-Escamilla G. Atienza M. Garcia-Solis D. Cantero J. L. (2016). Cerebral and blood correlates of reduced functional connectivity in mild cognitive impairment. Brain Struct. Funct. 221, 631–645. doi: 10.1007/s00429-014-0930-6, PMID: 25366971

[ref84] Gour N. Felician O. Didic M. Koric L. Gueriot C. Chanoine V. . (2014). Functional connectivity changes differ in early and late-onset alzheimer's disease. Hum. Brain Mapp. 35, 2978–2994. doi: 10.1002/hbm.22379, PMID: 24123475 PMC6869697

[ref85] Güntekin B. Saatçi E. Yener G. (2008). Decrease of evoked delta, theta and alpha coherences in Alzheimer patients during a visual oddball paradigm. Brain Res. 1235, 109–116. doi: 10.1016/j.brainres.2008.06.028, PMID: 18598686

[ref86] Guo Y. Dang G. Hordacre B. Su X. Yan N. Chen S. . (2021). Repetitive transcranial magnetic stimulation of the dorsolateral prefrontal cortex modulates electroencephalographic functional connectivity in Alzheimer’s disease. Front. Aging Neurosci. 13:679585. doi: 10.3389/fnagi.2021.679585, PMID: 34305567 PMC8293898

[ref87] Gurja J. P. Muthukrishnan S. P. Tripathi M. Sharma R. (2022). Reduced resting-state cortical alpha connectivity reflects distinct functional brain Dysconnectivity in Alzheimer's disease and mild cognitive impairment. Brain Connect. 12, 134–145. doi: 10.1089/brain.2020.0926 34030487

[ref88] Hamilton C. A. Schumacher J. Matthews F. Taylor J.-P. Allan L. Barnett N. . (2021). Slowing on quantitative EEG is associated with transition to dementia in mild cognitive impairment. Int. Psychogeriatr. 33, 1321–1325. doi: 10.1017/S1041610221001083, PMID: 34551831

[ref89] Han Y. Wang K. Jia J. Wu W. (2017). Changes of EEG spectra and functional connectivity during an object-location memory task in Alzheimer’s disease. Front. Behav. Neurosci. 11:107. doi: 10.3389/fnbeh.2017.00107, PMID: 28620287 PMC5449767

[ref90] Handayani N. Haryanto F. Khotimah S. N. Arif I. Taruno W. P. (2018). Coherence and phase synchrony analyses of EEG signals in mild cognitive impairment (MCI): a study of functional brain connectivity. Polish J. Med. Physics Eng. 24, 1–9. doi: 10.2478/pjmpe-2018-0001

[ref91] Hata M. Kazui H. Tanaka T. Ishii R. Canuet L. Pascual-Marqui R. D. . (2016). Functional connectivity assessed by resting state EEG correlates with cognitive decline of Alzheimer’s disease–an eLORETA study. Clin. Neurophysiol. 127, 1269–1278. doi: 10.1016/j.clinph.2015.10.030, PMID: 26541308

[ref92] Herzog R. Rosas F. E. Whelan R. Fittipaldi S. Santamaria-Garcia H. Cruzat J. . (2022). Genuine high-order interactions in brain networks and neurodegeneration. Neurobiol. Dis. 175:105918. doi: 10.1016/j.nbd.2022.105918, PMID: 36375407 PMC11195446

[ref93] Hidasi Z. Czigler B. Salacz P. Csibri É. Molnár M. (2007). Changes of EEG spectra and coherence following performance in a cognitive task in Alzheimer's disease. Int. J. Psychophysiol. 65, 252–260. doi: 10.1016/j.ijpsycho.2007.05.002, PMID: 17586077

[ref94] Ho M.-C. Chen T.-C. Huang C.-F. Yu C.-H. Chen J.-M. Huang R.-Y. . (2014). Detect AD patients by using EEG coherence analysis. J. Med. Eng. 2014, 1–5. doi: 10.1155/2014/236734, PMID: 27006929 PMC4782614

[ref95] Horvath A. Szucs A. Csukly G. Sakovics A. Stefanics G. Kamondi A. (2018). EEG and ERP biomarkers of Alzheimer's disease: a critical review. Front. Biosci. 23, 183–220. doi: 10.2741/4587, PMID: 28930543

[ref96] Hsieh S. Lin Y.-C. (2017a). Stopping ability in younger and older adults: behavioral and event-related potential. Cogn. Affect. Behav. Neurosci. 17, 348–363. doi: 10.3758/s13415-016-0483-7, PMID: 27896714

[ref97] Hsieh S. Lin Y.-C. (2017b). Strategies for stimulus selective stopping in the elderly. Acta Psychol. 173, 122–131. doi: 10.1016/j.actpsy.2016.12.011, PMID: 28063944

[ref98] Invitto S. Piraino G. Ciccarese V. Carmillo L. Caggiula M. Trianni G. . (2018). Potential role of OERP as early marker of mild cognitive impairment. Front. Aging Neurosci. 10:272. doi: 10.3389/fnagi.2018.00272, PMID: 30271339 PMC6146232

[ref99] Ioulietta L. Kostas G. Spiros N. Vangelis O. P. Anthoula T. Ioannis K. . (2020). A novel connectome-based electrophysiological study of subjective cognitive decline related to Alzheimer’s disease by using resting-state high-density EEG EGI GES 300. Brain Sci. 10:392. doi: 10.3390/brainsci10060392, PMID: 32575641 PMC7349850

[ref100] Iragui V. J. Kutas M. Mitchiner M. R. Hillyard S. A. (1993). Effects of aging on event-related brain potentials and reaction times in an auditory oddball task. Psychophysiology 30, 10–22. doi: 10.1111/j.1469-8986.1993.tb03200.x, PMID: 8416055

[ref101] Jalili M. (2016). Functional brain networks: does the choice of dependency estimator and binarization method matter? Sci. Rep. 6, 1–12. doi: 10.1038/srep29780, PMID: 27417262 PMC4945914

[ref102] Jalili M. (2017). Graph theoretical analysis of Alzheimer's disease: discrimination of AD patients from healthy subjects. Inf. Sci. 384, 145–156. doi: 10.1016/j.ins.2016.08.047

[ref103] Jelic V. Julin P. Shigeta M. Nordberg A. Lannfelt L. Winblad B. . (1997). Apolipoprotein E ε4 allele decreases functional connectivity in Alzheimer’s disease as measured by EEG coherence. J. Neurol. Neurosurg. Psychiatry 63, 59–65. doi: 10.1136/jnnp.63.1.59, PMID: 9221969 PMC2169641

[ref104] Jeong J. Gore J. C. Peterson B. S. (2001). Mutual information analysis of the EEG in patients with Alzheimer's disease. Clin. Neurophysiol. 112, 827–835. doi: 10.1016/S1388-2457(01)00513-2, PMID: 11336898

[ref105] Jiang Z.-Y. (2005). Study on EEG power and coherence in patients with mild cognitive impairment during working memory task. J Zhejiang Univ Sci B 6, 1213–1219. doi: 10.1631/jzus.2005.B1213, PMID: 16358382 PMC1390647

[ref106] Jiang Y. Zhang X. Guo Z. Jiang N. (2024). Altered EEG Theta and alpha band functional connectivity in mild cognitive impairment during working memory coding. IEEE Trans. Neural Syst. Rehabil. Eng. 32, 2845–2853. doi: 10.1109/TNSRE.2024.3417617, PMID: 38905095

[ref107] Jiang Z.-Y. Zheng L. L. (2006). Inter-and intra-hemispheric EEG coherence in patients with mild cognitive impairment at rest and during working memory task. J. Zhejiang Univ. 7, 357–364. doi: 10.1631/jzus.2006.B0357, PMID: 16615165 PMC1462929

[ref108] Jiang Z.-Y. Zheng L.-L. Yu E.-Y. (2008). EEG coherence characteristics at rest and during a three-level working memory task in normal aging and mild cognitive impairment. Med. Sci. Monitor 14:CR515-523. Available at: http://www.medscimonit.com/abstract/index/idArt/869414, PMID: 18830191

[ref109] Josefsson A. Ibáñez A. Parra M. Escudero J. (2019). Network analysis through the use of joint-distribution entropy on EEG recordings of MCI patients during a visual short-term memory binding task. Healthcare Technol. Letters 6, 27–31. doi: 10.1049/htl.2018.5060, PMID: 31119035 PMC6498400

[ref110] Kabbara A. Eid H. El Falou W. Khalil M. Wendling F. Hassan M. (2018). Reduced integration and improved segregation of functional brain networks in Alzheimer’s disease. J. Neural Eng. 15:026023. doi: 10.1088/1741-2552/aaaa76, PMID: 29451125

[ref111] Khadem A. Hossein-Zadeh G.-A. (2014). Quantification of the effects of volume conduction on the EEG/MEG connectivity estimates: an index of sensitivity to brain interactions. Physiol. Meas. 35, 2149–2164. doi: 10.1088/0967-3334/35/10/2149, PMID: 25243864

[ref112] Kim H.-R. Go H.-J. Kim S.-Y. (2018). Discrimination of mild Alzheimer’s disease patients using cluster analysis of information transmission in EEG. J. Korean Phys. Soc. 73, 377–387. doi: 10.3938/jkps.73.377

[ref113] Klimesch W. (1999). EEG alpha and theta oscillations reflect cognitive and memory performance: a review and analysis. Brain Res. Rev. 29, 169–195. doi: 10.1016/S0165-0173(98)00056-3, PMID: 10209231

[ref114] Knyazeva M. G. Carmeli C. Khadivi A. Ghika J. Meuli R. Frackowiak R. S. (2013). Evolution of source EEG synchronization in early Alzheimer's disease. Neurobiol. Aging 34, 694–705. doi: 10.1016/j.neurobiolaging.2012.07.012, PMID: 22902196

[ref115] Knyazeva M. G. Jalili M. Brioschi A. Bourquin I. Fornari E. Hasler M. . (2010). Topography of EEG multivariate phase synchronization in early Alzheimer's disease. Neurobiol. Aging 31, 1132–1144. doi: 10.1016/j.neurobiolaging.2008.07.019, PMID: 18774201

[ref9002] Koenig T. Prichep L. Dierks T. Hubl D. Wahlund L. O. John E. R. . (2005). Decreased EEG synchronization in Alzheimer’s disease and mild cognitive impairment. Neurobiol. Aging 26, 165–171. doi: 10.1016/j.neurobiolaging.2004.03.008 15582746

[ref116] Kvitting A. S. Fällman K. Wressle E. Marcusson J. (2019). Age-normative MMSE data for older persons aged 85 to 93 in a longitudinal Swedish cohort. J. Am. Geriatr. Soc. 67, 534–538. doi: 10.1111/jgs.15694, PMID: 30536796 PMC6949533

[ref117] La Foresta F. Morabito F. C. Marino S. Dattola S. (2019). High-density EEG signal processing based on active-source reconstruction for brain network analysis in Alzheimer’s disease. Electronics 8:8091031. doi: 10.3390/electronics8091031

[ref118] Lazarou I. Georgiadis K. Nikolopoulos S. Oikonomou V. P. Stavropoulos T. G. Tsolaki A. . (2022). Exploring network properties across preclinical stages of Alzheimer’s disease using a visual short-term memory and attention task with high-density electroencephalography: a brain-connectome neurophysiological study. J. Alzheimers Dis. 87, 643–664. doi: 10.3233/JAD-215421, PMID: 35367964 PMC9661343

[ref119] Lejko N. Larabi D. I. Herrmann C. S. Aleman A. Ćurčić-Blake B. (2020). Alpha power and functional connectivity in cognitive decline: a systematic review and meta-analysis. J. Alzheimers Dis. 78, 1047–1088. doi: 10.3233/JAD-200962, PMID: 33185607 PMC7739973

[ref120] Leuchter A. Dunkin J. Lufkin R. B. Anzai Y. Cook I. A. Newton T. F. (1994). Effect of white matter disease on functional connections in the aging brain. J. Neurol. Neurosurg. Psychiatry 57, 1347–1354. doi: 10.1136/jnnp.57.11.1347, PMID: 7964810 PMC1073185

[ref121] Li R. Nguyen T. Potter T. Zhang Y. (2019). Dynamic cortical connectivity alterations associated with Alzheimer's disease: an EEG and fNIRS integration study. NeuroImage 21:101622. doi: 10.1016/j.nicl.2018.101622, PMID: 30527906 PMC6411655

[ref122] Li X. Yang C. Xie P. Han Y. Su R. Li Z. . (2021). The diagnosis of amnestic mild cognitive impairment by combining the characteristics of brain functional network and support vector machine classifier. J. Neurosci. Methods 363:109334. doi: 10.1016/j.jneumeth.2021.109334, PMID: 34428513

[ref123] Liu C. J. Huang C. F. Chou C. Y. Kuo W. J. Lin Y. T. Hung C. M. . (2012). Age-and disease-related features of task-related brain oscillations by using mutual information. Brain Behavior 2, 754–762. doi: 10.1002/brb3.93, PMID: 23170238 PMC3500462

[ref124] Locatelli T. Cursi M. Liberati D. Franceschi M. Comi G. (1998). EEG coherence in Alzheimer's disease. Electroencephalogr. Clin. Neurophysiol. 106, 229–237. doi: 10.1016/S0013-4694(97)00129-6, PMID: 9743281

[ref125] Long J. M. Holtzman D. M. (2019). Alzheimer disease: an update on pathobiology and treatment strategies. Cell 179, 312–339. doi: 10.1016/j.cell.2019.09.001, PMID: 31564456 PMC6778042

[ref126] Luck S. J. (2014). An introduction to the event-related potential technique. Cambridge, MA: MIT Press.

[ref127] Maestú F. Cuesta P. Hasan O. Fernandéz A. Funke M. Schulz P. E. (2019). The importance of the validation of M/EEG with current biomarkers in Alzheimer's disease. Front. Hum. Neurosci. 13:17. doi: 10.3389/fnhum.2019.00017, PMID: 30792632 PMC6374629

[ref128] Mahjoory K. Nikulin V. V. Botrel L. Linkenkaer-Hansen K. Fato M. M. Haufe S. (2017). Consistency of EEG source localization and connectivity estimates. NeuroImage 152, 590–601. doi: 10.1016/j.neuroimage.2017.02.076, PMID: 28300640

[ref129] Mammone N. De Salvo S. Bonanno L. Ieracitano C. Marino S. Marra A. . (2018). Brain network analysis of compressive sensed high-density EEG signals in AD and MCI subjects. IEEE Trans. Industr. Inform. 15, 527–536. doi: 10.1109/TII.2018.2868431, PMID: 39573497

[ref130] Martínez-Serra R. Alonso-Nanclares L. Cho K. Giese K. P. (2022). Emerging insights into synapse dysregulation in Alzheimer’s disease. Brain Commun. 4:fcac083. doi: 10.1093/braincomms/fcac083, PMID: 35652120 PMC9149787

[ref131] Masuda N. Sakaki M. Ezaki T. Watanabe T. (2018). Clustering coefficients for correlation networks. Front. Neuroinform. 12:7. doi: 10.3389/fninf.2018.00007, PMID: 29599714 PMC5863042

[ref132] Mehraram R. Kaiser M. Cromarty R. Graziadio S. O'Brien J. T. Killen A. . (2020). Weighted network measures reveal differences between dementia types: an EEG study. Hum. Brain Mapp. 41, 1573–1590. doi: 10.1002/hbm.24896, PMID: 31816147 PMC7267959

[ref133] Mendez M. F. (2017). Early-onset Alzheimer disease. Neurol. Clin. 35, 263–281. doi: 10.1016/j.ncl.2017.01.005, PMID: 28410659 PMC5407192

[ref134] Michel C. M. Brunet D. (2019). EEG source imaging: a practical review of the analysis steps. Front. Neurol. 10:325. doi: 10.3389/fneur.2019.00325, PMID: 31019487 PMC6458265

[ref135] Michels L. Muthuraman M. Anwar A. R. Kollias S. Leh S. E. Riese F. . (2017). Changes of functional and directed resting-state connectivity are associated with neuronal oscillations, ApoE genotype and amyloid deposition in mild cognitive impairment. Front. Aging Neurosci. 9:304. doi: 10.3389/fnagi.2017.00304, PMID: 29081745 PMC5646353

[ref136] Mielke M. M. (2018). Sex and gender differences in Alzheimer’s disease dementia. Psychiatric Times 35:14. 30820070 PMC6390276

[ref137] Mielke M. M. Vemuri P. Rocca W. A. (2014). Clinical epidemiology of Alzheimer’s disease: assessing sex and gender differences. Clin. Epidemiol. 6, 37–48. doi: 10.2147/CLEP.S37929, PMID: 24470773 PMC3891487

[ref138] Milà-Alomà M. Suárez-Calvet M. Molinuevo J. L. (2019). Latest advances in cerebrospinal fluid and blood biomarkers of Alzheimer’s disease. Ther. Adv. Neurol. Disord. 12:1756286419888819. doi: 10.1177/1756286419888819, PMID: 31897088 PMC6920596

[ref139] Millett D. (2001). Hans Berger: from psychic energy to the EEG. Perspect. Biol. Med. 44, 522–542. doi: 10.1353/pbm.2001.0070, PMID: 11600799

[ref140] Miraglia F. Pappalettera C. Guglielmi V. Cacciotti A. Manenti R. Judica E. . (2023). The combination of hyperventilation test and graph theory parameters to characterize EEG changes in mild cognitive impairment (MCI) condition. Gero Sci. 45, 1857–1867. doi: 10.1007/s11357-023-00733-5, PMID: 36692591 PMC10400506

[ref141] Miraglia F. Vecchio F. Bramanti P. Rossini P. M. (2016). EEG characteristics in “eyes-open” versus “eyes-closed” conditions: small-world network architecture in healthy aging and age-related brain degeneration. Clin. Neurophysiol. 127, 1261–1268. doi: 10.1016/j.clinph.2015.07.040, PMID: 26603651

[ref142] Miraglia F. Vecchio F. Pappalettera C. Nucci L. Cotelli M. Judica E. . (2022). Brain connectivity and graph theory analysis in Alzheimer’s and Parkinson’s disease: the contribution of electrophysiological techniques. Brain Sci. 12:402. doi: 10.3390/brainsci12030402, PMID: 35326358 PMC8946843

[ref143] Montemurro S. Filippini N. Ferrazzi G. Mantini D. Arcara G. Marino M. (2023). Education differentiates cognitive performance and resting state fMRI connectivity in healthy aging. Front. Aging Neurosci. 15:1168576. doi: 10.3389/fnagi.2023.1168576, PMID: 37293663 PMC10244540

[ref144] Morales S. Bowers M. E. (2022). Time-frequency analysis methods and their application in developmental EEG data. Dev. Cogn. Neurosci. 54:101067. doi: 10.1016/j.dcn.2022.101067, PMID: 35065418 PMC8784307

[ref145] Movahed R.A. Rezaeian M. (2022). Automatic diagnosis of mild cognitive impairment based on spectral, functional connectivity, and nonlinear EEG-based features. Comput Math Methods Med. 2022:2014001. doi: 10.1155/2022/2014001 35991131 PMC9388263

[ref146] Mulert C. (2013). Simultaneous EEG and fMRI: towards the characterization of structure and dynamics of brain networks. Dialogues Clin. Neurosci. 15, 381–386. doi: 10.31887/DCNS.2013.15.3/cmulert, PMID: 24174908 PMC3811108

[ref147] Musaeus C. S. Engedal K. Høgh P. Jelic V. Mørup M. Naik M. . (2019a). Oscillatory connectivity as a diagnostic marker of dementia due to Alzheimer’s disease. Clin. Neurophysiol. 130, 1889–1899. doi: 10.1016/j.clinph.2019.07.016, PMID: 31408790

[ref148] Musaeus C. S. Nielsen M. S. Høgh P. (2019b). Altered low-frequency EEG connectivity in mild cognitive impairment as a sign of clinical progression. J. Alzheimers Dis. 68, 947–960. doi: 10.3233/JAD-181081, PMID: 30883355

[ref149] Musaeus C. S. Nielsen M. S. Musaeus J. S. Høgh P. (2020). Electroencephalographic cross-frequency coupling as a sign of disease progression in patients with mild cognitive impairment: a pilot study. Front. Neurosci. 14:790. doi: 10.3389/fnins.2020.00790, PMID: 32848563 PMC7431634

[ref150] Muthukumaraswamy S. D. (2013). High-frequency brain activity and muscle artifacts in MEG/EEG: a review and recommendations. Front. Hum. Neurosci. 7:138. doi: 10.3389/fnhum.2013.00138, PMID: 23596409 PMC3625857

[ref151] Nielson K. A. Douville K. L. Seidenberg M. Woodard J. L. Miller S. K. Franczak M. . (2006). Age-related functional recruitment for famous name recognition: an event-related fMRI study. Neurobiol. Aging 27, 1494–1504. doi: 10.1016/j.neurobiolaging.2005.08.022, PMID: 16225965 PMC2078241

[ref152] Nolte G. Bai O. Wheaton L. Mari Z. Vorbach S. Hallett M. (2004). Identifying true brain interaction from EEG data using the imaginary part of coherency. Clin. Neurophysiol. 115, 2292–2307. doi: 10.1016/j.clinph.2004.04.029, PMID: 15351371

[ref153] Núñez P. Poza J. Gómez C. Rodríguez-González V. Hillebrand A. Tewarie P. . (2021). Abnormal meta-state activation of dynamic brain networks across the Alzheimer spectrum. NeuroImage 232:117898. doi: 10.1016/j.neuroimage.2021.117898, PMID: 33621696

[ref154] Núñez P. Poza J. Gómez C. Rodríguez-González V. Hillebrand A. Tola-Arribas M. A. . (2019). Characterizing the fluctuations of dynamic resting-state electrophysiological functional connectivity: reduced neuronal coupling variability in mild cognitive impairment and dementia due to Alzheimer’s disease. J. Neural Eng. 16:056030. doi: 10.1088/1741-2552/ab234b, PMID: 31112938

[ref155] Nunez P. L. Srinivasan R. (2006). Electric fields of the brain: The neurophysics of EEG. USA: Oxford University Press.

[ref156] O’Neal M. A. (2024). Women and the risk of Alzheimer's disease. Front. Global Women's Health 4:1324522. doi: 10.3389/fgwh.2023.1324522, PMID: 38250748 PMC10796575

[ref157] Oostenveld R. Fries P. Maris E. Schoffelen J.-M. (2011). FieldTrip: open source software for advanced analysis of MEG, EEG, and invasive electrophysiological data. Comput. Intell. Neurosci. 2011:156869. doi: 10.1155/2011/156869, PMID: 21253357 PMC3021840

[ref158] Page M. J. McKenzie J. E. Bossuyt P. M. Boutron I. Hoffmann T. C. Mulrow C. D. . (2021). The PRISMA 2020 statement: an updated guideline for reporting systematic reviews. BMJ 372:n71. doi: 10.1136/bmj.n71, PMID: 33782057 PMC8005924

[ref159] Paitel E. R. Nielson K. A. (2021). Temporal dynamics of event-related potentials during inhibitory control characterize age-related neural compensation. Symmetry 13:2323. doi: 10.3390/sym13122323, PMID: 35923222 PMC9345327

[ref160] Paitel E. R. Nielson K. A. (2023). Cerebellar EEG source localization reveals age-related compensatory activity moderated by genetic risk for Alzheimer's disease. Psychophysiology 60. doi: 10.1111/psyp.14395, PMID: 37493042 PMC10720653

[ref161] Paitel E. R. Samii M. R. Nielson K. A. (2021). A systematic review of cognitive event-related potentials in mild cognitive impairment and Alzheimer’s disease. Behav. Brain Res. 396:112904. doi: 10.1016/j.bbr.2020.112904, PMID: 32941881

[ref162] Park D. C. Reuter-Lorenz P. (2009). The adaptive brain: aging and neurocognitive scaffolding. Annu. Rev. Psychol. 60, 173–196. doi: 10.1146/annurev.psych.59.103006.093656, PMID: 19035823 PMC3359129

[ref163] Penny W. D. Friston K. J. Ashburner J. T. Kiebel S. J. Nichols T. E. (2011). Statistical parametric mapping: The analysis of functional brain images. Amsterdam, The Netherlands: Elsevier.

[ref164] Peraza L. R. Cromarty R. Kobeleva X. Firbank M. J. Killen A. Graziadio S. . (2018). Electroencephalographic derived network differences in Lewy body dementia compared to Alzheimer’s disease patients. Sci. Rep. 8:4637. doi: 10.1038/s41598-018-22984-5, PMID: 29545639 PMC5854590

[ref165] Petersen R. C. (2004). Mild cognitive impairment. CONTINUUM: lifelong learning. Neurology 10, 9–28. doi: 10.1212/01.CON.0000293545.39683.cc, PMID: 37703639

[ref166] Petersen R. C. (2016). Mild cognitive impairment. CONTINUUM: lifelong learning. Neurology 22, 404–418. doi: 10.1212/CON.0000000000000313, PMID: 27042901 PMC5390929

[ref167] Petersen R. C. Lopez O. Armstrong M. J. Getchius T. S. Ganguli M. Gloss D. . (2018). Practice guideline update summary: mild cognitive impairment: report of the guideline development, dissemination, and implementation Subcommittee of the American Academy of neurology. Neurology 90, 126–135. doi: 10.1212/WNL.0000000000004826, PMID: 29282327 PMC5772157

[ref168] Pistono A. Busigny T. Jucla M. Cabirol A. Dinnat A.-L. Pariente J. . (2019). An analysis of famous person semantic memory in aging. Exp. Aging Res. 45, 74–93. doi: 10.1080/0361073X.2018.1560118, PMID: 30702032

[ref169] Pons A. J. Cantero J. L. Atienza M. Garcia-Ojalvo J. (2010). Relating structural and functional anomalous connectivity in the aging brain via neural mass modeling. NeuroImage 52, 848–861. doi: 10.1016/j.neuroimage.2009.12.105, PMID: 20056154

[ref170] Požar R. Giordani B. Kavcic V. (2020). Effective differentiation of mild cognitive impairment by functional brain graph analysis and computerized testing. PLoS One 15:e0230099. doi: 10.1371/journal.pone.0230099, PMID: 32176709 PMC7075594

[ref171] Rao S. M. Bonner-Jackson A. Nielson K. A. Seidenberg M. Smith J. C. Woodard J. L. . (2015). Genetic risk for Alzheimer's disease alters the five-year trajectory of semantic memory activation in cognitively intact elders. NeuroImage 111, 136–146. doi: 10.1016/j.neuroimage.2015.02.011, PMID: 25687593 PMC4387085

[ref172] Reuter-Lorenz P. A. Cappell K. A. (2008). Neurocognitive aging and the compensation hypothesis. Curr. Dir. Psychol. Sci. 17, 177–182. doi: 10.1111/j.1467-8721.2008.00570.x

[ref173] Reuter-Lorenz P. A. Park D. C. (2014). How does it STAC up? Revisiting the scaffolding theory of aging and cognition. Neuropsychol. Rev. 24, 355–370. doi: 10.1007/s11065-014-9270-9, PMID: 25143069 PMC4150993

[ref174] Riddle J. McFerren A. Frohlich F. (2021). Causal role of cross-frequency coupling in distinct components of cognitive control. Prog. Neurobiol. 202:102033. doi: 10.1016/j.pneurobio.2021.102033, PMID: 33741402 PMC8184612

[ref175] Rodinskaia D. Radinski C. Labuhn J. (2022). EEG coherence as a marker of functional connectivity disruption in Alzheimer's disease. Aging Health Res. 2:100098. doi: 10.1016/j.ahr.2022.100098

[ref176] Roldán-Tapia M. D. Cánovas R. León I. García-Garcia J. (2017). Cognitive vulnerability in aging may be modulated by education and reserve in healthy people. Front. Aging Neurosci. 9:340. doi: 10.3389/fnagi.2017.00340, PMID: 29118710 PMC5661171

[ref177] Rubinov M. Sporns O. (2010). Complex network measures of brain connectivity: uses and interpretations. NeuroImage 52, 1059–1069. doi: 10.1016/j.neuroimage.2009.10.003, PMID: 19819337

[ref178] Ruiz-Gómez S. J. Gómez C. Poza J. Maturana-Candelas A. Rodríguez-González V. García M. . (2019a). “Analysis of volume conduction effects on different functional connectivity metrics: application to Alzheimer’s disease EEG signals” in in: 2019 *41st Annual International Conference of the IEEE Engineering in Medicine and Biology Society (EMBC)* (Berlin, Germany: IEEE), 6434–6437. 10.1109/EMBC.2019.8856548 31947315

[ref179] Ruiz-Gómez S. J. Hornero R. Poza J. Maturana-Candelas A. Pinto N. Gómez C. (2019b). Computational modeling of the effects of EEG volume conduction on functional connectivity metrics. Application to Alzheimer’s disease continuum. J. Neural Eng. 16:066019. doi: 10.1088/1741-2552/ab4024, PMID: 31470433

[ref180] Ruiz-Gómez S. J. Hornero R. Poza J. Santamaría-Vázquez E. Rodríguez-González V. Maturana-Candelas A. . (2021). A new method to build multiplex networks using canonical correlation analysis for the characterization of the Alzheimer’s disease continuum. J. Neural Eng. 18:026002. doi: 10.1088/1741-2552/abd82c, PMID: 33395667

[ref181] Sakkalis V. (2011). Review of advanced techniques for the estimation of brain connectivity measured with EEG/MEG. Comput. Biol. Med. 41, 1110–1117. doi: 10.1016/j.compbiomed.2011.06.020, PMID: 21794851

[ref182] Sala-Llonch R. Bartrés-Faz D. Junqué C. (2015). Reorganization of brain networks in aging: a review of functional connectivity studies. Front. Psychol. 6:663. doi: 10.3389/fpsyg.2015.00663, PMID: 26052298 PMC4439539

[ref183] Salis F. Costaggiu D. Mandas A. (2023). Mini-mental state examination: optimal cut-off levels for mild and severe cognitive impairment. Geriatrics 8:12. doi: 10.3390/geriatrics8010012, PMID: 36648917 PMC9844353

[ref184] Salthouse T. A. (2014). Quantity and structure of word knowledge across adulthood. Intelligence 46, 122–130. doi: 10.1016/j.intell.2014.05.009, PMID: 24932055 PMC4050304

[ref185] Sankari Z. Adeli H. Adeli A. (2011). Intrahemispheric, interhemispheric, and distal EEG coherence in Alzheimer’s disease. Clin. Neurophysiol. 122, 897–906. doi: 10.1016/j.clinph.2010.09.008, PMID: 21056936

[ref186] Sankari Z. Adeli H. Adeli A. (2012). Wavelet coherence model for diagnosis of Alzheimer disease. Clin. EEG Neurosci. 43, 268–278. doi: 10.1177/1550059412444970, PMID: 22715491

[ref187] Schiff S. Valenti P. Andrea P. Lot M. Bisiacchi P. Gatta A. . (2008). The effect of aging on auditory components of event-related brain potentials. Clin. Neurophysiol. 119, 1795–1802. doi: 10.1016/j.clinph.2008.04.007, PMID: 18495531

[ref188] Schnitzler A. Gross J. (2005). Normal and pathological oscillatory communication in the brain. Nat. Rev. Neurosci. 6, 285–296. doi: 10.1038/nrn1650, PMID: 15803160

[ref189] Schoffelen J. M. Gross J. (2009). Source connectivity analysis with MEG and EEG. Hum. Brain Mapp. 30, 1857–1865. doi: 10.1002/hbm.20745, PMID: 19235884 PMC6870611

[ref190] Sedghizadeh M. J. Aghajan H. Vahabi Z. Fatemi S. N. Afzal A. (2022). Network synchronization deficits caused by dementia and Alzheimer’s disease serve as topographical biomarkers: a pilot study. Brain Struct. Funct. 227, 2957–2969. doi: 10.1007/s00429-022-02554-2, PMID: 35997832 PMC9396580

[ref191] Sedghizadeh M. J. Hojjati H. Ezzatdoost K. Aghajan H. Vahabi Z. Tarighatnia H. (2020). Olfactory response as a marker for Alzheimer’s disease: evidence from perceptual and frontal lobe oscillation coherence deficit. PLoS One 15:e0243535. doi: 10.1371/journal.pone.0243535, PMID: 33320870 PMC7737889

[ref192] Seidenberg M. Guidotti L. Nielson K. A. Woodard J. L. Durgerian S. Antuono P. . (2009). Semantic memory activation in individuals at risk for developing Alzheimer disease. Neurology 73, 612–620. doi: 10.1212/WNL.0b013e3181b389ad, PMID: 19704080 PMC2731619

[ref193] Selkoe D. J. Hardy J. (2016). The amyloid hypothesis of Alzheimer's disease at 25 years. EMBO Mol. Med. 8, 595–608. doi: 10.15252/emmm.201606210, PMID: 27025652 PMC4888851

[ref194] Shankar G. M. Walsh D. M. (2009). Alzheimer's disease: synaptic dysfunction and Aβ. Mol. Neurodegener. 4, 48–13. doi: 10.1186/1750-1326-4-48, PMID: 19930651 PMC2788538

[ref195] Slotnick S. D. (2017). “fMRI versus ERPs” in Cognitive neuroscience of memory (Cambridge, England: Cambridge University Press).

[ref196] Smailovic U. Jelic V. (2019). Neurophysiological markers of Alzheimer’s disease: quantitative EEG approach. Neurol. Ther. 8, 37–55. doi: 10.1007/s40120-019-00169-0, PMID: 31833023 PMC6908537

[ref197] Smith K. Abasolo D. Escudero J. (2016). “A comparison of the cluster-span threshold and the union of shortest paths as objective thresholds of EEG functional connectivity networks from Beta activity in Alzheimer’s disease” in in: 2016 38th Annual International Conference of the IEEE Engineering in Medicine and Biology Society (EMBC) ((Orlando, FL: IEEE)), 2826–2829. 10.1109/EMBC.2016.7591318 28268906

[ref198] Song J. Davey C. Poulsen C. Luu P. Turovets S. Anderson E. . (2015). EEG source localization: sensor density and head surface coverage. J. Neurosci. Methods 256, 9–21. doi: 10.1016/j.jneumeth.2015.08.015, PMID: 26300183

[ref199] Song Z. Deng B. Wang J. Wang R. (2018). Biomarkers for Alzheimer's disease defined by a novel brain functional network measure. IEEE Trans. Biomed. Eng. 66, 41–49. doi: 10.1109/TBME.2018.2834546, PMID: 29993428

[ref200] Spyrou L. Parra M. Escudero J. (2018). Complex tensor factorization with PARAFAC2 for the estimation of brain connectivity from the EEG. IEEE Trans. Neural Syst. Rehabil. Eng. 27, 1–12. doi: 10.1109/TNSRE.2018.2883514, PMID: 30507512

[ref201] Srinivasan R. Winter W. R. Ding J. Nunez P. L. (2007). EEG and MEG coherence: measures of functional connectivity at distinct spatial scales of neocortical dynamics. J. Neurosci. Methods 166, 41–52. doi: 10.1016/j.jneumeth.2007.06.026, PMID: 17698205 PMC2151962

[ref202] Stam C. J. (2014). Modern network science of neurological disorders. Nat. Rev. Neurosci. 15, 683–695. doi: 10.1038/nrn3801 25186238

[ref203] Stevens A. A. Skudlarski P. Gatenby J. C. Gore J. C. (2000). Event-related fMRI of auditory and visual oddball tasks. Magn. Reson. Imaging 18, 495–502. doi: 10.1016/S0730-725X(00)00128-4, PMID: 10913710

[ref204] Su R. Li X. Li Z. Han Y. Cui W. Xie P. . (2021). Constructing biomarker for early diagnosis of aMCI based on combination of multiscale fuzzy entropy and functional brain connectivity. Biomed. Signal Processing Control 70:103000. doi: 10.1016/j.bspc.2021.103000

[ref205] Sugarman M. A. Woodard J. L. Nielson K. A. Seidenberg M. Smith J. C. Durgerian S. . (2012). Functional magnetic resonance imaging of semantic memory as a presymptomatic biomarker of Alzheimer's disease risk. Biochimica et Biophysica Acta Molecular Basis of Disease 1822, 442–456. doi: 10.1016/j.bbadis.2011.09.016, PMID: 21996618 PMC3580153

[ref206] Sweeney-Reed C. M. Riddell P. M. Ellis J. A. Freeman J. E. Nasuto S. J. (2012). Neural correlates of true and false memory in mild cognitive impairment. PLoS One 7:e48357. doi: 10.1371/journal.pone.0048357, PMID: 23118992 PMC3485202

[ref207] Tadel F. Baillet S. Mosher J. C. Pantazis D. Leahy R. M. (2011). Brainstorm: a user-friendly application for MEG/EEG analysis. Comput. Intell. Neurosci. 2011, 1–13. doi: 10.1155/2011/879716, PMID: 21584256 PMC3090754

[ref208] Tahaei M. S. Jalili M. Knyazeva M. G. (2012). Synchronizability of EEG-based functional networks in early Alzheimer's disease. IEEE Trans. Neural Syst. Rehabil. Eng. 20, 636–641. doi: 10.1109/TNSRE.2012.2202127 22695360

[ref209] Tait L. Stothart G. Coulthard E. Brown J. T. Kazanina N. Goodfellow M. (2019). Network substrates of cognitive impairment in Alzheimer’s disease. Clin. Neurophysiol. 130, 1581–1595. doi: 10.1016/j.clinph.2019.05.027, PMID: 31306967

[ref210] Tao H.-y. Tian X. (2006). “Coherence Characteristics of Gamma-band EEG during rest and cognitive task in MCI and AD“, in: 2005 IEEE Engineering in Medicine and Biology 27th Annual Conference. Shanghai, China: IEEE 2747–2750. 10.1109/IEMBS.2005.1617040 17282809

[ref211] Teipel S. J. Grothe M. J. Zhou J. Sepulcre J. Dyrba M. Sorg C. . (2016). Measuring cortical connectivity in Alzheimer’s disease as a brain neural network pathology: toward clinical applications. J. Int. Neuropsychol. Soc. 22, 138–163. doi: 10.1017/S1355617715000995, PMID: 26888613

[ref212] Teipel S. J. Pogarell O. Meindl T. Dietrich O. Sydykova D. Hunklinger U. . (2009). Regional networks underlying interhemispheric connectivity: an EEG and DTI study in healthy ageing and amnestic mild cognitive impairment. Hum. Brain Mapp. 30, 2098–2119. doi: 10.1002/hbm.20652, PMID: 18781594 PMC6870980

[ref213] Thilaga M. Ramasamy V. Nadarajan R. Nandagopal D. (2018). Shortest path based network analysis to characterize cognitive load states of human brain using EEG based functional brain networks. J. Integr. Neurosci. 17, 133–148. doi: 10.31083/JIN-170049, PMID: 28968248

[ref9003] Timothy L. T. Krishna B. M. Nair U. (2017). Classification of mild cognitive impairment EEG using combined recurrence and cross recurrence quantification analysis. Int J Psychophysiol. 120, 86–95. doi: 10.1016/j.ijpsycho.2017.07.006 28711698

[ref214] Tóth B. File B. Boha R. Kardos Z. Hidasi Z. Gaál Z. A. . (2014). EEG network connectivity changes in mild cognitive impairment—preliminary results. Int. J. Psychophysiol. 92, 1–7. doi: 10.1016/j.ijpsycho.2014.02.001, PMID: 24508504

[ref215] Tucker A. M. Stern Y. (2011). Cognitive reserve in aging. Curr. Alzheimer Res. 8, 354–360. doi: 10.2174/156720511795745320, PMID: 21222591 PMC3135666

[ref216] Tyrer A. Gilbert J. R. Adams S. Stiles A. B. Bankole A. O. Gilchrist I. D. . (2020). Lateralized memory circuit dropout in Alzheimer’s disease patients. Brain. Communications 2:fcaa212. doi: 10.1093/braincomms/fcaa212, PMID: 33409493 PMC7772115

[ref217] Van de Steen F. Faes L. Karahan E. Songsiri J. Valdes-Sosa P. A. Marinazzo D. (2019). Critical comments on EEG sensor space dynamical connectivity analysis. Brain Topogr. 32, 643–654. doi: 10.1007/s10548-016-0538-7, PMID: 27905073

[ref218] Van Diessen E. Zweiphenning W. J. Jansen F. E. Stam C. J. Braun K. P. Otte W. M. (2014). Brain network organization in focal epilepsy: a systematic review and meta-analysis. PLoS One 9:e114606. doi: 10.1371/journal.pone.0114606, PMID: 25493432 PMC4262431

[ref219] Vanneste S. Luckey A. McLeod S. L. Robertson I. H. To, W.T (2021). Impaired posterior cingulate cortex–parahippocampus connectivity is associated with episodic memory retrieval problems in amnestic mild cognitive impairment. Eur. J. Neurosci. 53, 3125–3141. doi: 10.1111/ejn.15189, PMID: 33738836

[ref220] Vecchio F. Babiloni C. Lizio R. Fallani F. D. V. Blinowska K. Verrienti G. . (2013). Resting state cortical EEG rhythms in Alzheimer's disease: toward EEG markers for clinical applications: a review. Suppl. Clin. Neurophysiol. 62, 223–236. doi: 10.1016/B978-0-7020-5307-8.00015-6, PMID: 24053043

[ref221] Vecchio F. Miraglia F. Alù F. Judica E. Cotelli M. Pellicciari M. C. . (2022). Human brain networks in physiological and pathological aging: reproducibility of electroencephalogram graph theoretical analysis in cortical connectivity. Brain Connect. 12, 41–51. doi: 10.1089/brain.2020.0824, PMID: 33797981

[ref222] Vecchio F. Miraglia F. Alú F. Orticoni A. Judica E. Cotelli M. . (2021). Contribution of graph theory applied to EEG data analysis for Alzheimer’s disease versus vascular dementia diagnosis. J. Alzheimers Dis. 82, 871–879. doi: 10.3233/JAD-210394, PMID: 34092648

[ref223] Vecchio F. Miraglia F. Marra C. Quaranta D. Vita M. G. Bramanti P. . (2014). Human brain networks in cognitive decline: a graph theoretical analysis of cortical connectivity from EEG data. J. Alzheimers Dis. 41, 113–127. doi: 10.3233/JAD-132087, PMID: 24577480

[ref224] Vecchio F. Miraglia F. Piludu F. Granata G. Romanello R. Caulo M. . (2017). “Small world” architecture in brain connectivity and hippocampal volume in Alzheimer’s disease: a study via graph theory from EEG data. Brain Imaging Behav. 11, 473–485. doi: 10.1007/s11682-016-9528-3, PMID: 26960946

[ref225] Vecchio F. Miraglia F. Quaranta D. Granata G. Romanello R. Marra C. . (2016). Cortical connectivity and memory performance in cognitive decline: a study via graph theory from EEG data. Neuroscience 316, 143–150. doi: 10.1016/j.neuroscience.2015.12.036, PMID: 26724581

[ref226] Vecchio F. Miraglia F. Quaranta D. Lacidogna G. Marra C. Rossini P. M. (2018). Learning processes and brain connectivity in a cognitive-motor task in neurodegeneration: evidence from EEG network analysis. J. Alzheimers Dis. 66, 471–481. doi: 10.3233/JAD-180342 30282357

[ref227] Vyšata O. Vališ M. Procházka A. Rusina R. Pazdera L. (2015). Linear and nonlinear EEG synchronization in Alzheimer’s disease. Neurophysiology 47, 46–52. doi: 10.1007/s11062-015-9496-z

[ref228] Wang Y. Huang X. Feng Y. Luo Q. He Y. Guo Q. . (2022). Resting-state electroencephalography and P300 evidence: age-related vestibular loss as a risk factor contributes to cognitive decline. J. Alzheimers Dis. 86, 1107–1121. doi: 10.3233/JAD-215467, PMID: 35213376 PMC9108596

[ref229] Wang R. Wang J. Yu H. Wei X. Yang C. Deng B. (2014). Decreased coherence and functional connectivity of electroencephalograph in Alzheimer's disease. Chaos: an interdisciplinary. Journal of Nonlinear Science 24:033136. doi: 10.1063/1.4896095, PMID: 25273216

[ref230] Wang R. Wang J. Yu H. Wei X. Yang C. Deng B. (2015). Power spectral density and coherence analysis of Alzheimer’s EEG. Cogn. Neurodyn. 9, 291–304. doi: 10.1007/s11571-014-9325-x, PMID: 25972978 PMC4427585

[ref231] Ward A. Tardiff S. Dye C. Arrighi H. M. (2013). Rate of conversion from prodromal Alzheimer's disease to Alzheimer's dementia: a systematic review of the literature. Dementia Geriatric Cognitive Disorders Extra 3, 320–332. doi: 10.1159/000354370, PMID: 24174927 PMC3808216

[ref232] Watanabe H. Bagarinao E. Yokoi T. Yamaguchi H. Ishigaki S. Mausuda M. . (2019). “Tau Accumulation and Network Breakdown in Alzheimer’s Disease” in Tau Biology. Advances in Experimental Medicine and Biology. eds. Takashima A. Wolozin B. Buee L., vol. 1184 (Springer: Singapore), 231–240. doi: 10.1007/978-981-32-9358-8_19 32096042

[ref233] Wei L. Li Y. Yang X. Xue Q. Wang Y. (2015). Altered characteristic of brain networks in mild cognitive impairment during a selective attention task: an EEG study. Int. J. Psychophysiol. 98, 8–16. doi: 10.1016/j.ijpsycho.2015.05.015, PMID: 26048737

[ref234] Wells G. Shea B. O’connell D. Peterson J. Welch V. Losos M. . (2014). Newcastle-Ottawa quality assessment scale cohort studies. Ottawa, Canada: University of Ottawa.

[ref9004] Wen D. Xue Q. Lu C. Guan X. Wang Y. Li X. (2014). A global coupling index of multivariate neural series with application to the evaluation of mild cognitive impairment. Neural Netw. 56, 1–9. doi: 10.1016/j.neunet.2014.03.001 24811057

[ref235] Wijaya A. Setiawan N. A. Ahmad A. H. Zakaria R. Othman Z. (2023). Electroencephalography and mild cognitive impairment research: a scoping review and bibliometric analysis (ScoRBA). AIMS Neurosci. 10, 154–171. doi: 10.3934/Neuroscience.2023012, PMID: 37426780 PMC10323261

[ref236] Woodard J. L. Seidenberg M. Nielson K. A. Smith J. C. Antuono P. Durgerian S. . (2010). Prediction of cognitive decline in healthy older adults using fMRI. J. Alzheimers Dis. 21, 871–885. doi: 10.3233/JAD-2010-091693, PMID: 20634590 PMC2940960

[ref237] Xu P. Xiong X. C. Xue Q. Tian Y. Peng Y. Zhang R. . (2014). Recognizing mild cognitive impairment based on network connectivity analysis of resting EEG with zero reference. Physiol. Meas. 35, 1279–1298. doi: 10.1088/0967-3334/35/7/1279, PMID: 24853724

[ref238] Yan Y. Zhao A. Ying W. Qiu Y. Ding Y. Wang Y. . (2021). Functional connectivity alterations based on the weighted phase lag index: an exploratory electroencephalography study on Alzheimer’s disease. Curr. Alzheimer Res. 18, 513–522. doi: 10.2174/1567205018666211001110824, PMID: 34598666

[ref239] Youssef N. Xiao S. Liu M. Lian H. Li R. Chen X. . (2021). Functional brain networks in mild cognitive impairment based on resting electroencephalography signals. Front. Comput. Neurosci. 15:698386. doi: 10.3389/fncom.2021.698386, PMID: 34776913 PMC8579961

[ref240] Yu H. Lei X. Song Z. Liu C. Wang J. (2019). Supervised network-based fuzzy learning of EEG signals for Alzheimer's disease identification. IEEE Trans. Fuzzy Syst. 28, 60–71. doi: 10.1109/TFUZZ.2019.2903753, PMID: 39573497

[ref241] Yu H. Lei X. Song Z. Wang J. Wei X. Yu B. (2018). Functional brain connectivity in Alzheimer’s disease: an EEG study based on permutation disalignment index. Physica A 506, 1093–1103. doi: 10.1016/j.physa.2018.05.009

[ref242] Yu M. Sporns O. Saykin A. J. (2021). The human connectome in Alzheimer disease—relationship to biomarkers and genetics. Nat. Rev. Neurol. 17, 545–563. doi: 10.1038/s41582-021-00529-1, PMID: 34285392 PMC8403643

[ref243] Yu J.-T. Tan L. Hardy J. (2014). Apolipoprotein E in Alzheimer's disease: an update. Annu. Rev. Neurosci. 37, 79–100. doi: 10.1146/annurev-neuro-071013-014300, PMID: 24821312

[ref244] Zhang X. Ren H. Pei Z. Lian C. Su X. Lan X. . (2022). Dual-targeted repetitive transcranial magnetic stimulation modulates brain functional network connectivity to improve cognition in mild cognitive impairment patients. Front. Physiol. 13:2434. doi: 10.3389/fphys.2022.1066290, PMID: 36467674 PMC9716076

[ref245] Zhao Y. Zhao Y. Durongbhan P. Chen L. Liu J. Billings S. . (2019). Imaging of nonlinear and dynamic functional brain connectivity based on EEG recordings with the application on the diagnosis of Alzheimer’s disease. IEEE Trans. Med. Imaging 39, 1571–1581. doi: 10.1109/TMI.2019.2953584, PMID: 31725372

[ref246] Zheng L.-L. Jiang Z.-Y. Yu E.-Y. (2007). Alpha spectral power and coherence in the patients with mild cognitive impairment during a three-level working memory task. J Zhejiang Univ Sci B 8, 584–592. doi: 10.1631/jzus.2007.B0584, PMID: 17657862 PMC1934955

