# Supplementary material for: Functional and effective EEG connectivity patterns in Alzheimer’s disease and mild cognitive impairment: a systematic review
Source: Front Aging Neurosci. 2025 Feb 12;17:1496235. doi: 10.3389/fnagi.2025.1496235 (PMC11861106; doi:10.3389/fnagi.2025.1496235)
Supplement: Supplementary file 1 [file Data_Sheet_1.pdf]

Table S1.
Demographic data (mean(SD)) for studies comparing Mild Cognitive Impairment (MCI) and healthy control (HC) groups.

Authors (Year)	Total N^	MCI N	HC N	MCI Age	HC Age	MCI MMSE	HC MMSE	MCI EDU	HC EDU	MCI %F	HC %F	Ref.
Babiloni et al. (2009a)	206	69	64	74.1 (0.8)	73.7 (0.9)	26.6 (0.2)	28.6 (0.2)	9.8 (0.5)	9.7 (0.6)	55.1	50.0	3
Babiloni et al. (2009b)	132	52	47	72.3 (0.8)	74.7 (0.9)	25.3 (0.3)	27.2 (0.4)	7.8 (0.6)	9.4 (0.7)	51.9	46.8	4
Babiloni et al. (2018b)	225	75	75	70.1 (6.1)	70.1 (6.9)	25.1 (2.6)	28.5 (0.9)	10.9 (4.3)	10.2 (4.3)	54.7	52.0	7
Babiloni et al. (2019)	83	30	30	74.2 (3.3)	74.7 (4.4)	25.6 (2.2)	28.5 (1.1)	9.8 (4.4)	9.7 (3.8)	46.7	40.0	8
Bagattini et al. (2022)	43	15	14	74.5 (6.05)	70.9 (3.9)	26.2 (1.8)	27.8 (2.1)	8.4 (3.1)	9.2 (3.7)	40.0	64.3	9
Barzegaran et al. (2016)	43	15	28	NA	NA	NA	NA	NA	NA	NA	NA	10
Cantero et al. (2009a)	40	ε4-: 11; ε4+: 9	20	68.4 (6.1)	66.8(4.7)	26.6 (2.7)	28.4 (1.4)	11.8 (6.5)	13.5 (5.6)	45.0	50.0	16
Cantero et al. (2009b)	40	20	20	68.4 (6.1)	66.8 (4.7)	26.6 (2.7)	28.4 (1.4)	11.8 (6.5)	13.5 (5.6)	45.0	50.0	17
Cecchetti et al. (2021)	129	Bio: 22; Non: 15	53	Bio: 70.5 (5.7); Non: 67.8 (6.3)	67.2 (6.3)	Bio: 25.9 (2.3); Non: 27.1 (2.7)	29.1 (1.4)	Bio: 9.9 (3.9); Non: 10.6 (4.7)	12.5 (5.0)	Bio: 63.6; Non: 26.7	54.7	19
Choi et al. (2021)	959	34	35	74.6 (7.0)	71.7 (5.2)	NA	NA	8.3 (4.7)	10.3 (5.2)	61.8	68.6	21
Crook-Rumsey et al. (2022)	96	27	39	77.5 (6.5)	72.9 (4.2)	NA	NA	NA	NA	44.4	61.5	22
Das & Puthankattil (2020)	33	13	20	67.8 (6.1)	60.2 (6.2)	23.9 (4.2)	29.3 (1.0)	11.1 (3.3)	12.3 (3.5)	46.2	50.0	23
Dattola et al. (2021)	40	21	10	NA	NA	NA	NA	NA	NA	NA	NA	24
Ding et al. (2022)	301	116	113	68.2 (10.8)	67.8 (9.9)	24.4 (3.4)	27.7 (1.8)	9.4 (4.6)	10.1 (3.5)	61.2	46.0	25

Duan et al. (2020)	60	22	38	NA	NA	NA	NA	NA	NA	NA	NA	26
Fide et al. (2023)	154	43	68	73.5 (5.9)	72.1 (6.2)	26.6 (4.3)	28.5 (2.7)	10.1 (4.2)	10.6 (5.1)	37.2	57.4	31
Franciotti et al. (2019)	125	42	42	74.8 (7.8)	73.7 (7.4)	25.0 (1.0)	28.9 (0.9)	9.2 (4.5)	9.9 (3.3)	62.0	50.0	32
Frantzidis et al. (2014)	64	17	23	68.6 (2.7)	68.0 (5.5)	25.6 (2.2)	28.0 (2.1)	NA	NA	76.5	73.9	35
Gómez et al. (2018)	78	41	37	76.1 (6.8)	76.3 (3.8)	27.2 (1.7)	28.8 (1.1)	Pn: 29; Sn:12	Pn: 11; Sn: 26	58.5	67.6	36
Gonzalez-Escamilla et al. (2014)	55	Total: 29; ε4-: 15; ε4+: 14	26	All: 70.2 (6.8); ε4-: 68.6 (7.1); ε4+: 71.8 (6.2)	66.5 (4.9)	All: 26.8 (2.6); ε4-: 27.5 (2.6); ε4+: 26.0 (2.5)	28.3 (1.3)	All: 8.2 (5.4); ε4-: 7.7 (5.2); ε4+: 8.8 (5.7)	8.6 (4.3)	All: 34.5; ε4-: 20.0; ε4+: 50.0	57.7	37
Gonzalez-Escamilla et al. (2015)	55	Total: 29; ε4-: 15; ε4+: 14	26	All: 70.2 (6.8); ε4-: 68.6 (7.1); ε4+: 71.8 (6.2)	66.5 (4.9)	All: 26.8 (2.6); ε4-: 27.5 (2.6); ε4+: 26.0 (2.5)	28.3 (1.3)	All: 8.2 (5.4); ε4-: 7.7 (5.2); ε4+: 8.8 (5.7)	8.6 (4.3)	All: 34.5; ε4-: 20.0; ε4+: 50.0	57.7	38
Guo et al (2021)	34	Mild:12; Mod: 11	11	Mild: 65.8 (8.4); Mod: 70.2 (5.8)	63.7 (2.0)	NA	NA	Mild: 11.1 (5.2); Mod: 11.7 (3.3)	10.0 (5.7)	Mild: 58.3; Mod: 63.6	75.0	40
Gurja et al. (2022)	67	23	22	65.4 (8.1)	63.3 (6.1)	28.1 (2.9)	29.6 (0.9)	15.4 (3.6)	14.8 (2.8)	26.1	45.5	41
Handayani et al. (2018)	22	10	12	72.2 (7.3)	70.2 (4.4)	20.3 (0.8)	27.3 (2.0)	NA	NA	70.0	66.7	43
Ioulietta et al. (2020)	92	30	22	70.4 (6.0)	67.2 (4.0)	27.1 (2.6)	29.1 (1.0)	11.5 (4.1)	13.2 (4.6)	73.3	63.3	48
Jiang (2005)	69	35	34	62.3 (6.5)	57.4 (4.0)	26.6 (2.0)	29.1 (1.3)	NA	NA	51.4	50.0	53
Jiang et al. (2006)	69	35	34	62.3 (6.5)	57.4 (4.0)	26.6 (2.0)	29.1 (1.3)	NA	NA	51.4	50.0	54
Jiang et al. (2008)	69	35	34	62.3 (6.5)	57.4 (4.0)	26.6 (2.0)	29.1 (1.3)	NA	NA	51.4	50.0	55
Josefsson et al. (2019)	27	13	14	73.1 (9.0)	67.2 (10.1)	26.5 (2.5)	29.5 (0.5)	14.1 (4.4)	16.5 (2.0)	NA	NA	56

Koenig et al. (2005)	A: 264, B: 155	A: 51, B: 41	A: 25, B: 21	A: 70.7 (NA), B: 61 (NA)	A: 67.1(NA), B: 63.4(NA)	NA	NA	NA	NA	NA	NA	61
La Foresta et al. (2019)	40	21	10	NA	NA	NA	NA	NA	NA	NA	NA	62
Lazarou et al. (2022)	73	23	21	69.6 (6.9)	62.6 (13.9)	26.2 (2.1)	29.3 (1.4)	10.1 (4.4)	14.7 (5.3)	69.6	61.9	63
Li et al. (2021)	49	28	21	65.2 (5.4)	67.1 (6.3)	26.6 (1.5)	29.2 (1.2)	14.9 (2.5)	15.2 (2.7)	46.5	57.1	67
Liu et al. (2012)	47	16	15	79.3 (8.5)	70.1 (8.5)	25.4 (2.1)	29.2 (0.7)	NA	NA	37.5	33.3	68
Mammone et al. (2019)	44	16	12	69 (8)	62 (4)	NA	NA	NA	NA	68.8	58.3	70
Michels et al. (2017)	34	17	17	72.1 (4.6)	71.8 (4.6)	28.4 (0.5)	29.7 (0.6)	14.8 (2.9)	15.4 (2.7)	27.8	27.8	72
Miraglia et al. (2016)	90	30	30	70.7 (6.9)	65.4 (9.6)	26.8 (1.6)	28.9 (0.9)	8.0 (4.7)	9.5 (3.8)	56.7	46.7	73
Miraglia et al. (2023)	33	22	11	74.2 (7.0)	65.4 (9.6)	27.6 (1.9)	29.0 (1.3)	15.1 (3.8)	14.5 (3.7)	NA	NA	74
Mohaved et al. (2022)	34	18	16	>55	>55	NA	NA	NA	NA	NA	NA	75
Musaeus et al. (2019a)	77	Total: 25; sMCI: 13; pMCI: 11	37	All: 71.4 (6.0); sMCI 72.4 (6.1); pMCI: 70.3 (6.6)	65.7 (6.9)	All: 27.6 (1.5); sMCI: 27.9 (1.4); pMCI: 27.1 (1.6)	29.1 (1.0)	All: 10.6 (3.4); sMCI: 10.7 (3.8); pMCI:10.6 (3.4)	12.7 (3.6)	All: 24.0; sMCI: 30.8; pMCI: 18.2	46.0	76
Musaeus et al. (2019b)	366	117	135	70.2 (8.1)	66.4 (7.6)	27.1 (2.2)	28.9 (1.3)	11.6 (3.9)	13.9 (3.6)	53.0	60.7	77
Núñez et al. (2019)	132	51	36	77.2 (NA)	75.9 (NA)	27 (NA)	29 (NA)	Pn: 35; Sn: 16	Pn:11; Sn: 25	58.8	69.4	78
Núñez et al. (2021)	160	67	43	NA	NA	NA	NA	Pn: 41; Sn: 26	Pn: 16; Sn: 27	56.7	69.8	79
Pons et al. (2010)	90	30	30	66.8 (4.7)	68.4 (6.1)	NA	NA	NA	NA	30.0	33.3	82
Požar et al. (2020)	40	13	27	73.6 (5.9)	73.1 (6.9)	NA	NA	14.2 (2.2)	15.1 (2.3)	84.6	92.6	83

Rodinskaia, et al. (2022)	30	10	10	80.1 (1.4)	78.4 (5.1)	NA	NA	11.8 (1.8)	12.2 (2.2)	50.0	60.0	84
Ruiz-Gomez et al. (2019b)	102	51	51	85.5 (7.3)	80.1 (7.1)	23.3 (2.8)	28.8 (1.1)	NA	NA	70.6	49.0	86
Ruiz-Gomez et al. (2021)	102	51	51	85.5 (7.3)	80.1 (7.1)	23.3 (2.8)	28.8 (1.1)	NA	NA	70.6	49.0	87
Sedghizadeh et al. (2022)	35	7	15	66.6 (6.9)	69.3 (6.7)	23.7 (2.9)	25.7 (3.2)	5.9 (6.8)	4.9 (4.7)	51.1	53.3	91
Spyrou et al. (2018)	27	13	14	NA	NA	NA	NA	NA	NA	NA	NA	94
Su et al. (2021)	49	28	21	65.0 (60-70	63.4 (60-70)	NA	NA	12.6 (2.3)	12.5 (3.1)	42.9	52.4	95
Sweeney-Reed et al. (2012)	22	11	11	71.6 (3.9)	68.2 (7.1)	26.9 (0.9)	28.8 (0.8)	14.9 (5.5)	13.5 (2.6)	NA	NA	96
Tao et al. (2006)	40	18	10	70.3 (13.3)	67.9 (9.6)	NA	NA	NA	NA	55.6	50.0	99
Teipel et al. (2009)	36	16	20	73.6 (7.8)	67.0 (7.3)	26.5 (1.1)	29.0 (0.7)	NA	NA	43.8	45.0	100
Timothy et al. (2017)	36	18	18	67.1 (7.2)	65.1 (5.6)	26.2 (1.4)	30	NA	NA	38.9	22.2	101
Tóth et al. (2014)	23	9	14	67.5 (3.2)	64.8 (2.5)	27.4 (1.8)	NA	NA	NA	66.7	57.1	102
Vanneste et al. (2021)	40	20	20	55-70	55-70	<25	>27	>4	>4	65.0	70.0	104
Vecchio et al. (2014)	378	154	50	71.5 (7.5)	72.9 (7.1)	26.1 (2.2)	28.4 (1.6)	8.1 (4.5)	8.9 (4.8)	55.8	50.0	105
Vecchio et al. (2016)	144	50	24	73.5 (10.6)	72.9 (7.8)	25.2 (3.5)	29.8 (1.0)	NA	NA	NA	NA	106
Vecchio et al. (2018)	86	47	22	72.8 (5.5)	68.3 (4.7)	25.9 (2.3)	28.6 (5.6)	10.5 (4.1)	13.7 (4.7)	NA	NA	108
Vecchio et al. (2021)	274	80	54	70.8 (11.3)	69.4 (12.9)	26.8 (2.7)	28.9 (1.3)	8.0 (7.7)	9.5 (5.1)	55.0	50.0	109
Wei et al. (2015)	27	10	17	75.2 (7.7)	74.8 (5.5)	NA	NA	7.0 (3.9)	7.6 (3.9)	40.0	41.2	115
Wen et al. (2014)	24	12	12	75.2 (7.1)	73.0 (6.5)	23.8 (5.2)	28.3 (1.8)	6.1 (4.2)	8.6 (4.3)	50.0	58.3	116

Xu et al. (2014)	25	11	14	75.5 (7.4)	74.0 (6.9)	24.0 (5.4)	26.8 (3.7)	7.1 (4.3)	7.5 (3.9)	45.5	57.1	117
Yan et al. (2021)	78	26	26	69.3 (8.1)	67.9 (7.2)	27.0 (1.5)	29.6 (0.6)	NA	NA	53.9	65.4	118
Youssef et al. (2021)	94	43	51	69.5 (9.0)	69.0 (7.4)	23.9 (3.3)	27.5 (1.7)	10.3 (3.2)	11.1 (2.8)	60.5	49.0	119
Zhang et al. (2022)	31	15	16	66.4 (6.1)	63.8 (2.5)	NA	NA	13.1 (3.0)	12.1 (2.9)	40.0	56.3	122
Zheng et al. (2007)	69	35	34	62.3 (6.5)	57.4 (4.0)	26.6 (2.0)	29.4 (0.9)	NA	NA	51.4	50.0	124

Notes: Studies with both MCI and AD samples are included in both tables; ^portion of sample with EEG data; Bio = AD-biomarker positive; Edu = education; ε4- = Apolipoprotein-E (APOE) ε4 non-carrier; ε4+ = APOE ε4 carrier; F = female; HC = healthy controls; MMSE = Mini-mental State Exam; Mod = moderate; NA = not available/not specified; Non = AD-biomarker negative; Pn = N with primary school only; pMCI = progressing MCI; Ref = reference number; sMCI = stable MCI; Sn = N with secondary school or higher.

Table S2.
Demographic data (mean(SD)) for studies comparing Alzheimer's disease (AD) and healthy control (HC) groups.

Authors (Year)	Total N^	AD N	HC N	AD Age	HC Age	AD MMSE	HC MMSE	AD EDU	HC EDU	AD %F	HC %F	Ref
Afshari & Jalili (2016)	51	25	26	NA	NA	NA	NA	NA	NA	36.0	42.3	1
Al-Nuaimi et al. (2021)	A:11; B:41; C:20; D:40	A:3; B:17; C:10; D:20	A:8; B:24; C:10; D:20	A: >65; B: 77.6 (10.0); C: 78.3 (4.0); D: 77.8 (5.5)	A: >65; B: 69.4 (11.5); C: 78.0 (4.2); D: 75.8 (3.9)	A: NA; B: NA; C: 22.8 (2.4); D: 21.2 (3.1)	A: NA; B: NA; C: 29.2 (0.6); D: 28.4 (1.0)	NA	NA	A: NA; B: 47.1; C: 50.0; D: 50.0	A: NA; B: 58.3; C: 50.0; D: 50.0	2
Babiloni et al. (2009a)	206	73	64	74.1 (1.0)	73.7 (0.9)	20.9 (0.5)	28.6 (0.2)	9.8 (0.6)	9.7 (0.6)	47.3	50.0	3
Babiloni et al. (2009b)	132	33	47	70.9 (1.7)	74.7 (0.9)	20.8 (0.7)	27.2 (0.4)	6.3 (0.6)	9.4 (0.7)	75.8	46.8	4
Babiloni et al. (2016)	220	120	100	69.8 (7.7)	69.0 (9.0)	19.0 (3.3)	28.8 (1.0)	9.2 (4.4)	9.7 (4.0)	65.0	62.0	5
Babiloni et al. (2018a)	158	42	40	73.3 (6.5)	72.9 (7.0)	18.9 (3.9)	28.7 (1.3)	8.1 (5.2)	8.5 (3.8)	59.5	60.0	6
Bagattini et al. (2022)	43	14	14	74.8 (6.3)	70.9 (3.9)	21.8 (1.4)	27.8 (2.1)	7.2 (2.7)	9.2 (3.7)	50.0	64.3	9
Barzegaran et al. (2016)	42	14	28	NA	NA	NA	NA	NA	NA	NA	NA	10
Birba et al. (2022)	63	15	21	73.5 (5.7)	73.5 (4.8)	NA	NA	12.3 (4.7)	13.5 (3.5)	66.7	81.0	11
Blinowska et al. (2017)	84	42	42	70.6 (8.5)	65.9 (8.5)	19.3 (5.2)	28.8 (1.5)	6.3 (3.0)	12.0 (4.6)	66.7	50.0	12
Bonanni et al. (2021)†	56	18	20	74.0 (7.0)	73.0 (7.0)	22.0 (1.0)	29.0 (1.0)	10.0 (5.0)	10.0 (4.0)	55.6	55.0	13
Cai et al. (2018)	40	20	20	74-78	70-76	12-15	28-30	NA	NA	60.0	50.0	14
Cai et al. (2020)	40	20	20	74-78	70-76	12-15	28-30	NA	NA	60.0	50.0	15

Canuet et al. (2012)	185	Total: 125; Mild: 38; Mod: 70; Sev: 17	60	All: 71.9 (7.7); Mild: 70.9 (5.9); Mod: 72.7 (8.1); Sev: 70.6 (9.5)	67.3 (6.8)	All: 15.2 (5.7); Mild: 21.8 (1.6); Mod: 13.6 (2.9); Sev: 5.9 (2.7)	28.8 (0.9)	NA	NA	65.6	40.0	18
Cecchetti et al. (2021)	129	39	53	68.7 (6.8)	67.2 (6.3)	19.5 (5.2)	29.1 (1.4)	9.0 (4.8)	12.5 (5.0)	53.9	54.7	19
Chan et al. (2013)	31	16	15	66.8 (4.7)	67.3 (5.8)	18.7 (5.0)	27.9 (2.5)	4.8 (3.5)	8.5 (4.9)	68.8	60.0	20
Choi et al. (2021)	959	29	27	76.2 (5.1)	73.8 (4.2)	NA	NA	5.7 (4.0)	8.0 (5.4)	82.8	81.5	21
Dattola et al. (2021)	40	9	10	NA	NA	NA	NA	NA	NA	NA	NA	24
Ding et al. (2022)	301	72	113	73.4 (8.8)	67.8 (9.9)	17.7 (6.9)	27.7 (1.8)	9.1 (4.9)	10.1 (3.5)	59.7	46.0	25
Duan et al. (2020)	41	17	24	69.4 (11.5)	77.6 (10.0)	NA	NA	NA	NA	47.1	58.3	26
Dubovik et al. (2013)	30	15	15	83.9 (4.5)	76.7 (5.6)	20.6 (2.9)	28.4 (1.5)	NA	NA	80.0	73.3	27
Engels et al. (2015)	451	318	133	NA	67.8 (6.3)	NA	28.5 (1.3)	NA	5.4 (1.2)	48.4	44.4	28
Escudero et al. (2016)	23	12	1	72.8 (8.0)	72.8 (6.1)	13.3 (5.6)	30.0 (0)	NA	NA	58.3	36.4	29
Fide et al. (2022)	201	56	54	74.7 (5.3)	72.8 (4.6)	19.8 (4.9)	29.3 (0.8)	9.2 (4.5)	10.0 (4.6)	56.14‡	66.67‡	30
Fide et al. (2023)	154	43	68	74.6 (5.0)	72.1 (6.2)	20.8 (4.4)	28.5 (2.7)	9.9 (4.6)	10.6 (5.1)	44.2	57.4	31
Franciotti et al. (2019)	125	41	42	77.3 (6.1)	73.7 (7.4)	21.5 (1.1)	28.9 (0.9)	8.2 (4.4)	9.9 (3.3)	61.0	50.0	32
Franciotti et al. (2022)†	56	18	20	74 (7)	73 (7)	22 (1)	29 (1)	10 (5)	10 (4)	55.6	55.0	33
Frangopoulou et al. (2022)	40	20	20	60.0 (4.4)	61.0 (6.7)	NA	NA	NA	NA	40.0	60.0	34
Frantzidis et al. (2014)	64	24	23	72.3 (6.3)	68.0 (5.5)	NA	NA	22.3 (2.5)	28.0 (2.1)	70.8	73.9	35
Guntekin et al. (2008)	40	Ut: 10; Tx: 11	19	NA	72.1 (6.6)	20-24	28-30	Pn: 14; Sn: 7	Pn: 11; Sn: 8	NA	42.1	39

Gurja et al. (2022)	67	22	22	66.8 (9.1)	63.3 (6.1)	17.2 (3.7)	29.6 (0.9)	14.1 (3.2)	14.8 (2.8)	31.8	45.5	41
Han et al. (2017)	39	20	19	69.1 (8.8)	66.7 (6.7)	23.6 (2)	29.2 (0.8)	14.1 (2.0)	13.4 (3.2)	60.0	63.2	42
Hata et al. (2016)	58	28	30	74.2 (8.9)	70.6 (5.6)	18.7 (4.5)	29.2 (1)	NA	NA	78.6	46.7	44
Herzog et al. (2022)	77	31	46	75.7 (7.6)	71.0 (7.3)	NA	NA	9.9 (5.0)	14.7 (4.1)	54.8	63.0	45
Hidasi et al. (2007)	24	14	10	67.4 (NA)	67.2 (NA)	20.2 (NA)	29.8 (NA)	NA	NA	57.1	60.0	46
Ho et al. (2014)	46	16	15	80.0 (9.6)	72.0 (10.5)	NA	NA	NA	NA	NA	NA	47
Ioulietta et al. (2020)	92	20	22	73.2 (8.2)	67.2 (4.0)	22.3 (3.4)	29.1 (1.0)	9.8 (5.5)	13.2 (4.6)	60.0	63.6	48
Jalili et al. (2016)	30	16	14	69.1 (10.6)	68.0 (11.2)	21 (4.5)	29 (1)	NA	NA	NA	NA	49
Jalili (2017)	48	23	25	72.1 (10.1)	68.0 (11.2)	22.4 (4.3)	29.0 (1.0)	NA	NA	39.1	44.0	50
Jelic et al. (1997)	59	ε4-: 17; ε4 het: 14; ε4 hom: 10	18	ε4-: 61.8 (8.6); ε4 het: 61.2 (7.4); ε4 hom: 65.7 (5.7)	63.4 (11.0)	ε4-: 23.2 (3.7); ε4 het: 21.6 (4.0); ε4 hom: 22.7 (2.6)	28.9 (1.1)	ε4-: 10.4 (3.5); ε4 het: 9.7 (3.9); ε4 hom: 12.2 (4.5)	10.5 (3.6)	ε4-: 41.2; ε4 het: 57.1; ε4 hom: 50	50.0	51
Jeong et al. (2001)	30	15	15	70.4 (3.1)	69.7 (4.3)	9.4 (3.4)	27.3 (0.6)	NA	NA	53.3	53.3	52
Kabbara et al. (2018)	20	10	10	66-81	64-78	NA	NA	NA	NA	50.0	40.0	57
Kim et al. (2018)	20	10	10	73.5 (7.4)	72.9 (4.3)	19.3 (3.4)	26.7 (0.8)	NA	NA	30.0	40.0	58
Knyazeva et al. (2010)	34	17	16	69.4 (10.6)	67.6 (11.6)	21.8 (3.9)	28.5 (1.2)	11.6 (3.3)	13.1 (3.2)	35.3	64.7	59
Knyazeva et al. (2013)	30	Total: 15; FP: 8; SP:7	15	All: 68.7 (5.1); FP: 71.9 (4.0); SP: 65.9 (3.1)	67.6 (5.0)	All: 22.6 (1.1); FP: 20.5 (1.5); SP: 24.6 (1.4)	28.5 (1.2)	All: NA; FP: 2.3 (0.3); SP: 2.1 (0.3)	NA	All: 20; FP: 37.5; SP: 0.0	60.0	60

Koenig et al. (2005)	A: 264; B: 155	A: Mild: 71; Mod: 56; MS: 21; B: Mild: 45; Mod: 18	A: 25; B: 21	A: Mild: 70.9; Mod: 71.7; MS: 74.3; B: Mild: 62; Mod: 63	A: 67.1; B: 63.4	A: NA; B: Mild: >20 Mod: 15-20	NA	NA	NA	NA	NA	61
La Foresta et al. (2019)	40	9	10	NA	NA	NA	NA	NA	NA	NA	NA	62
Lazarou et al. (2022)	73	12	21	70.5 (6.8)	62.6 (13.9)	20.7 (5.5)	26.2 (2.1)	9.8 (4.0)	14.7 (5.3)	66.7	61.9	63
Lee et al. (2010)	47	25	22	73.8 (7.2)	72.8 (4.5)	18.2 (3.7)	26.1 (1.4)	5.1 (4.6)	5.8 (4.7)	80.0	40.9	64
Leuchter et al. (1994)	56	22	18	72.6 (7.2)	73.6 (7.6)	18.7 (NA)	28.7 (NA)	NA	NA	NA	NA	65
Li et al. (2019)	14	6	8	72.5 (7.3)	62.8 (8.2)	19.7 (3.0)	28.1 (1.1)	11.2 (2.8)	11.0 (2.5)	66.7	25.0	66
Locatelli et al. (1998)	20	10	10	67.3 (NA)	NA	19-23	NA	NA	NA	NA	NA	69
Mammone et al. (2019)	44	16	12	74 (10)	62 (4)	NA	NA	NA	NA	56.3	58.3	70
Mehraram et al. (2020)	96	32	18	76.6 (7.7)	76.2 (5.5)	20.2 (4.3)	29.2 (0.9)	NA	NA	31.3	38.9	71
Miraglia et al. (2016)	90	30	30	72.0 (8.1)	65.4 (9.6)	22.3 (5.3)	28.9 (0.9)	8.2 (3.9)	9.5 (3.8)	66.7	46.7	73
Musaeus et al. (2019a)	77	15	37	70.1 (7.8)	65.7 (6.9)	26.3 (3.2)	29.1 (1.0)	12.1 (4.0)	12.7 (3.6)	53.3	46.0	76
Musaeus et al. (2019b)	366	114	135	75.5 (7.7)	66.4 (7.6)	23.5 (3.8)	28.9 (1.3)	10.1 (3.4)	13.9 (3.6)	60.7	60.7	77
Núñez et al. (2019)	132	45	36	79.5 (NA)	75.9 (NA)	22 (NA)	29 (NA)	Pn: 31; Sn: 14	Pn: 11; Sn: 25	55.6	69.4	78
Núñez et al. (2021)	160	50	43	NA	NA	NA	NA	Pn: 35, Sn: 15	Pn: 16, Sn: 27	54.0	69.8	79
Park et al. (2008)	45	22	23	73.8 (7.7)	72.0 (4.8)	17.9 (3.8)	26.4 (1.0)	5.2 (4.7)	6.2 (4.9)	86.4	39.1	80
Peraza et al. (2018)	89	26	17	76.5 (7.8)	76.2 (5.7)	20.1 (4.3)	29.1 (0.9)	NA	NA	30.8	41.2	81
Rodinskaia, et al. (2022)	30	10	10	79.7 (4.3)	78.4 (5.1)	NA	NA	13.4 (2.3)	12.2 (2.2)	60.0	60.0	84

Ruiz-Gomez et al. (2019a)	109	72	37	NA	NA	NA	NA	NA	NA	NA	NA	85
Ruiz-Gomez et al. (2019b)	202	Total: 151; Mild: 51; Mod: 50; Sev: 50	51	All: NA; Mild: 80.7(7.1); Mod: 81.3 (8.0); Sev: 80.0 (7.8)	80.1 (7.1)	All: NA; Mild: 22.5 (2.3); Mod: 13.6 (2.8); Sev: 2.4 (3.7)	28.8 (1.1)	NA	NA	All: 73.2; Mild: 58.8; Mod: 86.0; Sev: 86.0	50.0	86
Ruiz-Gomez et al. (2021)	202	Total: 151; Mild: 51; Mod: 50; Sev: 50	51	All: NA; Mild: 80.7 (7.1); Mod: 81.3 (8.0); Sev: 80.0 (7.8)	80.1 (7.1)	All: NA; Mild: 22.5 (2.3); Mod: 13.6 (2.8); Sev: 2.4 (3.7)	28.8 (1.1)	NA	NA	All: 73.2; Mild: 58.8; Mod: 86.0; Sev: 86.0	50.0	87
Sankari et al. (2011)	27	20	7	74	71	NA	NA	NA	NA	NA	NA	88
Sankari et al. (2012)	27	20	7	74	71	NA	NA	NA	NA	NA	NA	89
Sedghizadeh et al. (2020)	24	11	15	76.6 (9.2)	68.2 (6.2)	15.7 (2.9)	25.8 (3.3)	4.2 (4.7)	3.4 (2.9)	64.0	46.0	90
Sedghizadeh et al. (2022)	35	13	15	75.3 (9.9)	69.3 (6.7)	16.5 (3.2)	25.7 (3.2)	3.3 (3.0)	4.9 (4.7)	61.5	53.3	91
Smith et al. (2016)	23	12	1	72.8 (8.0)	72.8 (6.1)	13.3 (5.6)	30.0 (0.0)	NA	NA	58.3	36.4	92
Song et al. (2018)	30	15	15	77.6 (3.4)	72.2 (1.9)	21.3 (5.8)	27.1 (1.3)	>6	>6	53.3	60.0	93
Tahaei et al. (2012)	34	17	17	69.4 (10.6)	67.6 (11.6)	21.8 (3.9)	28.5 (1.2)	11.6 (3.3)	13.1 (3.2)	35.3	64.7	97
Tait et al. (2019)	47	21	26	79.0 (9.0)	76.0 (7.0)	23.0 (3.0)	29.0 (1.0)	NA	NA	61.9	46.2	98
Tao et al. (2006)	40	12	10	69.4 (9.0)	67.9 (9.6)	NA	NA	NA	NA	58.3	50.0	99
Tyrer et al. (2020)	42	21	21	80.5 (6.2)	73.7 (6.4)	22.6 (4.5)	29.8 (0.4)	13.2 (1.9)	16.1 (2.6)	52.4	57.1	103
Vecchio et al. (2014)	378	174	50	73.2 (7.4)	72.9 (7.1)	20.1 (4.2)	28.4 (1.6)	7.1 (4.2)	8.9 (4.8)	71.3	50.0	105
Vecchio et al. (2016)	144	70	24	73.2 (17.6)	72.9 (7.8)	21.4 (5.0)	29.8 (1.0)	NA	NA	NA	NA	106

Vecchio et al. (2017)	144	110	34	73.2 (5.2)	73.8 (4.1)	21.3 (3.2)	29.8 (1.8)	7.0 (4.2)	8.0 (5.3)	72.7	58.8	107
Vecchio et al. (2018)	86	17	22	70.1 (6.2)	68.3 (4.7)	20.1 (2.1)	28.6 (NA)	10.4 (4.5)	13.7 (4.7)	NA	NA	108
Vecchio et al. (2021)	274	100	54	72.0 (15.6)	69.4 (12.9)	21.3 (NA)	28.9 (1.3)	8.2	9.5	60.0	50.0	109
Vecchio et al. (2022)	80	12	32	71.0 (5.9)	70.0 (5.1)	20.3 (3.4)	NA	NA	NA	58.3	65.6	110
Vysata et al. (2015)	240	120	120	71.5 (6.8)	69.1 (5.7)	15.8 (1.7)	NA	NA	NA	56.7	55.0	111
Wang et al. (2014)	28	14	14	74-78	70-76	11.7-14.9	28.1-30	NA	NA	57.1	71.4	112
Wang et al. (2015)	28	14	14	74-78	70-76	11.7-14.9	28.1-30	NA	NA	57.1	71.4	113
Wang et al. (2022)	61	19	21	68.7 (5.6)	63.9 (6.8)	24.3 (5.8)	28.0 (1.6)	10.7 (3.4)	12.1 (3.6)	68.4	76.2	114
Yan et al. (2021)	78	26	26	69.4 (8.8)	67.9 (7.2)	14.2 (5.3)	29.6 (0.6)	NA	NA	69.2	65.4	118
Yu et al. (2018)	28	14	14	74-78	70-76	12-15	28-30	NA	NA	57.1	71.4	120
Yu et al. (2020)	60	30	30	74-78	70-76	12-15	28-30	NA	NA	NA	NA	121
Zhao et al. (2019)	40	20	20	64.0 (8.3)	67.4 (11.8)	20.1 (4.0)	28.2 (1.4)	11.8 (2.1)	15.2 (3.0)	50.0	55.0	123

Notes: Studies with both MCI and AD samples are included in both tables; ^portion of sample with EEG data; † Time2 (disease onset) shown; ‡ approximate due to inconsistent reporting in paper; Edu = education; ε4- = Apolipoprotein-E (APOE) ε4 non-carrier; ε4+ = APOE ε4 carrier; F = female; FP = fast-progressing; HC = healthy controls; het = heterozygous; hom = homozygous; MMSE = Mini-mental State Exam; Mod = moderate; MS = moderately severe; NA = not available/not specified; Pn = N with primary school only; Ref = reference number; Sev = severe; Sn = N with secondary school or higher; SP = slow progressing; Tx = treated; Ut = untreated.

Table S3.
Detailed methods for studies included in the review.

1. Coherence Methods

Authors (Year)	Connectivity Method or Type	Frequencies Analyzed (Hz)	Rest Type, Duration	Active Task Type	Sensor-Level Approach	Source Space Approach	Segment duration (ms)	Clinical group(s)	Ref
Al-Nuaimi et al. (2021)	Magnitude squared	Delta (0-4), theta (4-8), alpha (8-12), beta (12-30), gamma (30-45), ratios between each band	NA/NA		Pairs		NA	AD	2
Babiloni et al. (2009b)	Magnitude squared	Delta (2-4), theta (4-8), alpha1 (8-10), alpha2 (10-13), beta1 (13-20), beta2 (20-30)	EC, 5min		Pairs		2,000	MCI, AD	4
Babiloni et al. (2016)	Lagged linear connectivity	Delta (2-4), theta (4-8), alpha1 (8-10.5), alpha2 (10.5-13), beta1 (13-20), beta2 (20-30), gamma (30-40)	EC, ≅5min			eLORETA	2,000	AD	5
Babiloni et al. (2018a)	Lagged linear connectivity	*Delta, theta, alpha1, alpha2, alpha3, beta1 (14-20), beta2 (20-30), gamma (30-40)	EC, ≅5min			eLORETA	2,000	AD	6
Babiloni et al. (2018b)	Lagged linear connectivity	*Delta, theta, alpha1, alpha2, alpha3, beta1, beta2, gamma	EC,5min			eLORETA	2,000	MCI	7
Babiloni et al. (2019)	Lagged linear connectivity	*Delta, theta, alpha1, alpha2, alpha3, beta1 (14-20), beta2 (20-30), gamma (30-40)	EC, ≅5min			eLORETA	2,000	AD	8
Barzegaran et al. (2016)	Lagged linear connectivity	Theta (3-8), alpha (8-14), beta1 (14-25)	EC, NA; EO, NA‡			LAURA	1,000	MCI, AD	10
Blinowska et al. (2017)	Partial & ordinary	Delta (1-4), theta (4-8), alpha (8-13), beta (13-30), gamma (30-45)	EC, NA		Pairs		2,000	AD	12

Chan et al. (2013)	Magnitude squared	Delta (1-3), theta (4-7), alpha (8-12), beta (13-30)	EC, 2 min EO, 10 sec PS‡, 10 sec		Pairs		10,000	AD	20
Dubovik et al. (2013)	Imaginary	Theta (3-5), alpha (7-13)	EC, NA			Beamformer	1,000	AD	27
Fide et al. (2022)	Imaginary	Delta (0.5-3.5), theta (4-7.5), alpha (8-13), alpha1 (8-10), alpha2 (10.5-13), beta (13-30), beta1 (13-20), beta2 (20.5-30)	EC, 4min		Avg regions		8,000	AD	30
Fide et al. (2023)	Imaginary	Delta (0.5-3.5), theta (4-7.5), alpha (8-13), beta (13.5-29.5), gamma (30-48)		Visual oddball^	Pairs		800	MCI, AD	31
Guntekin et al. (2008)	Magnitude squared	Delta (1-3.5), Theta (4-7), Alpha (8-13)		Visual oddball^	Pairs		650	AD	39
Handayani et al. (2018)	Magnitude squared	Delta (1-4), theta (4-7), alpha (7-13), beta (13-30)	EC, 20min		Pairs		NA	MCI	43
Hidasi et al. (2007)	NA	Delta (0.5-4), Theta (4-8), Alpha1 (8-11), Alpha2 (11-14), Beta1 (14-25), Beta 2 (25-35)	EC 2min†; EO 20sec†		Pairs		2,560	AD	46
Ho et al. (2014)	Magnitude squared	NA		Auditory oddball	CP3-F4		NA	AD	47
Jelic et al. (1997)	Magnitude squared	Alpha (8-13)	EC, NA		Avg regions		15,000	AD	51
Jiang (2005)	Magnitude squared	Delta (1-3.5), theta (4-7.5), alpha1 (8-10), alpha2 (10.5-13), beta1 (13.5-18), beta2 (18.5-30)	EC, 10min	WM	Pairs		2,000	MCI	53
Jiang et al. (2006)	Magnitude squared	Delta (1-3.5), theta (4-7.5), alpha1 (8-10), alpha2 (10.5-13), beta1 (13.5-18), beta2 (18.5-30)	EC, 10min	WM	Pairs		2,000	MCI	54

Jiang et al. (2008)	Magnitude squared	Delta (1-3.5), theta (4-7.5), alpha1 (8-10), alpha2 (10.5-13), beta1 (13.5-18), beta2 (18.5-30)	EC, 10min	WM	Pairs		2,000	MCI	55
Leuchter et al. (1994)	Magnitude squared	4, 12, 16	EC, 5min		Avg pairs		4,000	AD	65
Locatelli et al. (1998)	Magnitude squared	Delta (0.5-4), theta (4-8), alpha (8-12), beta (12-30)	EC, 20min		Avg pairs		1,000	AD	69
Michels et al. (2017)	Magnitude squared, Renormalized partial directed	Delta (1-3), theta (4-7), alpha (8-13), beta (14-30), gamma (30-49)	EC, 5min			Beamformer	2,000	MCI	72
Musaeus et al. (2019a)	Magnitude squared, Imaginary	Delta (1-3.99); theta (4-7.99); alpha (8-12.99); beta (13-29.99)	EC, NA		Pairs		1,000	MCI, AD	76
Musaeus et al. (2019b)	Magnitude squared, imaginary	Delta (1-3.99); theta (4-7.99); alpha (8-12.99); beta (13-29.99); beta1 (13-17.99); beta2 (18-23.99); beta3 (24-29.99)	EC, NA; EO, NA		Pairs		1,000	MCI, AD	77
Núñez et al. (2019)	Magnitude squared	Delta (1-4), theta (4-8), alpha (8-13), beta1 (13-19), beta2 (19-30)	EC, 5min		Avg regions		15,000	MCI, AD	78
Rodinskaia et al. (2022)	NA	Delta, theta, alpha, beta, gamma (NA)	EC, 1min	Multiple cognitive tasks	Pairs		1,200	MCI, AD	84
Ruiz-Gomez et al. (2019a)	Magnitude squared	Delta (1-4), theta (4-8), alpha (8-13), beta1 (13-19), beta2 (19-30), gamma (30-70)	EC, 5min		Pairs		5,000	AD	85
Ruiz-Gomez et al. (2019b)	Magnitude squared, imaginary, lagged	Delta (1-4), theta (4-8), alpha (8-13), beta1 (13-19), beta2 (19-30), gamma (30-70)	EC, 5min		Pairs		5,000	MCI, AD	86

Sankari et al. (2011)	Magnitude squared	Delta (0-4), theta (4-8), alpha (8-12), beta (12-30)	EC, NA		Pairs		8,000	AD	88
Sankari et al. (2012)	Magnitude squared, wavelet, wavelet fraction	Delta (0-4), theta (4-8), alpha (8-12), beta (12-30)	EC, NA		Pairs		8,000	AD	89
Sedghizadeh et al. (2020)	Imaginary	Delta (0.5-3.99), theta (4.0-7.99), alpha (8.0-12.99) beta (13.0-29.99), gamma (30.0-40.5)		Olfactory oddball~	Pairs		3,000	AD	90
Sedghizadeh et al. (2022)	Amplitude, phase-amplitude	A: low gamma (35-45); PA: theta (4-8), gamma (39-41)		Olfactory oddball	Fz, Cz, Pz		3,000	MCI, AD	91
Tao et al. (2006)	NA	Gamma (20-45)	EC, 5min	Counting	Pairs		24,560	MCI, AD	99
Teipel et al. (2009)	NA	Delta (1-3), theta (4-7), alpha (8-12), beta (13-32)	EC, 10min		Avg regions		2,000	MCI	100
Vanneste et al. (2021)	Lagged linear connectivity	Delta (2-3.5), theta (4-7.5), alpha (8-12), beta (13-30), gamma (30.5-44.5)	EC, 5min			eLORETA	NA	MCI	104
Vysata et al. (2015)	Wavelet	Five wavelet scales (NA)	EC, 5-8 min		Pairs		60-80,000	AD	111
Wang et al. (2014)	Magnitude squared	Delta (0.5-4), theta (4-7), alpha1 (8-10), alpha2 (10-12), beta (13-30), gamma (30-40)	EC, 10min		Pairs		8,000	AD	112
Wang et al. (2015)	Magnitude squared	Alpha2 (10-12)	EC, 10min		Pairs		8,000	AD	113
Xu et al. (2014)	Magnitude squared	Theta (4-8), alpha (8-13), beta (13-30), gamma (30-40), full band (4-60)	EC, ≅20min		Pairs		2,000	MCI	117
Zheng et al. (2007)	Magnitude squared	Alpha1 (8-10), alpha2 (10.5-13)	EC, 10min	WM	Pairs		2,000	MCI	124

2. Phase-locked Methods

Authors (Year)	Connectivity Method or Type	Frequencies Analyzed (Hz)	Rest Type, Duration	Active Task Type	Sensor-Level Approach	Source Space Approach	Segment Duration (ms)	Clinical Groups	Ref.
Cai et al. (2018)	Phase synchrony index	Delta (0-3.75), theta (3.75-7.5), alpha (7.5-15), beta (15-30)	EC, 32sec		Pairs		8,000	AD	14
Cantero et al. (2009a)	Phase synchronization	Alpha1 (7.5-10.0), alpha2 (10.1-12.5)	EC, NA			swLORETA	NA	MCI	17
Canuet et al. (2012)	Lagged phase synchronization	Delta (1.5-4), theta (4-8), alpha1 (8-10), alpha2 (10-13), beta1 (13-20), beta2 (21-30)	EC, 3min			eLORETA	2,000	AD	18
Choi et al. (2021)	Phase locking value	Theta (4-8), alpha1 (8-10), alpha2 (10-12), beta1 (12-18), beta2 (18-30)	EC, 4min			WMNE	2,000	MCI, AD	21
Das & Puthankattil (2020)	Weighted phase lag index	Delta (0.5-4), theta (4-8), alpha1 (8-10), alpha2 (10-13), beta (13-30), gamma (30-48)	EC, 5min†, EO 5min†	Mental arithmetic	Avg regions		5,000	MCI	23
Engels et al. (2015)	Phase lag index	Delta (0.5-4), theta (4-8), alpha1 (8-10), alpha2 (10-13), beta (13-30)	EC, NA		Avg regions		~8,000	AD	28
Frangopoulou et al. (2022)	Phase locking value	Delta (1-4), theta (4-8), alpha (8-13), beta (13-30), gamma (36-44)	EO, 12sec		Pairs		12,000	AD	34
Gómez et al. (2018)	Phase slope index	Delta (1-4), theta (4-8), alpha (8-13), beta1 (13-19), beta2 (19-30), gamma (30-70)	EC, NA		Pairs		5,000	MCI	36
Gonzalez-Escamilla et al. (2014)	Phase lag index	Alpha (7.5-12.5)	EC, 10min		Avg regions		8,000	MCI	37
Gonzalez-Escamilla et al. (2015)	Phase lag index	Indiv. alpha peak	EC, 56sec		Pairs		8,000	MCI	38

Gurja et al. (2022)	Lagged phase synchronization	Delta (2-4), theta (4-8), alpha1 (8-10.5), alpha2 (10.5-13), beta1 (13-20), beta2 (20-30), gamma (30-45)	EC, ≥5min			eLORETA	1,000	MCI, AD	41
Han et al. (2017)	Phase lag index	Delta (1-4), theta (4-8), alpha (8-13), beta (13-30), gamma (30-45)		Object location, memory	Avg regions		1,000	AD	42
Handayani et al. (2018)	Phase locking value	Delta (1-4), theta (4-7), alpha (7-13), beta (13-30)	EC, 20min		Pairs		NA	MCI	43
Hata et al. (2016)	Lagged phase synchronization	Delta (2-4), theta (4-8), alpha1 (8-10), alpha2 (10-13), beta1 (13-20), and beta2 (20-30)	EC, EO, 5min (HC) or 10 min (AD) total			eLORETA	2,000	AD	44
Kabbara et al. (2018)	Phase locking value	Theta (4-8); alpha1 (8-10); alpha2 (10-13); beta (13-30)	EC, 10min			WMNE	40,000	AD	57
Knyazeva et al. (2010)	Multivariate phase synchronization	Delta (1-3), theta (3-7), alpha1 (7-9.5), alpha2 (9.5-13), beta1 (13-20), beta2 (20-30)	EC, 3-4min		NA		1,000	AD	59
Li et al. (2019)	Weighted phase lag index	Alpha1 (8-10), alpha2 (10-13), beta (13-30)		WM		DBTN	17,000	AD	66
Li et al. (2021)	Phase synchronization index	Theta (range NA)	EC, 5min		NA		1,000	MCI	67
Mehraram et al. (2020)	Weighted phase lag index	Theta (4-7.5), alpha (8-13.5), beta (14-20.5)	EC, 2.5min		Avg regions		2,000	AD	71
Musaeus et al. (2019b)	Weighted phase lag index	Delta (1-3.99); theta (4-7.99); alpha (8-12.99); beta (13-29.99); beta1 (13-17.99); beta2 (18-23.99); beta3 (24-29.99)	EC, NA; EO, NA		Pairs		1,000	MCI, AD	77
Núñez et al. (2019)	Phase lag index	Delta (1-4), theta (4-8), alpha (8-13), beta1 (13-19), beta2 (19-30)	EC, 5min		Avg regions		15,000	MCI, AD	78

Pons et al. (2010)	Phase lag index	Alpha1 (7-9), alpha2 (9.1-11)	EC, 2.5min		Pairs		8,000	MCI	82
Požar et al. (2020)	Phase lag index	Delta (0.5-4), theta (4-8), alpha1 (8-10), alpha2 (10-13), beta (13-30)	EC, ≥3min		Avg regions		2,000	MCI	83
Ruiz-Gomez et al. (2019a)	Phase lag index	Delta (1-4), theta (4-8), alpha (8-13), beta1 (13-19), beta2 (19-30), gamma (30-70)	EC, 5min		Pairs		5,000	AD	85
Ruiz-Gomez et al. (2019b)	Phase lag index, phase locking value, corrected imaginary phase locking value	Delta (1-4), theta (4-8), alpha (8-13), beta1 (13-19), beta2 (19-30), gamma (30-70)	EC, 5min		Pairs		5,000	MCI, AD	86
Ruiz-Gomez et al. (2021)	Phase lag index	Delta (1-4), theta (4-8), alpha (8-13), beta1 (13-19), beta2 (19-30), gamma (30-70)	EC, 5min			sLORETA	5,000	MCI, AD	87
Spyrou et al. (2018)	Phase lag index	Theta (4-7), alpha (8-12), beta (13-20)		Visual memory	Pairs		1,000	MCI	94
Su et al. (2021)	Phase locking value	Delta (1-4), theta (4-8), alpha (8-13), beta (13-30), gamma (30-45)	EC, 5min		Pairs		NA	MCI	95
Sweeney-Reed et al. (2012)	Phase locking value (EMDPL)	4-12		DRM memory	Avg regions (frontal, parietal)		600	MCI	96
Tóth et al. (2014)	Phase lag index	Delta (0.5-4), theta (4-8), alpha1 (8-10), alpha2 (10-13), beta (13-30), gamma (30-45)	EC, 4min; EO, 4min§		Avg regions		2,048	MCI	102
Wang et al. (2022)	Lagged phase synchronization	Delta (2-3.5), theta (4-7.5), alpha1 (8.5-10), alpha2 (10.5-12), beta1 (12.5-18), beta2 (18.5-21), beta3 (21.5-30), gamma (30.5-45)	EC, 5min			eLORETA, sLORETA	2,000	AD	114
Yan et al. (2021)	Weighted phase lag index	Delta (2-4), theta (4-8), alpha1 (8-10.5), alpha2 (10.5-13), beta1 (13-20), beta2 (20-30)	EC, 10min		Pairs		2,000	MCI, AD	118

Youssef et al. (2021)	Debiased weighted phase lag index	Delta (1-4), theta (4-7), alpha1 (8-10), alpha2 (10-13), beta (13-30), gamma (30-45)	EC, 5min†		Avg regions		4,000	MCI	119
Yu et al. (2020)	Phase synchronization index	Broadband (0.5-30)	EC, EO, 10min total		Pairs		8,000	AD	121
Zhang et al. (2022)	Phase synchronization	Alpha (NA)	EC, 8min			DICOS	NA	MCI	122

3. Graph Theory Methods

Authors (Year)	Connectivity Method or Type	Frequencies Analyzed (Hz)	Rest Type, Duration	Active Task Type	Sensor-Level Approach	Source Space Approach	Segment Duration (ms)	Clinical Groups	Ref.
Afshari & Jalili (2016)	Global efficiency, local efficiency, attack tolerance - via directed transfer function	Delta (0.5-3), theta (3-7), alpha (7-13), beta (13-30)	EC, 3-4min		NA		1,000	AD	1
Bagattini et al. (2022)	Divisibility index - via partial directed coherence	Theta (3-7)		Enumeration	Avg regions		400	MCI, AD	9
Cai et al. (2018)	Within-, between-frequency phase coupling - via phase synchrony index	Delta (0-3.75), theta (3.75-7.5), alpha (7.5-15), beta (15-30)	EC, 32sec		Global (16 channel x band nodes)		8,000	AD	14
Cai et al. (2020)	Multiplex clustering coefficient, multiplex participation coefficient, node degree layer proportion - via normalized imaginary phase locking value	Delta (1-4), theta (4-8), alpha (8-12), beta (12-30)	EC, ≥10min		NA		4,000	AD	15

Cecchetti et al. (2021)	Nodal strength, characteristic path length, local efficiency, clustering coefficient - via lagged linear connectivity	Theta (4.0-8.0), alpha 2 (10.0-12.0)	EC, ≥10min			eLORETA	2,000	MCI, AD	19
Choi et al. (2021)	Clustering coefficient - via phase locking value	Theta (4-8), alpha1 (8-10), alpha2 (10-12), beta1 (12-18), beta2 (18-30)	EC, 4min			WMNE	2,000	MCI, AD	21
Das & Puthankattil (2020)	Degree, leaf fraction, diameter, eccentricity, betweenness centrality, tree hierarchy - via weighted phase lag index	Delta (0.5-4), theta (4-8), alpha1 (8-10), alpha2 (10-13), beta (13-30), gamma (30-48)	EC, 5min†; EO 5min†	Mental arithmetic	Avg regions		5,000	MCI	23
Dattola et al. (2021)	Connection density index, Randic index, normalized Kirchoff index - via lagged linear connectivity	Broadband (1-40)	EC, 2min			eLORETA	120,000	MCI, AD	24
Duan et al. (2020)	Clustering coefficient, node strength, characteristic path length, betweenness centrality, resilience, global efficiency, versatility - via coherence and Pearson's correlation	Theta (4-8), alpha1 (8-10), alpha2 (10-13), beta (13-20)	EC, 5min		NA		20,000	MCI, AD	26
Engels et al. (2015)	Betweenness centrality - via phase lag index	Delta (0.5-4), theta (4-8), alpha1 (8-10), alpha2 (10-13), beta (13-30)	EC, NA		Avg regions		~8,000	AD	28

Escudero et al. (2016)	Mean local clustering coefficient, degree centrality, efficiency - via imaginary part of coherence	Broadband (0.5-40)	EC, ≥5min	NA	5,000	AD	29
Franciotti et al. (2019)	Degree, in-degree, out-degree, clustering coefficient, local efficiency, characteristic path length, global efficiency, assortativity - via granger causality	NA	EC, 2.5min	Pairs	16,000	MCI, AD	32
Franciotti et al. (2022)	Assortativity, global and local efficiency, clustering coefficient, small world propensity - via mutual information	Broadband (1-100)	EC, 10min	NA	2,000	AD	33
Frantzidis et al. (2014)	Small worldness, characteristic path length, clustering coefficient, global efficiency, normalized relative betweenness - via relative wavelet entropy	Broadband (NA)	EC, 5min	Pairs	20,000	MCI, AD	35
Ioulietta et al. (2020)	Clustering coefficient, strength, betweenness centrality - via Pearson correlation coefficient	Broadband (0.3-75)	EC, 2-3min; EO, 2-3min	NA	2,000	MCI, AD	48

Jalili et al. (2016)	Avg path length, clustering coefficient, betweenness centrality, local efficiency, global efficiency - via correlations, coherence, phase order, and synchronization likelihood	Alpha (7-13)	EC, 3-4min		NA		1,000	AD	49
Jalili (2017)	Local efficiency, clustering coefficient, global efficiency, edge and node betweenness centrality, modularity index - via Pearson correlation coefficient	Delta (1-3), theta (3-7), alpha (7-13), beta (13-30), gamma (30-50)	EC, EO 3-4 min total		Pairs		1,000	AD	50
Josefsson et al. (2019)	Clustering, characteristic path length, small worldness, eccentricity - via joint distribution entropy	Beta (13-30)		Memory	NA		1,200	MCI	56
Kabbara et al. (2018)	Avg clustering coefficient, global efficiency, recruitment, integration, hub identification, vulnerability - via phase locking value	Theta (4-8); alpha1 (8-10); alpha2 (10-13); beta (13-30)	EC, 10min			WMNE	40,000	AD	57
La Foresta et al. (2019)	Characteristic path length, clustering coefficient, connection density - via lagged linear connectivity	Broadband (1-40)	EC, 2min			eLORETA	3,000	MCI, AD	62

Lazarou et al. (2022)	Clustering coefficient, strength, betweenness centrality - via Pearson correlation coefficient	Broadband (0.3-70)		Verbal attention, memory	NA		1,000	MCI, AD	63
Li et al. (2019)	Degree, clustering coefficient, betweenness centrality - via weighted phase lag index	Alpha1 (8-10), alpha2 (10-13), beta (13-30)		WM		DBTN	17,000	AD	66
Li et al. (2021)	Clustering coefficient, avg node degree, global efficiency - via phase synchronization index and directed transfer function	Theta (NA)	EC, 5min		NA		1,000	MCI	67
Mammone et al. (2019)	Characteristic path length, clustering coefficient, global efficiency - via permutation disalignment index	Broadband (1-40)	EC, 2min		Pairs		1,000	MCI, AD	70
Mehraram et al. (2020)	Node degree, clustering coefficient, characteristic path length, small worldness, modularity - via weighted phase lag index	Theta (4-7.5), alpha (8-13.5), beta (14-20.5)	EC, 2.5min		Avg regions		2,000	AD	71
Miraglia et al. (2016)	Small worldness - via lagged linear coherence	Delta (2-4), theta (4-8), alpha1 (8-10.5), alpha2 (10.5-13), beta1 (13-20), beta2 (20-30), gamma (30-45)	EC 5min; EO 5min			eLORETA	2,000	MCI, AD	73

Miraglia et al. (2023)	Small world index, modularity, global efficiency - via lagged linear connectivity	Delta (2-4), theta (4-8), alpha1 (8-10.5), alpha2 (10.5-13), beta1 (13-20), beta2 (20-30), gamma (30-45)	EC, 5min; EO, 5min; Hypervent., 5min		eLORETA	2,000	MCI	74
Peraza et al. (2018)	Maximum betweenness centrality, diameter, eccentricity, radius, maximum degree, leaf ratio, PLI mean, PLI leaf, PLI root, PLI height - via phase lag index	Delta (0.5-4), theta1 (4-5.5), theta2 (5.5-8), alpha (8-13), beta (13-30), dominant frequency (+/- 2)	EC, 2.5min	NA		2,000	AD	81
Požar et al. (2020)	Clustering coefficient, characteristic path length, small worldness, degree, betweenness centrality, eccentricity, divergence, diameter, leaf fraction, hierarchy - via phase lag index	Delta (0.5-4), theta (4-8), alpha1 (8-10), alpha2 (10-13), beta (13-30)	EC, ≥3min	Avg regions		2,000	MCI	83
Smith et al. (2016)	Local clustering coefficient, degree variance for cluster span threshold, union of shortest paths - via weighted phase lag index	Beta (12.2-32)	EC, ≥5min	NA		5,000	AD	92
Tahaei et al. (2012)	Synchronizability, eigenratios - via Pearson cross-correlation coefficients	Delta (1-3), theta (3-7), alpha (7-13), beta (13-30), gamma (30-40)	EC, 3-4min	NA		1,000	AD	97

Tait et al. (2019)	Mean degree, small worldness, clustering coefficient, characteristic path length, closeness centrality - via phase locking factor	Delta (1-4), theta (4-8), alpha (8-13), beta (13-30), gamma (30-45)	EO, NA		eLORETA	20,000	AD	98
Vecchio et al. (2014)	Characteristic path length, clustering coefficient, small worldness - via lagged linear coherence	Delta (2-4), theta (4-8), alpha1 (8-10.5), alpha2 (10.5-13), beta1 (13-20), beta2 (20-30), gamma (30-45)	EC, ≥5min		eLORETA, sLORETA	2,000	MCI, AD	105
Vecchio et al. (2016)	Small worldness - via lagged linear coherence	Delta (2-4), theta (4-8), alpha1 (8-10.5), alpha2 (10.5-13), beta1 (13-20), beta2 (20-30), gamma (30-45)	EC, ≥5min		eLORETA, sLORETA	2,000	MCI, AD	106
Vecchio et al. (2017)	Small worldness - via lagged linear coherence	Delta (2-4), theta (4-8), alpha1 (8-10.5), alpha2 (10.5-13), beta1 (13-20), beta2 (20-30), gamma (30-45)	EC, ≥5min		eLORETA, sLORETA	2,000	AD	107
Vecchio et al. (2018)	Small worldness - via lagged linear coherence	Delta (2-4), theta (4-8), alpha1 (8-10.5), alpha2 (10.5-13), beta1 (13-20), beta2 (20-30), gamma (30-45)	EC, ≥5min		eLORETA, sLORETA	2,000	MCI, AD	108
Vecchio et al. (2021)	Small worldness - via lagged linear coherence	Delta (2-4), theta (4-8), alpha1 (8-10.5), alpha2 (10.5-13), beta1 (13-20), beta2 (20-30)	EC, ≥6min		eLORETA	2,000	MCI, AD	109
Vecchio et al. (2022)	Small worldness - via lagged linear coherence	Delta (2-4), theta (4-8), alpha1 (8-10.5), alpha2 (10.5-13), beta1 (13-20), beta2 (20-30), gamma (30-45)	EC, ≥5min		eLORETA	2,000	AD	110

Wang et al. (2014)	Degree, clustering coefficient, avg path length, betweenness, global efficiency, local efficiency, small worldness - via magnitude squared coherence	Delta (0.5-4), theta (4-7), alpha1 (8-10), alpha2 (10-12), beta (13-30), gamma (30-40)	EC, 10min		NA	8,000	AD	112
Wei et al. (2015)	Characteristic path length, clustering coefficient, small worldness - via phase synchrony index	Alpha (8-16), beta (16-32)		Attention (color matching)	Pairs	200	MCI	115
Xu et al. (2014)	Clustering coefficient, path length - via magnitude squared coherence	Theta (4-8), alpha (8-13), beta (13-30), gamma (30-40), full band (4-60)	EC, ≅20min		NA	2,000	MCI	117
Youssef et al. (2021)	Avg node degree, normalized avg clustering coefficient, normalized characteristic path length, avg local & global efficiency, small worldness, maximum degree, betweenness centrality, eccentricity, diameter, leaf fraction, tree hierarchy - via debiased weighted phase lag index	Delta (1-4), theta (4-7), alpha1 (8-10), alpha2 (10-13), beta (13-30), gamma (30-45)	EC, 5min†		NA	4,000	MCI	119
Yu et al. (2018)	Global efficiency, clustering coefficient, small worldness - via permutation disalignment index	Broadband (0.5-30)	EC, 10min		Pairs	8,000	AD	120

Yu et al. (2020)	Clustering coefficient, global and local efficiency, nodal between-ness, edge betweenness - via phase synchron-ization index	Broadband (0.5-30)	EC, EO, 10min total		Pairs		8,000	AD	121

4. Miscellaneous Methods

Authors (Year)	Connectivity Method or Type	Frequencies Analyzed (Hz)	Rest Type, Duration	Active Task Type	Sensor-Level Approach	Source Space Approach	Segment Duration (ms)	Clinical Groups	Ref.
Babiloni et al. (2009a)	Directed transfer function	Delta (1-4), theta (4-8), alpha1 (8-10), alpha2 (10-12), beta1 (13-20), beta2 (20-30), gamma (30-40)	EC, 5min		Avg regions (frontal-parietal; left-right)		NA	MCI, AD	3
Birba et al. (2022)	Weighted symbolic mutual information	4-10	NA, 10min		Pairs, clusters		1,000	AD	11
Blinowska et al. (2017)	Directed transfer function	Delta (1-4), theta (4-8), alpha (8-13), beta (13-30), gamma (30-45)	EC, NA		Pairs		2,000	AD	12
Bonanni et al. (2021)	Mutual information analysis	Broadband (1-100)	EC, NA		Pairs, avg regions		2,000	AD	13
Cantero et al. (2009b)	Directed transfer function	Alpha1 (7.5-10), alpha2 (10.1-12.5)	EC, NA			swLORETA	NA	MCI	17
Chan et al. (2013)	Cross mutual information	Broadband (0.5-70)	EC, 2 min EO, 10 sec PS‡, 10 sec		Pairs		10,000	AD	20
Crook-Rumsey et al. (2022)	Spiking Neural Network	Broadband (0.01-35)		WM, memory	Avg regions		1,000 (full epoch)	MCI	22
Ding et al. (2022)	Correlation coefficient, cross-power spectral density correlation coefficient	Delta (1-4), theta (4-8), alpha (8-12), beta1 (12-18.5), beta2 (18.5-21), beta3 (21-30)	EC, 5min		Pairs		15,000	MCI, AD	25

Guo et al. (2021)	Power envelope connectivity	Delta (1-4), theta (5-7), alpha (8-12), beta (13-30), gamma (31-45)	EC, 8min			Custom	2,000	MCI	40
Herzog et al. (2022)	Dual total correlation	Delta (0.5-4), theta (4-8), alpha (8-12), beta (12-30), gamma (30-40)	EC, 5min			sLORETA	NA	AD	45
Jeong et al. (2001)	Cross mutual information	Broadband (1-35)	EC, 16sec		Pairs		16,000	AD	52
Kim et al. (2018)	Time-delayed mutual information analysis; KL decomposition	Broadband (1-35)	EC, 33sec		Pairs		512	AD	58
Knyazeva et al. (2013)	Synchronization via S-estimator	Broadband (1-50)	EC, 3-4min			LAURA	1,000	AD	60
Koenig et al. (2005)	Global field synchronization	A, B: Delta (1-4), theta (4.5-7.5), alpha (8-12), beta (12.5-30), A: gamma (35-45)	A: EC, 20min; B: EC, ≥20sec		Global (19 sites)		2,000	MCI, AD	61
Lee et al. (2010)	Global field synchronization	Delta (1-3), theta (4-7), alpha (8-12), beta1 (13-18), beta2 (19-21), beta3 (22-30), gamma (31-50)	EC, 10min		Global (18 sites)		2,000	AD	64
Li et al. (2021)	Directed transfer function	Theta (NA)	EC, 5min		NA		1,000	MCI	67
Liu et al. (2012)	Cross-mutual information	Delta (1-4), theta (4-7), alpha (7-13), beta (13-25), gamma (25-50)		Auditory oddball	Pairs		6,000	MCI	68
Mohaved et al. (2022)	Synchronization likelihood	Theta (4-8), alpha (8-13), beta (13-32)	EC, 30min		Pairs		60,000	MCI	75
Núñez et al. (2019)	Amplitude envelope correlation	Delta (1-4), theta (4-8), alpha (8-13), beta1 (13-19), beta2 (19-30)	EC, 5min		Avg regions		15,000	MCI, AD	78
Núñez et al. (2021)	Instantaneous amplitude correlation	Delta (1-4), theta (4-8), alpha (8-13), beta1 (13-19), beta2 (19-30)	EC, 5min			sLORETA	NA	MCI, AD	79

Park et al. (2008)	Global field synchronization	Delta (1-3), theta (4-7), alpha (8-12), beta1 (13-18), beta2 (19-21), beta3 (22-30), gamma (30-50), full (1-70)	EC, EO, 10min (EC used)		Global (18 sites)		2,000	AD	80
Ruiz-Gomez et al. (2019a)	Amplitude envelope correlation	Delta (1-4), theta (4-8), alpha (8-13), beta1 (13-19), beta2 (19-30), gamma (30-70)	EC, 5min		Pairs		5,000	AD	85
Ruiz-Gomez et al. (2019b)	Amplitude envelope correlation, synchronization likelihood	Delta (1-4), theta (4-8), alpha (8-13), beta1 (13-19), beta2 (19-30), gamma (30-70)	EC, 5min		Pairs		5,000	MCI, AD	86
Ruiz-Gomez et al. (2021)	Global strength, characteristic path length, clustering coefficient - via phase lag index and canonical correlation analysis	Delta (1-4), theta (4-8), alpha (8-13), beta1 (13-19), beta2 (19-30), gamma (30-70)	EC, 5min			sLORETA	5,000	MCI, AD	87
Sedghizadeh et al. (2022)	Amplitude, phase-amplitude	A: low gamma (35-45); PA: theta (4-8), gamma (39-41)		Olfactory oddball	Fz, Cz, Pz		3,000	MCI, AD	91
Song et al. (2018)	Generalized composite multiscale entropy vector	Broadband (0.5-40)	EC, 1min		NA		8,000	AD	93
Tahaei et al. (2012)	Synchronization	Delta (1-3), theta (3-7), alpha (7-13), beta (13-30), gamma (30-40)	EC, 3-4min		NA		1,000	AD	97
Timothy et al. (2017)	Recurrence rate from cross recurrence quantification analysis	Broadband (0.4-60)	EC, 5min	STM	Avg regions		10,000	MCI	101
Tyrer et al. (2020)	Dynamic causal modeling	Broadband (2-30)		Memory, visual priming		MSP	552	AD	103
Vanneste et al. (2021)	Phase-amplitude cross-frequency coupling	Theta (4-7.5), gamma (30.5-44.5)	EC, 5min			eLORETA	NA	MCI	104

Vysata et al. (2015)	Mutual information	Five wavelet scales	EC, 20min	Pairs	60-80K	AD	111
Wen et al. (2014)	Global synchronization index; Global coupling index	Delta (1-3), theta (4-7), alpha (8-12), beta1 (13-18), beta2 (19-21), beta3 (22-30), gamma (31-50)	EC, 8min	Global (10 sites)	10,000	MCI	116
Yu et al. (2018)	Permutation disalignment index	Broadband (0.5-30)	EC, 10min	Pairs	8,000	AD	120
Zhao et al. (2019)	Revised orthogonal least squares	Broadband (>2)	EC, EO, 30min total	Pairs	4,000	AD	123

Notes: *individualized landmarks; ^oddball trials, post-stim; ~frequent trials only; † pre- and post-task; ‡ passive visual task; §repeated within 1-year interval; avg = averaged; DRM = Deese-Roediger-McDermott word recognition task; EC = eyes closed; EMDPL = empirical mode decomposition phase locking; EO = eyes open; Hypervent = hyperventilation; NA = not available/specified; PS = photic stimulation; Ref = reference number; STM = short-term memory; WM = working memory.

Table S4.
Quality assessment of each study included in the review, based on the modified Newcastle-Ottawa Scale.

	Selection											Compar-ability			Exposure							
	Definition of MCI/AD			Representative-ness of cases		Selection of controls			Definition of controls			Match/ control			Connectivity analysis			Same method		Missing/ excluded EEG data		
Ref. Authors (Year)	*Diagnostic criteria	Records or customized criteria	No description	*Consecutive	Non-consecutive/No Description	*Community	Hospital/Clinic	Not described	*No objective cognitive deficits (1/2)	*No subjective cognitive deficits (1/2)	Not defined	*Age	*Educ	*Sex	*Blinded	Not blinded	No description	*Yes	No	*Comparable for all groups	No statistics, but described	Different amounts or no description
1. Afshari & Jalili (2016)	X				X			X			X						X	X				X
2. Al-Nuaimi et al. (2021)	X				X		X		X								X	X				X
3. Babiloni et al. (2009a)	X				X			X	X			X	X	X	X			X				X
4. Babiloni et al. (2009b)	X				X			X	X			X	X		X			X			X	
5. Babiloni et al. (2016)	X				X			X	X			X	X	X	X			X				X
6. Babiloni et al. (2018a)	X				X			X	X			X	X	X			X	X				X
7. Babiloni et al. (2018b)	X				X		?		X			X	X	X	X			X				X
8. Babiloni et al. (2019)	X				X		?		X	X		X	X	X	X			X		X		
9. Bagattini et al. (2022)	X				X			X	X			X	X				X	X		X		
10. Barzegaran et al. (2016)	X				X	X			X	X		X	X				X	X			X	
11. Birba et al. (2022)	X				X	X			X			X	X				X	X				X
12. Blinowska et al. (2017)	X			X				X	X						X			X				X

13. Bonanni et al. (2021)	X			X				X	X			X	X	X	X			X		X		
14. Cai et al. (2018)	X				X			X	X			X					X	X		X		
15. Cai et al. (2020)		X			X		X		X								X	X		X		
16. Cantero et al. (2009a)	X				X	X			X	X		X	X				X	X		X		
17. Cantero et al. (2009b)	X				X	X			X	X		X	X				X	X		X		
18. Canuet et al. (2012)	X				X			X			X			X	X			X			X	
19. Cecchetti et al. (2021)	X			X		X			X			X	X	X	X			X				X
20. Chan et al. (2013)	X				X			X	X			X	X	X			X	X				X
21. Choi et al. (2021)	X			X		X					X		X				X	X		X		
22. Crook-Rumsey et al. (2022)	X				X			X	X			X		X			X	X				X
23. Das & Puthankattil (2020)		X			X			X			X						X	X				X
24. Dattola et al. (2021)			X		X			X			X						X	X				X
25. Ding et al. (2022)	X				X		X				X						X	X			X	X
26. Duan et al. (2020)		X			X			X			X						X	X		X		
27. Dubovik et al. (2013)	X				X			X	X			X		X			X	X				X
28. Engels et al. (2015)	X				X			X	X			X	X	X			X	X		X		
29. Escudero et al. (2016)	X				X			X	X			X					X	X			X	
30. Fide et al. (2022)	X				X			X	X			X					X	X				X
31. Fide et al. (2023)	X				X	X	X				X	X	X	X			X	X				X
32. Franciotti et al. (2019)		X			X			X	X								X	X			X	
33. Franciotti et al. (2022)	X			X				X	X			X	X	X			X	X		X		
34. Frangopoulou et al. (2022)			X		X			X			X	X					X	X				X
35. Frantzidis et al. (2014)	X				X			X	X			X		X			X	X			X	
36. Gómez et al. (2018)		X			X			X	X			X		X			X	X		X		
37. Gonzalez-Escamilla et al. (2014)	X				X	X	X		X				X				X	X		X		
38. Gonzalez-Escamilla et al. (2015)	X				X	X	X		X				X				X	X		X		
39. Guntekin et al. (2008)	X			X				X			X						X	X				X
40. Guo et al (2021)	X			X				X	X					X			X	X				X
41. Gurja et al. (2022)	X				X			X	X			X	X	X			X	X				X

42. Han et al. (2017)	X				X			X	X			X	X	X		X	X			X
43. Handayani et al. (2018)			X		X			X	X			X		X		X	X			X
44. Hata et al. (2016)	X			X			X		X							X	X	X		
45. Herzog et al. (2022)	X				X			X			X	X	X			X	X			X
46. Hidasi et al. (2007)	X				X			X	X							X	X			X
47. Ho et al. (2014)			X		X			X			X					X	X	X		
48. Ioulietta et al. (2020)	X				X		X		X	X						X	X			X
49. Jalili et al. (2016)	X				X			X	X							X	X			X
50. Jalili (2017)			X		X		X				X					X	X			X
51. Jelic et al. (1997)	X			X				X	X			X	X	X		X	X			X
52. Jeong et al. (2001)	X				X			X	X			X		X		X	X			X
53. Jiang (2005)	X				X	X			X			X		X		X	X	X		
54. Jiang et al. (2006)	X				X	X			X			X		X		X	X	X		
55. Jiang et al. (2008)	X				X	X			X			X		X		X	X	X		
56. Josefsson et al. (2019)			X		X			X			X					X	X			X
57. Kabbara et al. (2018)			X		X	X	X				X					X	X	X		
58. Kim et al. (2018)			X		X	X			X							X	X			X
59. Knyazeva et al. (2010)	X				X	X			X							X	X			X
60. Knyazeva et al. (2013)	X				X			X	X	X		X	X			X	X		X	
61. Koenig et al. (2005)	X			X		X	X		X	X						X	X			X
62. La Foresta et al. (2019)			X		X			X			X					X	X		X	
63. Lazarou et al. (2022)	X				X		X		X	X		X	X	X		X	X			X
64. Lee et al. (2010)	X				X	X	X		X			X	X		X		X		X	
65. Leuchter et al. (1994)	X				X			X	X							X	X	X		
66. Li et al. (2019)		X			X	X			X				X	X		X	X		X	
67. Li et al. (2021)		X			X		X		X			X	X			X	X			X
68. Liu et al. (2012)	X				X			X	X			X		X		X	X			X
69. Locatelli et al. (1998)	X				X			X			X					X	X	X		
70. Mammone et al. (2019)	X				X		X				X					X	X		X	

71. Mehraram et al. (2020)	X					X			X			X			X			X	X			X
72. Michels et al. (2017)	X					X			X			X	X	X	X			X	X	X		
73. Miraglia et al. (2016)	X					X		X		X								X	X			X
74. Miraglia et al. (2023)	X					X			X	X								X	X			X
75. Mohaved et al. (2022)	X					X			X	X								X	X	X		
76. Musaeus et al. (2019a)	X			X			X	X		X	X		X	X	X	X			X	X		
77. Musaeus et al. (2019b)	X			X				X		X	X		X	X	X	X			X			X
78. Núñez et al. (2019)	X					X			X	X			X	X	X			X	X			X
79. Núñez et al. (2021)	X					X			X	X					X			X	X		X	
80. Park et al. (2008)	X					X	X	X		X			X	X		X			X	X		
81. Peraza et al. (2018)		X				X			X	X			X		X	X			X	X		
82. Po et al. (2010)	X					X			X	X	X		X					X	X			X
83. Požar et al. (2020)		X				X	X			X			X	X				X	X		X	
84. Rodikaia, et al. (2022)	X					X		X				X	X	X	X			X	X			X
85. Ruiz-Gomez et al. (2019a)	X					X			X			X						X	X			X
86. Ruiz-Gomez et al. (2019b)	X					X			X	X			X					X	X			X
87. Ruiz-Gomez et al. (2021)	X					X			X	X								X	X		X	
88. Sankari et al. (2011)	X					X			X			X						X	X			X
88. Sankari et al. (2012)	X					X			X			X						X	X			X
90. Sedghizadeh et al. (2020)	X					X		X		X			X					X	X			X
91. Sedghizadeh et al. (2022)	X					X			X	X				X	X			X	X			X
92. Smith et al. (2016)	X					X			X	X			X					X	X		X	
93. Song et al. (2018)			X			X			X	X								X	X	X		
94. Spyrou et al. (2018)	X					X	X			X			X	X	X			X	X	X		
95. Su et al. (2021)	X					X	X			X								X	X			X
96. Sweeney-Reed et al. (2012)	X					X	X			X			X	X	X			X	X			X
97. Tahaei et al. (2012)	X					X			X	X			X	X				X	X			X
98. Tait et al. (2019)	X			X				X		X								X	X	X		
99. Tao et al. (2006)	X					X		X				X	X		X			X	X			X

100. Teipel et al. (2009)	X				X			X	X	X				X	X	X		X	
101. Timothy et al. (2017)	X				X			X			X	X			X	X	X		
102. Tóth et al. (2014)	X				X			X			X	X			X	X	X		
103. Tyrer et al. (2020)	X				X			X	X						X	X			X
104. Vanneste et al. (2021)		X			X			X	X			X		X	X	X			X
105. Vecchio et al. (2014)	X				X			X	X			X	X	X	X	X	X		
106. Vecchio et al. (2016)	X				X			X	X			X			X	X		X	
107. Vecchio et al. (2017)	X				X			X	X			X			X	X			X
108. Vecchio et al. (2018)	X				X			X	X						X	X			X
109. Vecchio et al. (2021)	X				X			X	X						X	X		X	
110. Vecchio et al. (2022)	X				X			X			X				X	X			X
111. Vysata et al. (2015)	X				X			X	X			X			X	X		X	
112. Wang et al. (2014)			X		X			X	X			X			X	X			X
113. Wang et al. (2015)	X				X			X	X						X	X			X
114. Wang et al. (2022)	X				X		X		X				X	X	X	X			X
115. Wei et al. (2015)		X			X	X			X			X	X		X	X			X
116. Wen et al. (2014)	X				X	X			X			X	X	X	X	X		X	
117. Xu et al. (2014)	X				X		X		X	X		X	X	X	X	X	X		
118. Yan et al. (2021)	X			X			X		X			X		X	X	X	X		
119. Youssef et al. (2021)	X				X		X		X	X		X	X	X	X	X	X		
120. Yu et al. (2018)	X				X			X	X			X		X	X	X		X	
121. Yu et al. (2020)	X				X			X	X						X	X			X
122. Zhang et al. (2022)	X			X			X		X			X	X	X	X	X			X
123. Zhao et al. (2019)	X				X	X			X			X		X	X	X	X		
124. Zheng et al. (2007)	X				X	X			X			X		X	X	X	X		

Notes: Ref. = reference number; * = 'high quality' indicators.
